# Esterase-Sensitive Prodrugs of a Potent Bisubstrate Inhibitor of Nicotinamide *N*-Methyltransferase (NNMT) Display Cellular Activity

**DOI:** 10.3390/biom11091357

**Published:** 2021-09-14

**Authors:** Matthijs J. van Haren, Yongzhi Gao, Ned Buijs, Roberto Campagna, Davide Sartini, Monica Emanuelli, Lukasz Mateuszuk, Agnieszka Kij, Stefan Chlopicki, Pol Escudé Martinez de Castilla, Raymond Schiffelers, Nathaniel I. Martin

**Affiliations:** 1 Biological Chemistry Group, Institute of Biology Leiden, Leiden University, Sylviusweg 72, 2333 BE Leiden, The Netherlands; y.gao@biology.leidenuniv.nl (Y.G.); n.p.buijs@biology.leidenuniv.nl (N.B.); 2 Department of Clinical Sciences, Universitá Politecnica delle Marche, Via Ranieri 65, 60131 Ancona, Italy; rob_campagna@yahoo.com (R.C.); d.sartini@staff.univpm.it (D.S.); m.emanuelli@staff.univpm.it (M.E.); 3 Jagiellonian Centre for Experimental Therapeutics (JCET), Jagiellonian University, Bobrzynskiego 14, 30-348 Krakow, Poland; lukasz.mateuszuk@jcet.eu (L.M.); agnieszka.kij@jcet.eu (A.K.); stefan.chlopicki@jcet.eu (S.C.); 4 Faculty of Medicine, Chair of Pharmacology, Jagiellonian University Medical College, Grzegorzecka 16, 31-531 Krakow, Poland; 5 Central Diagnostic Laboratory, Division Laboratories, Pharmacy and Biomedical Genetics (LAB), Universitair Medisch Centrum Utrecht, Heidelberglaan 100, 3584 CX Utrecht, The Netherlands; P.Castilla@umcutrecht.nl (P.E.M.d.C.); R.Schiffelers@umcutrecht.nl (R.S.)

**Keywords:** NNMT, inhibition, prodrug, cell permeability, esterase, cellular activity

## Abstract

A recently discovered bisubstrate inhibitor of Nicotinamide *N*-methyltransferase (NNMT) was found to be highly potent in biochemical assays with a single digit nanomolar IC_50_ value but lacking in cellular activity. We, here, report a prodrug strategy designed to translate the observed potent biochemical inhibitory activity of this inhibitor into strong cellular activity. This prodrug strategy relies on the temporary protection of the amine and carboxylic acid moieties of the highly polar amino acid side chain present in the bisubstrate inhibitor. The modification of the carboxylic acid into a range of esters in the absence or presence of a trimethyl-lock (TML) amine protecting group yielded a range of candidate prodrugs. Based on the stability in an aqueous buffer, and the confirmed esterase-dependent conversion to the parent compound, the isopropyl ester was selected as the preferred acid prodrug. The isopropyl ester and isopropyl ester-TML prodrugs exhibit improved cell permeability, which also translates to significantly enhanced cellular activity as established using assays designed to measure the enzymatic activity of NNMT in live cells.

## 1. Introduction

Nicotinamide *N*-methyltransferase (NNMT) is a small molecule methyltransferase enzyme responsible for the conversion of nicotinamide (NA, vitamin b3) to 1-methylnicotinamide (MNA). NNMT utilizes the cofactor *S*-adenosyl-l-methionine (SAM) as a methyl donor, which is converted to *S*-adenosyl-l-homocysteine (SAH) upon methylation of nicotinamide [1]. Under normal physiological conditions, NNMT is mainly expressed in the liver and in adipose tissue [2]. One of the primary roles of NNMT is the detoxification of xenobiotics. This function is achieved through NNMT’s broad substrate recognition that allows for the methylation of different metabolites, including pyridines, quinolines, and other related heterocyclic aromatics [3].

The overexpression of NNMT has been described in a wide variety of tissues and diseases, generally with detrimental effects, although there are reports suggesting a beneficial effect from the NNMT-MNA pathway [4,5,6,7]. Most studies on NNMT focus on its role in the pathology of cancer where elevated NNMT activity has been correlated with tumor aggressiveness and is proposed to promote migration, invasion, and proliferation, leading to its potential as a biomarker predictive of worsened clinical outcomes [8,9,10,11,12,13,14,15]. The overexpression of NNMT has been shown to cause depletion of the cellular pool of SAM, distorting the SAM/SAH balance, subsequently leading to a hypomethylated state with downstream effects on gene expression associated with tumor growth and metastasis [16]. This process is supported by recent proteomics-based studies revealing NNMT to be the master regulator of the differentiation of cancer-associated fibroblasts (CAFs) [17]. In another recent investigation, upregulated MNA levels in the tumor microenvironment were found to lead to the inhibition of T-cell functions, resulting in their deregulated killing capacity and increased tumor growth [18].

**Figure 1:**
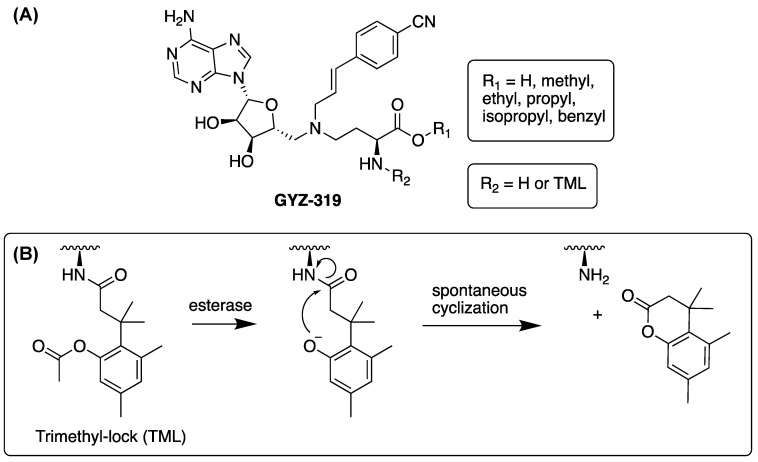
(**A**) Prodrug strategy of compound GYZ-319. The carboxylic acid can be masked as an ester and the amine can be masked as an amide using the esterase-sensitive trimethyl-lock (TML). (**B**) The mechanism of the trimethyl-lock cleavage. Deacetylation by esterases results in spontaneous lactonization, releasing the free amine.

Potent, selective, and cell-active NNMT inhibitors are valuable tools to probe the complex regulatory functions mediated by NNMT and to also investigate a number of different pharmacological hypotheses that suggest NNMT as a therapeutic target. While the growing number of reports connecting NNMT to disease has resulted in an increase in the development of inhibitors of NNMT, to date, very few cell-active inhibitors have been described. In this regard, the bisubstrate NNMT inhibitors pioneered by our group and others exhibit very potent enzyme inhibition in biochemical assays, but given their polar nature, such compounds show limited cellular activity [19,20,21,22,23]. Recent work in our group identified compound GYZ-319 (Figure 1A), which exhibits an IC_50_ value of 3.7 nM, making it one of the most potent NNMT inhibitors reported to date [24]. However, when tested against a range of human cancer cell lines, compound GYZ-319 only showed significant antiproliferative effects at the highest concentration tested of 100 µM, more than four orders of magnitude higher than the concentration needed for enzyme inhibition in the biochemical assay. This absence of cellular activity for GYZ-319 is likely associated with the compound’s poor cell permeability, which is due to the presence of two highly polar functional groups present in all potent bisubstrate NNMT inhibitors reported to date: namely the carboxylic acid and amine moieties of the amino acid motif. Notably, previous structure activity relationship studies of the bisubstrate inhibitors revealed that both the carboxylic acid and amine moieties are required for potent inhibitory activity and attempts at replacing them with less polar bio-isosteres resulted in all cases in a significant loss of potency [19,20,21,22,23].

We, here, report our efforts in developing a prodrug form of compound GYZ-319 with the aim of improving its cellular activity (Figure 1). The carboxylic acid moiety was converted into a variety of esters, which can be cleaved by cellular esterases. In the case of the amino group, a different prodrug strategy was applied. The derivatization of amines to give amides has not been widely used as a prodrug strategy due to the high chemical stability of amide linkages [25,26] and the lack of amidase enzymes necessary for hydrolysis [27]. To circumvent these problems, the trimethyl-lock (TML) moiety [28] was selected as the amine masking motif in the prodrug design (Figure 1B). Upon esterase-mediated hydrolysis of the acetyl group in the TML moiety, the liberated hydroxyl group spontaneously cyclizes to form the corresponding lactone ring, with concomitant release of the free amine. Using these strategies, a series of prodrugs were prepared in which either one, or both, of the ester and TML-groups were incorporated.

To investigate the most suitable prodrug form of compound GYZ-319, the different ester and TML modified analogues were first evaluated for their hydrolytic stability in a buffer after which the most stable prodrugs were evaluated for the esterase-mediated release of the parent compound. After confirming the esterase-mediated conversion to the active compound, the prodrugs were evaluated for cellular activity in a range of cellular assays and compared to the activity of the parent compound.

## 2. Experimental Procedures

All reagents employed were of American Chemical Society grade or finer and were used without further purification unless otherwise stated. For compound characterization, ^1^H NMR spectra were recorded at 400 and 500 MHz with chemical shifts reported in parts per million downfield relative to CHCl_3_ (7.26) or CH_3_OH (δ 3.31).^1^H NMR data are reported in the following order: multiplicity (s, singlet; d, doublet; t, triplet; q, quartet; and m, multiplet), coupling constant (*J*) in hertz (Hz), and the number of protons. Where appropriate, the multiplicity is preceded by br, indicating that the signal was broad. ^13^C NMR spectra were recorded at 101 or 126 MHz with chemical shifts reported relative to CHCl_3_ (77.16) or CH_3_OH (δ 49.00). High-resolution mass spectrometry (HRMS) analysis was performed using a Q-TOF instrument. Purity was confirmed to be ≥95% by LCMS performed on a Shimadzu LC-20AD system (Shimadzu, ’s-Hertogenbosch, The Netherlands) with a Shimadzu Shim-Pack GISS-HP C18 column (3.0 × 150 mm, 3 μm particle size, Shimadzu, ’s-Hertogenbosch, The Netherlands) at 30 °C and equipped with a UV detector monitoring at 214 and 254 nm. The following solvent system, at a flow rate of 0.5 mL/min, was used: solvent A, 0.1% formic acid in water; solvent B, acetonitrile. Gradient elution was as follows: 95:5 (A/B) for 2 min, 95:5 to 0:100 (A/B) over 13 min, 0:100 (A/B) for 2 min, then reversion back to 95:5 (A/B) over 1 min, 95:5 (A/B) for 2 min. This system was connected to a Shimadzu 8040 triple quadrupole mass spectrometer (ESI ionization, Shimadzu, ’s-Hertogenbosch, The Netherlands). The compounds were purified via preparative HPLC performed on a BESTA-Technik system with a Dr. Maisch Reprosil Gold 120 C18 column (25 × 250 mm, 10 μm particle size, Dr.Maisch, Ammerbuch-Entringen, Germany) and equipped with an ECOM Flash UV detector monitoring at 214 nm. The following solvent system, at a flow rate of 12 mL/min, was used: solvent A: 0.1% TFA in water/acetonitrile 95/5; solvent B: 0.1% TFA in water/acetonitrile 5/95. Gradient elution was as follows: 95:5 (A/B) for 5 min, 95:5 to 0:100 (A/B) over 40 min, 0:100 (A/B) for 5 min, then reversion back to 95:5 (A/B) over 2 min, 95:5 (A/B) for 8 min. HRMS analyses were performed on a Shimadzu Nexera X2 UHPLC system (Shimadzu, ’s-Hertogenbosch, The Netherlands) with a Waters Acquity HSS C18 column (2.1 × 100 mm, 1.8 μm particle size, Waters Chromatography, Etten-Leur, The Netherlands) at 30 °C and equipped with a diode array detector. The following solvent system, at a flow rate of 0.5 mL/min, was used: solvent A, 0.1% formic acid in water; solvent B, 0.1% formic acid in acetonitrile. Gradient elution was as follows: 95:5 (A/B) for 1 min, 95:5 to 15:85 (A/B) over 6 min, 15:85 to 0:100 (A/B) over 1 min, 0:100 (A/B) for 3 min, then reversion back to 95:5 (A/B) for 3 min. This system was connected to a Shimadzu 9030 QTOF mass spectrometer (ESI ionization, Shimadzu, ’s-Hertogenbosch, The Netherlands) calibrated internally with Agilent’s API-TOF reference mass solution kit (5.0 mM purine, 100.0 mM ammonium trifluoroacetate and 2.5 mM hexakis(1H,1H,3H-tetrafluoropropoxy)phosphazine) diluted to achieve a mass count of 10,000.

Compounds **2c** [29], **2d** [30], **2e** [31], **3c** [32], **3d** [30], **4a** [19], **4b** [33], **4f** [34], **5a** [21] were prepared as previously described and had NMR spectra and mass spectra consistent with the assigned structures.

### 2.1. Synthetic Procedures

*(S)-3-((tert-butoxycarbonyl)amino)-4-isopropoxy-4-oxobutanoic acid* (**3e**). To a stirred suspension of 4-benzyl 1-*iso*propyl (*tert*-butoxycarbonyl)-L-aspartate (**2e**) (1200 mg, 3.3 mmol), 10% Pd-C (120 mg) under H_2_ atmosphere. After completion of the reaction (TLC), the mixture was filtered through celite, and the filtrate was concentrated under a vacuum to get **3e** (900 mg, 99% yield) as a colorless oil. ^1^H NMR (400 MHz, CDCl_3_) δ 5.55 (d, *J* = 8.4 Hz, 1H), 5.11–5.03 (m, 1H), 4.54–4.50 (m, 1H), 3.05 (dd, *J* = 17.4, 4.5 Hz, 1H), 2.99 (s, 2H), 2.91 (s, 2H), 2.85 (dd, *J* = 17.3, 4.3 Hz, 1H), 1.46 (s, 9H), 1.25 (dd, *J* = 10.2, 6.3 Hz, 6H).^13^C NMR (101 MHz, CDCl_3_) δ 176.1, 170.5, 155.6, 80.3, 69.7, 50.0, 36.7, 28.3, 21.6. HRMS (ESI): calculated for C_12_H_22_NO_6_ [M + H]^+^ 276.1447, found 276.1450.

*Ethyl-N^2^-(tert-butoxycarbonyl)-N^4^-methoxy-N^4^-methyl-L-asparaginate* (**4c**). To a stirred suspension of **3c** (100 mg, 0.4 mmol) in 10 mL CH_2_Cl_2_, benzotriazol-1-yloxytris(dimethylamino)phosphonium hexafluorophosphate (BOP) (194 mg, 0.44 mmol) and 0.1 mL Et_3_N were added and after 10 min *N*,*O*-dimethylhydroxylamine hydrogen chloride (43 mg, 0.44) was added followed by another 0.1 mL Et_3_N. The resulting mixture was stirred at room temperature for 2 h, 10 mL water was added to quench the reaction, the product extracted with CH_2_Cl_2_ (10 mL × 3), the organic layer washed with water, brine, dried over NaSO_4_. The solvent was removed and the crude compound purified by column chromatography to get compound **4c** as a colorless oil (960 mg, 80% yield). ^1^H NMR (400 MHz, CDCl_3_) δ 5.73 (d, *J* = 8.8 Hz, 1H), 4.59–4.55 (m, 1H), 4.24–4.19 (m, 2H), 3.70 (s, 3H), 3.18 (s, 3H), 2.97–2.91 (m, 1H), 1.46 (s, 9H), 1.28 (t, *J* = 7.1 Hz, 3H). LRMS (ESI): calculated for C_13_H_25_N_2_O_6_ [M + H]^+^ 305.17, found 305.19.

*Propyl-N^2^-(tert-butoxycarbonyl)-N^4^-methoxy-N^4^-methyl-L-asparaginate* (4d). Following the procedure described for compound **4c**, coupling compound **3d** (730 mg, 2.6 mmol) with *N*,O-dimethylhydroxylamine hydrogen chloride (284 mg, 2.9 mmol) yielded compound **4d** as a colourless oil (708 mg, 84% yield).^1^H NMR (400 MHz, CDCl_3_) δ 5.70 (d, *J* = 8.9 Hz, 1H), 4.53 (dt, *J* = 9.3, 4.4 Hz, 1H), 4.08–4.01 (m, 2H), 3.66 (s, 3H), 3.21–3.15 (m, 1H), 3.12 (s, 3H), 2.91–2.86 (br m, 1H), 1.67–1.56 (m, 2H), 1.41 (s, 9H), 0.90 (t, *J* = 7.4 Hz, 3H).^13^C NMR (101 MHz, CDCl_3_) δ 171.8, 155.8, 80.6, 67.1, 62.1, 49.1, 34.7, 32.4, 21.9, 4.8. HRMS (ESI): calculated for C_14_H_27_N_2_O_6_ [M + H]^+^ 319.1869, found 318.1873.

*Isopropyl-N^2^-(tert-butoxycarbonyl)-N^4^-methoxy-N^4^-methyl-L-asparaginate* (**4e**). Following the procedure described for compound **4c**, coupling compound **3e** (800 mg, 2.9 mmol) with *N*,O-dimethylhydroxylamine hydrogen chloride (312 mg, 3.2 mmol) yielded compound **4e** as a colourless oil (760 mg, 82% yield). ^1^H NMR (400 MHz, CDCl_3_) δ 5.71 (d, *J* = 8.8 Hz, 1H), 5.11–5.01 (m, 1H), 4.55–4.50 (m, 1H), 3.70 (s, 3H), 3.25–3.18 (m, 1H), 3.17 (s, 3H), 2.95 –2.89 (br m, 1H), 1.46 (s, 10H), 1.25 (dd, *J* = 13.5, 6.3 Hz, 6H). ^13^C NMR (101 MHz, CDCl_3_) δ 171.1, 155.8, 69.1, 61.3, 50.0, 34.7, 32.0, 28.4, 21.7. HRMS (ESI): calculated for C_14_H_27_N_2_O_6_ [M + H]^+^ 319.1869, found 318.1872.

*Methyl-(S)-2-((tert-butoxycarbonyl)amino)-4-oxobutanoate* (**5b**). To a solution of methyl *N*^2^-(*tert*-butoxycarbonyl)-*N*^4^-methoxy-*N*^4^-methyl-L-asparaginate **4b** (1000 mg, 4.1 mmol) in CH_2_Cl_2_ (20 mL) at −78 °C was added DIBAL-H (1M in hexane, 6.0 mL) and the resulting mixture was stirred at −78 °C for 2 h. An amount of 10 mL water was added to quench the reaction, 10 mL 1M HCl (aq) was added to the solution, and the product was extracted with Et_2_O. Organic layer wash was combined with H_2_O, brine, and dried over Na_2_SO_4_. The solvent was removed to yield compound **5b** as a colorless oil used in the next step without further purification.

*Ethyl-(S)-2-((tert-butoxycarbonyl)amino)-4-oxobutanoate* (**5c**). Following the procedure described for compound **5a**, compound ethyl *N*^2^-(*tert*-butoxycarbonyl)-*N*^4^-methoxy-*N*^4^-methyl-L-asparaginate **4c** (560 mg, 1.84 mmol) was reduced using DIBAL-H (1M in hexane, 3 mL) to yield compound **5c,** which was used in the next step without further purification.

*Propyl-(S)-2-((tert-butoxycarbonyl)amino)-4-oxobutanoate* (**5d**). Following the procedure described for compound **5a**, compound propyl *N*^2^-(*tert*-butoxycarbonyl)-*N*^4^-methoxy-*N*^4^-methyl-L-asparaginate **4d** (400 mg, 1.3 mmol) was reduced by DIBAL-H (1M in hexane, 2 mL) to yield compound **5d** which was used in the next step without further purification.

*Isopropyl-(S)-2-((tert-butoxycarbonyl)amino)-4-oxobutanoate* (**5e**). Following the procedure described for compound **5a**, compound *iso*propyl *N*^2^-(*tert*-butoxycarbonyl)-*N*^4^-methoxy-*N*^4^-methyl-L-asparaginate **4e** (450 mg, 1.4 mmol) was reduced by DIBAL-H (1M in hexane, 2 mL) to yield compound **5e** which was used in the next step without further purification.

*Benzyl-(S)-2-((tert-butoxycarbonyl)amino)-4-oxobutanoate* (**5f**). Following the procedure described for compound **5a**, compound benzyl *N*^2^-(*tert*-butoxycarbonyl)-*N*^4^-methoxy-*N*^4^-methyl-L-asparaginate **4f** (370 mg, 1.0mmol) was reduced by DIBAL-H (1M in hexane, 1.2 mL) to yield compound **5f** which was used in the next step without further purification.

*tert-Butyl-N^2^-(3-(2-acetoxy-4,6-dimethylphenyl)-3-methylbutanoyl)-N^4^-methoxy-N^4^-methyl-L-asparaginate* (**8a**). *tert*-butyl *N*^2^-(*tert*-butoxycarbonyl)-*N*^4^-methoxy- *N*^4^-methyl-*L*-asparaginate **4a** (300 mg, 0.9 mmol) in dioxane (5 mL) was selectively deprotected using 4N HCl in dioxanes (10 mL) while stirring for 1 h at 0 °C and 75 min at room temperature. The mixture was concentrated and added to a mixture of TML acid **7** (220 mg, 0.83 mmol, 0.9 eq), Et_3_N (330 µL, 2.4 mmol, 2.8 eq), and BOP (450 mg, 1 mmol, 1.1 eq) in CH_2_Cl_2_ (10 mL). The mixture was stirred overnight, diluted with CH_2_Cl_2_ to 100 mL, washed with saturated NaHCO_3_, water, and brine, dried over sodium sulfate, and concentrated. The crude product was purified by column chromatography (30% EtOAc in petroleum ether, followed by flushing with 20% MeOH in EtOAc) yielding compound **8a** (350 mg, 88%) of an off-white powder. ^1^H NMR (300 MHz, CDCl_3_) δ 6.77 (d, *J* = 2.0 Hz, 1H), 6.56 (d, *J* = 2.1 Hz, 1H), 6.51 (d, *J* = 8.4 Hz, 1H), 4.66 (dt, *J* = 8.4, 4.2 Hz, 1H), 3.62 (s, 3H), 3.11 (s, 3H), 2.98 (dd, *J* = 17.3, 4.2 Hz, 1H), 2.78 (d, *J* = 13.8 Hz, 1H), 2.64–2.41 (m, 5H), 2.30 (s, 3H), 2.19 (s, 3H), 1.61 (s, 3H), 1.54 (s, 3H), 1.40 (s, 9H). ^13^C NMR (75 MHz, CDCl_3_) δ 170.66, 170.19, 170.08, 149.60, 138.14, 136.15, 133.61, 132.47, 123.21, 81.76, 61.15, 49.28, 48.51, 39.54, 34.32, 31.97, 31.64, 27.88, 25.45, 21.92, 20.24. LRMS (ESI): calculated for C_25_H_39_N_2_O_7_ [M + H]^+^ 479.28, found 479.35.

*tert-Butyl-(S)-2-(3-(2-acetoxy-4,6-dimethylphenyl)-3-methylbutanamido)-4-oxobutanoate* (**9a**). Following the procedure described for compound **5a**, compound **8a** (110 mg, 0.23 mmol) was reduced by DIBAL-H (1M in hexane, 0.4 mL) to yield compound **9a**, which was used in the next step without further purification.

*Methyl-(S)-4-((((3aR,4R,6R,6aR)-6-(6-amino-9H-purin-9-yl)-2,2-dimethyl-tetra-ydrofuro[3,4-d][1,3]dioxol-4-yl)methyl)((E)-3-(4-cyanophenyl)allyl)amino)-2-((tert-butoxycarbonyl)amino)butanoate* (**11b**). 4-((E)-3-((((3aR,4R,6R,6aR)-6- (6-amino-9H-purin-9-yl)-2,2-dimethyltetrahydrofuro[3,4-d][1,3]dioxol-4-yl)methyl)amino)prop-1-en-1-yl)benzonitrile **10** (50 mg, 0.11 mmol), **5b** (30, 0.13 mmol), NaBH(OAc)_3_ (36 mg, 0.17 mmol) and AcOH (one drop) were dissolved in 1,2- dichloroethane (DCE, 10 mL) and stirred at room temperature under a N_2_ atmosphere overnight. The reaction was quenched by adding 1 N NaOH (10 mL), and the product was extracted with CH_2_Cl_2_. The combined organic layers were washed with brine and dried over Na_2_SO_4_. The solvent was evaporated, and the crude product was purified by column chromatography (5% MeOH in CH_2_Cl_2_) to give compound **11b** as a white powder (47 mg, 65% yield). ^1^H NMR (400 MHz, CDCl_3_) δ 8.21 (s, 1H), 7.94 (s, 1H), 7.49 (d, *J* = 8.3 Hz, 2H), 7.27 (d, *J* = 8.3 Hz, 2H), 6.60 (s, 2H), 6.35 (d, *J* = 16.0 Hz, 1H), 6.27–6.22 (m, 1H), 6.06 (s, 1H), 5.94 (d, *J* = 8.1 Hz, 1H), 5.45 (d, *J* = 6.2 Hz, 1H), 5.03–4.95 (m, 1H), 4.41–4.30 (m, 2H), 3.64 (s, 3H), 3.23 (d, *J* = 6.0 Hz, 2H), 2.79–2.69 (m, 2H), 2.58–2.54 (m, 2H), 2.00–1.89 (m, 1H), 1.82-1.77 (br m, 1H), 1.58 (s, 3H), 1.38 (br s, 12H). ^13^C NMR (101 MHz, CDCl_3_) δ 173.3, 155.9, 155.6, 153.0, 149.0, 141.3, 140.1, 132.3, 131.0, 126.7, 120.2, 119.0, 114.4, 110.5, 90.7, 85.7, 83.9, 83.3, 56.6, 56.1, 53.6, 52.2, 50.6, 44.8, 29.2, 28.4, 27.2, 25.4. HRMS (ESI): calculated for C_33_H_43_N_8_O_7_ [M + H]^+^ 663.3255, found 663.3262.

*Ethyl-(S)-4-((((3aR,4R,6R,6aR)-6-(6-amino-9H-purin-9-yl)-2,2-dimethyltetrahydrofuro[3,4-d][1,3]dioxol-4-yl)methyl)((E)-3-(4-cyanophenyl)allyl)amino)-2-((tert-butoxycarbonyl)amino)butanoate* (**11c**). Following the procedure described for compound **11b**, coupling compound **10** (50 mg, 0.11 mmol) with **5c** (32 mg, 0.13 mmol) afforded compound **11c** as a white powder (53 mg, 72% yield). ^1^H NMR (400 MHz, CDCl_3_) δ 8.15 (s, 1H), 7.91 (s, 1H), 7.46 (d, *J* = 8.3 Hz, 2H), 7.24 (d, *J* = 8.3 Hz, 2H), 6.58 (s, 2H), 6.32 (d, *J* = 16.0 Hz, 1H), 6.26–6.16 (br, 1H), 6.01 (s, 1H), 5.85 (d, *J* = 8.0 Hz, 1H), 5.39 (t, *J* = 8.2 Hz, 2H), 4.95-4.89 (m, 2H), 4.36–4.17 (m, 2H), 3.74–3.51 (m, 6H), 3.43 (s, 4H), 3.29–3.03 (m, 4H), 2.80–2.52 (m, 6H), 2.49 (s, 5H), 1.83-1.64 (m, 3H), 1.53 (s, 3H), 1.34 (br d, *J* = 7.8 Hz, 15H). ^13^C NMR (101 MHz, CDCl_3_) δ 172.2, 156.1, 155.8, 153.0, 148.9, 141.3, 132.3, 126.7, 120.2, 114.4, 110.4, 90.6, 85.6, 83.9, 83.2, 68.8, 65.0, 59.7, 56.8, 56.0, 52.4, 51.0, 44.8, 29.3, 28.3, 25.4, 21.7. HRMS (ESI): calculated for C_33_H_43_N_8_O_7_ [M + H]^+^677.3411, found 677.3420.

*Propyl-(S)-4-((((3aR,4R,6R,6aR)-6-(6-amino-9H-purin-9-yl)-2,2-dimethyl-tetrahydrofuro[3,4-d][1,3]dioxol-4-yl)methyl)((E)-3-(4-cyanophenyl)allyl)amino)-2-((tert-butoxycarbonyl)amino)butanoate* (**11d**). Following the procedure described for compound **11b**, coupling compound **10** (50 mg, 0.11 mmol) with **5d** (34 mg, 0.13 mmol) afforded compound **11d** as a white powder (46 mg, 60% yield). ^1^H NMR (400 MHz, CDCl_3_) δ 8.18 (s, 1H), 7.93 (s, 1H), 7.48 (d, *J* = 8.3 Hz, 2H), 7.26 (d, *J* = 8.3 Hz, 2H), 6.55 (s, 2H), 6.34 (d, *J* = 15.9 Hz, 1H), 6.27–6.17 (m, 1H), 6.03 (s, 1H), 5.88 (d, *J* = 8.1 Hz, 1H), 5.41 (d, *J* = 5.6 Hz, 2H), 4.96 (dd, *J* = 6.3, 3.7 Hz, 1H), 4.33 (t, *J* = 8.1 Hz, 2H), 3.98 (t, *J* = 6.7 Hz, 2H), 3.64 (d, *J* = 2.7 Hz, 1H), 3.59 (d, *J* = 3.1 Hz, 1H), 3.26–3.19 (m, 2H), 3.11 (d, *J* = 6.8 Hz, 1H), 2.79–2.50 (m, 8H), 2.02–1.65 (m, 4H), 1.62-1.52 (m, 6H), 1.39–1.34 (m, 19H). ^13^C NMR (101 MHz, CDCl_3_) δ 172.8, 155.9, 153.0, 149.0, 141.3, 132.3, 130.9, 126.7, 120.1, 119.0, 114.4, 110.5, 90.6, 85.6, 83.9, 79.6, 66.6, 65.1, 59.6, 52.31, 44.8, 28.4, 25.4, 21.8. HRMS (ESI): calculated for C_33_H_43_N_8_O_7_ [M + H]^+^ 691.3568, found 691.3573.

*Isopropyl-(S)-4-((((3aR,4R,6R,6aR)-6-(6-amino-9H-purin-9-yl)-2,2-dimethyl-tetrahydrofuro[3,4-d][1,3]dioxol-4-yl)methyl)((E)-3-(4-cyanophenyl)allyl)amino)-2-((tert-butoxycarbonyl)amino)butanoate* (**11e**). Following the procedure described for compound **11b**, coupling compound **10** (50 mg, 0.11 mmol) with **5e** (34 mg, 0.13 mmol) afforded compound **11e** as a white powder (47 mg, 62% yield). ^1^H NMR (400 MHz, CDCl_3_) δ 8.16 (s, 1H), 7.92 (s, 1H), 7.45 (d, *J* = 8.3 Hz, 2H), 7.24 (d, *J* = 8.4 Hz, 2H), 6.62 (br s, 2H), 6.32 (br s, 1H), 6.24-6.16 (m, 1H), 6.04–5.99 (m, 1H), 5.91 (d, *J* = 8.1 Hz, 1H), 5.40 (t, *J* = 8.1 Hz, 2H), 4.33-4.25(m, 2H), 3.61 (d, *J* = 2.3 Hz, 1H), 3.56 (d, *J* = 3.6 Hz, 2H), 3.44 (s, 3H), 3.20 (t, *J* = 5.6 Hz, 2H), 3.09 (d, *J* = 4.8 Hz, 1H), 2.78–2.52 (m, 6H), 2.49 (s, 3H), 1.85–1.62 (m, 3H), 1.53 (s, 3H), 1.34 (br s, 20H). ^13^C NMR (101 MHz, CDCl_3_) δ 172.7, 156.1, 153.0, 132.29, 141.3, 132.3, 130.9, 126.6, 119.0, 114.3, 110.4, 90.6, 85.6, 83.9, 83.2, 59.7, 52.3, 51.0, 44.8, 29.2, 28.3, 27.1, 25.4. HRMS (ESI): calculated for C_33_H_43_N_8_O_7_ [M + H]^+^ 691.3568, found 691.3577.

*Benzyl-(S)-4-((((3aR,4R,6R,6aR)-6-(6-amino-9H-purin-9-yl)-2,2-dimethyl-tetrahydrofuro[3,4-d][1,3]dioxol-4-yl)methyl)((E)-3-(4-cyanophenyl)allyl)amino)-2-((tert-butoxycarbonyl)amino)butanoate* (**11f**). Following the procedure described for compound **11b**, coupling compound **5f** (50 mg, 0.11 mmol) with compound **10** (40 mg, 0.13 mmol) afforded compound **11f** as a white powder (54 mg, 66% yield). ^1^H NMR (400 MHz, CDCl_3_) δ 8.18 (s, 1H), 7.91 (s, 1H), 7.48 (d, *J* = 8.4 Hz, 2H), 7.25 (s, 7H), 6.48 (s, 2H), 6.02 (s, 1H), 5.92 (d, *J* = 8.1 Hz, 1H), 5.38 (d, *J* = 6.4 Hz, 2H), 5.19–4.99 (m, 2H), 4.98–4.90 (m, 1H), 4.38–4.31 (br m, 2H), 3.78–3.68 (m, 2H), 3.66 (s, 1H), 3.60 (d, *J* = 3.9 Hz, 3H), 3.48 (s, 5H), 3.23-3.17 (m, 2H), 2.79–2.59 (m, 6H), 1.88–1.82 (m, 2H), 1.74-1.69 (m, 2H), 1.56 (s, 3H), 1.38 (br, 12H). ^13^C NMR (101 MHz, CDCl_3_) δ 172.6, 156.1, 155.8, 153.0, 149.0, 141.2, 135.5, 132.3, 128.2, 126.7, 120.0, 90.6, 85.6, 83.9, 83.3, 66.9, 65.1, 59.8, 56.6, 56.1, 52.4, 44.8, 28.4, 27.2, 25.4. HRMS (ESI): calculated for C_33_H_43_N_8_O_7_ [M + H]^+^ 739.3568, found 739.3571.

*Methyl-(S)-2-amino-4-((((2R,3S,4R,5R)-5-(6-amino-9H-purin-9-yl)-3,4-dihydroxy-tetrahydrofuran-2-yl)methyl)((E)-3-(4-cyanophenyl)allyl)amino)butanoate* (**12b**). To a solution of compound **11b** (30 mg, 0.045 mmol) in 1 mL of CH_2_Cl_2_ was added a mixture of 9 mL TFA and 1 mL H_2_O, and the solution was stirred for 2 h at room temperature. The mixture was concentrated, and the crude product was purified by preparative HPLC affording compound **12b** as a white powder (24mg, 93% yield). ^1^H NMR (400 MHz, CD_3_OD) δ 8.47 (s, 1H), 8.33 (s, 1H), 7.69 (d, *J* = 8.4 Hz, 2H), 7.51 (d, *J* = 8.5 Hz, 2H), 6.84 (d, *J* = 15.8 Hz, 1H), 6.49 (dt, *J* = 15.8, 7.2 Hz, 1H), 6.17 (d, *J* = 3.4 Hz, 1H), 4.70 (dd, *J* = 4.8, 3.4 Hz, 1H), 4.60–4.53 (m, 2H), 4.25 (dd, *J* = 7.4, 5.7 Hz, 1H), 4.14 (d, *J* = 7.3 Hz, 2H), 3.92–3.85 (m, 1H), 3.84 (s, 3H), 3.74–3.66 (m, 1H), 3.64–3.47 (m, 2H), 2.58–2.47 (m, 1H), 2.46–2.34 (m, 1H). ^13^C NMR (101 MHz, CD_3_OD) δ 168.3, 161.84, 161.5, 151.6, 148.2, 139.75, 145.5, 143.0, 139.8, 138.4, 132.3, 127.2, 119.8, 118.191.2, 78.7, 73.5, 72.2, 55.5, 54.9, 52.8, 50.2, 49.8, 24.9. HRMS (ESI): calculated for C_25_H_31_N_8_O_5_ [M + H]^+^ 523.2417, found 523.2422.

*Ethyl-(S)-2-amino-4-((((2R,3S,4R,5R)-5-(6-amino-9H-purin-9-yl)-3,4-dihydroxytetrahydrofuran-2-yl)methyl)((E)-3-(4-cyanophenyl)allyl)amino)butanoate* (**12c**). Following the procedure described for compound **12b**, compound **11c** (30 mg, 0.044 mmol) was deprotected and purified, affording compound **12c** as a white powder (19 mg, 67% yield). ^1^H NMR (400 MHz, CD_3_OD) δ 8.42 (s, 1H), 8.30 (s, 1H), 7.70 (d, *J* = 8.4 Hz, 2H), 7.50 (d, *J* = 8.4 Hz, 2H), 6.84 (br d, *J* = 15.9 Hz, 1H), 6.53–6.42 (m, 1H), 6.15 (d, *J* = 3.4 Hz, 1H), 4.69 (dd, *J* = 5.1, 3.5 Hz, 1H), 4.59–4.48 (m, 2H), 4.33–4.25 (m, 2H), 4.21 (t, *J* = 6.6 Hz, 1H), 4.09 (t, *J* = 7.2 Hz, 2H), 3.80 (dd, *J* = 13.9, 9.8 Hz, 1H), 3.65–3.61 (dd, *J* = 13.2, 5.8 Hz, 1H), 3.56–3.44 (m, 2H), 2.52–2.44 (m, 1H), 2.41–2.30 (m, 1H), 1.30 (t, *J* = 7.1 Hz, 3H). ^13^C NMR (101 MHz, CD_3_OD) δ 167.9, 161.6, 152.5, 148.3, 139.8, 138.0, 132.3, 127.2, 121.0, 119.7, 118.1, 111.7, 91.0, 78.8, 73.4, 72.2, 62.9, 55.5, 55.0, 50.4, 49.9, 25.0. HRMS (ESI): calculated for C_26_H_33_N_8_O_5_ [M + H]^+^ 537.2574, found 537.2579.

*Propyl-(S)-2-amino-4-((((2R,3S,4R,5R)-5-(6-amino-9H-purin-9-yl)-3,4-dihydroxy-tetrahydrofuran-2-yl)methyl)((E)-3-(4-cyanophenyl)allyl)amino)butanoate* (**12d**). Following the procedure described for compound **12b**, compound **11d** (30 mg, 0.043 mmol) was deprotected and purified, affording compound **12d** as a white powder (20 mg, 69% yield). ^1^H NMR (400 MHz, CD_3_OD) δ 8.46 (s, 1H), 8.33 (s, 1H), 7.69 (d, *J* = 8.3 Hz, 2H), 7.51 (d, *J* = 8.3 Hz, 2H), 6.84 (d, *J* = 15.8 Hz, 1H), 655-6.45 (m, 1H), 6.16 (d, *J* = 3.4 Hz, 1H), 4.75–4.67 (m, 1H), 4.59–4.53 (m, 2H), 4.28–4.09 (m, 5H), 3.90–3.77 (m, 1H), 3.69 (d, *J* = 13.4 Hz, 1H), 3.63–3.47 (m, 2H), 2.57–2.33 (m, 2H), 1.73-1.64 (m, 2H), 1.24 (s, 6H), 0.94 (t, *J* = 7.4 Hz, 3H). ^13^C NMR (101 MHz, CD_3_OD) δ 168.0, 152.0, 148.3, 146.1, 142.8, 139.81, 120.6, 118.1, 111.8, 91.1, 78.8, 73.5, 72.2, 68.3, 55.6, 55.0, 29.8, 25.0, 21.4. HRMS (ESI): calculated for C_27_H_35_N_8_O_5_ [M + H]^+^551.2730, found 551.2732.

*Isopropyl-(S)-2-amino-4-((((2R,3S,4R,5R)-5-(6-amino-9H-purin-9-yl)-3,4-dihydroxytetrahydrofuran-2-yl)methyl)((E)-3-(4-cyanophenyl)allyl)amino)butanoate* (**12e**). Following the procedure described for compound **12b**, compound **11e** (30 mg, 0.043 mmol) was deprotected and purified, affording compound **12e** as a white powder (21 mg, 73% yield). ^1^H NMR (400 MHz, CD_3_OD) δ 8.41 (s, 1H), 8.30 (s, 1H), 7.70 (d, *J* = 8.4 Hz, 2H), 7.50 (d, *J* = 8.3 Hz, 2H), 6.84 (br d, *J* = 15.8 Hz, 1H), 6.53–6.42 (m, 1H), 6.14 (d, *J* = 3.4 Hz, 1H), 5.14–5.07 (m, 1H), 4.70 (dd, *J* = 5.1, 3.5 Hz, 1H), 4.59–4.54 (m, 1H), 4.54–4.48 (m, 1H), 4.18 (t, *J* = 6.6 Hz, 1H), 4.07 (d, *J* = 7.1 Hz, 2H), 2.51–2.29 (m, 2H), 1.29 (t, *J* = 6.1 Hz, 6H). ^13^C NMR (101 MHz, CD_3_OD) δ 167.5, 152.7, 139.8, 137.8, 132.3, 127.2, 119.7, 118.1, 111.7, 91.0, 78.9, 73.4, 72.2, 71.4, 55.1, 50.6, 50.0, 25.1, 20.3. HRMS (ESI): calculated for C_27_H_35_N_8_O_5_ [M + H]^+^ 551.2730, found 551.2734.

*Benzyl-(S)-2-amino-4-((((2R,3S,4R,5R)-5-(6-amino-9H-purin-9-yl)-3,4-dihydroxy-tetrahydrofuran-2-yl)methyl)((E)-3-(4-cyanophenyl)allyl)amino)butanoate* (**12f**). Following the procedure described for compound **12b**, compound **11f** (30 mg, 0.041 mmol) was deprotected and purified, affording compound **12f** as a white powder (22 mg, 77% yield). ^1^H NMR (400 MHz, CD_3_OD) δ 8.41 (s, 1H), 8.29 (s, 1H), 7.48 (s, 1H), 7.46 (s, 1H), 7.43–7.36 (m, 5H), 6.79 (br d, *J* = 15.9 Hz, 1H), 6.47–6.37 (m, 1H), 6.13 (d, *J* = 3.4 Hz, 1H), 5.29 (d, *J* = 2.8 Hz, 2H), 4.67 (dd, *J* = 5.1, 3.4 Hz, 1H), 4.57–4.45 (m, 2H), 4.28 (t, *J* = 6.6 Hz, 1H), 4.03 (d, *J* = 7.2 Hz, 2H), 3.76 (dd, *J* = 13.9, 9.9 Hz, 1H), 3.60 (br d, *J* = 12.8 Hz, 1H), 3.53–3.40 (m, 2H), 2.54–2.33 (m, 2H). ^13^C NMR (101 MHz, CD_3_OD) δ 167.9, 152.1, 148.2, 134.7, 132.3, 119.7, 118.1, 111.78, 91.0, 78.8, 73.4, 72.2, 68.2, 55.5, 54.9, 50.4, 49.9, 25.0. HRMS (ESI): calculated for C_31_H_34_N_8_O_5_ [M + H]^+^598.2652, found 598.2656.

*tert-Butyl-(S)-2-(3-(2-acetoxy-4,6-dimethylphenyl)-3-methylbutanamido)-4-((((3aR,4R,6R,6aR)-6-(6-amino-9H-purin-9-yl)-2,2-dimethyltetrahydrofuro[3,4-d][1,3]dioxol-4-yl)methyl)((E)-3-(4-cyanophenyl)allyl)amino)butanoate* (**13a**). Following the procedure described for compound **11b**, coupling compound **10** (95 mg, 0.21 mmol, 1.1 eq) with compound **9a** (80 mg, 0.19 mmol, 1.0 eq) afforded compound **13a** as a yellowish oil (55 mg, 34% yield). ^1^H NMR (400 MHz, CDCl_3_) δ 8.24 (s, 1H), 7.90 (s, 1H), 7.55 (d, *J* = 8.4 Hz, 1H), 7.31 (d, *J* = 8.4 Hz, 2H), 6.79 (d, *J* = 2.1 Hz, 1H), 6.58 (d, *J* = 2.1 Hz, 1H), 6.43–6.19 (m, 3H), 6.14 (s, 2H), 6.04 (d, *J* = 2.0 Hz, 1H), 5.44 (dd, *J* = 6.4, 2.0 Hz, 1H), 4.98 (dd, *J* = 6.4, 3.6 Hz, 1H), 4.38–4.27 (m, 2H), 3.21 (d, *J* = 6.3 Hz, 2H), 2.77–2.66 (m, 2H), 2.57 (q, *J* = 13.5 Hz, 2H), 2.49 (s, 3H), 2.31 (s, 3H), 2.20 (s, 3H), 1.85–1.73 (m, 1H), 1.67–1.56 (m, 11H), 1.52–1.38 (m, 16H), 1.26 (s, 9H). ^13^C NMR (101 MHz, CDCl_3_) δ 171.15, 170.94, 170.76, 155.65, 153.02, 149.87, 149.12, 141.25, 140.14, 138.46, 136.51, 133.68, 132.73, 132.39, 130.90, 126.69, 124.82, 123.39, 119.05, 114.43, 114.13, 110.59, 90.80, 85.71, 83.96, 83.25, 81.71, 56.83, 56.01, 51.56, 50.84, 49.26, 43.53, 39.80, 31.97, 31.81, 31.69, 30.35, 29.74, 29.55, 29.46, 27.96, 27.94, 27.20, 25.53, 25.43, 21.97, 20.27. HRMS (ESI): calculated for C_46_H_59_N_8_O_8_ [M + H]^+^ 851.4450, found 851.4456.

*Methyl-(S)-2-(3-(2-acetoxy-4,6-dimethylphenyl)-3-methylbutanamido)-4-((((3aR,4R,6R,6aR)-6-(6-amino-9H-purin-9-yl)-2,2-dimethyltetrahydrofuro[3,4-d][1,3]dioxol-4-yl)methyl)((E)-3-(4-cyanophenyl)allyl)amino)butanoate* (**13b**). To a solution of compound **11b** (30 mg, 0.045 mmol) in 9 mL dry of CH_2_Cl_2_ was added a 1 mL TFA and the solution was stirred for 1 h at room temperature. The mixture was concentrated. An amount of 5 mL CH_2_Cl_2_ was added to the mixture, followed by adding benzotriazol-1-yloxytris(dimethylamino)phosphonium hexafluorophosphate (BOP, 20 mg), 3-(2-acetoxy-4,6-dimethylphenyl)-3-methylbutanoic acid 7 (TML acid, 0.045 mmol, 12 mg), and 0.5 mL Et_3_N. The reaction mixture was stirred for 2 h at r.t., 10 mL water was added, extracted with CH_2_Cl_2_ (10 mL × 3), then the combined organic phase was dried over Na_2_SO_4_. The solvent was evaporated, and the crude product was purified by column chromatography (100% EtOAc) to give compound **13b** as a white powder (15 mg, 42% yield over 2 steps). ^1^H NMR (400 MHz, CDCl_3_) δ 8.20 (s, 1H), 7.91 (s, 1H), 7.54 (d, *J* = 8.3 Hz, 2H), 7.30 (d, *J* = 8.5 Hz, 2H), 6.79 (d, *J* = 1.5 Hz, 1H), 6.58 (d, *J* = 7.3 Hz, 3H), 6.43–6.32 (m, 2H), 6.27–6.17 (m, 1H), 6.04 (d, *J* = 2.0 Hz, 1H), 5.43 (dd, *J* = 6.4, 2.0 Hz, 1H), 4.97 (dd, *J* = 6.4, 3.4 Hz, 1H), 4.48-4.43 (m, 1H), 4.37-4.32 (m, 1H), 3.62 (s, 3H), 3.19 (d, *J* = 6.2 Hz, 2H), 2.69 (d, *J* = 6.8 Hz, 2H), 2.55 (d, *J* = 4.8 Hz, 2H), 2.48 (s, 3H), 2.41–2.32 (m, 1H), 2.30 (s, 3H), 2.19 (s, 3H), 2.12 (s, 1H), 1.81-1.74 (m, 1H), 1.69–1.55 (m, 9H), 1.38 (s, 3H). ^13^C NMR (101 MHz, CDCl_3_) δ 172.5, 171.1, 155.8, 152.7, 149.9, 141.2, 140.1, 138.5, 136.6, 132.7, 133.6, 132.4, 131.0, 130.9, 126.7, 123.4, 114.4, 110.6, 90.9, 85.6, 83.9, 83.2, 56.4, 55.9, 52.2, 50.9, 49.2, 39.8, 31.7, 29.1, 27.2, 25.5, 25.3, 22.0, 20.2. HRMS (ESI): calculated for C_43_H_53_N_8_O_8_ [M + H]^+^809.3986, found 809.3991.

*Ethyl-(S)-2-(3-(2-acetoxy-4,6-dimethylphenyl)-3-methylbutanamido)-4-((((3aR,4R,6R,6aR)-6-(6-amino-9H-purin-9-yl)-2,2-dimethyltetrahydrofuro[3,4-d][1,3]dioxol-4-yl)methyl)((E)-3-(4-cyanophenyl)allyl)amino)butanoate* (**13c**). Following the procedure described for compound **13b**, compound **11c** (85 mg, 0.126 mmol) was selectively deprotected and coupled with TML acid 7 (40 mg, 0.15 mmol), affording crude **13c** which was used in the next step without further purification.

*Propyl-(S)-2-(3-(2-acetoxy-4,6-dimethylphenyl)-3-methylbutanamido)-4-((((3aR,4R,6R,6aR)-6-(6-amino-9H-purin-9-yl)-2,2-dimethyltetrahydrofuro[3,4-d][1,3]dioxol-4-yl)methyl)((E)-3-(4-cyanophenyl)allyl)amino)butanoate* (**13d**). Following the procedure described for compound **13b**, compound **11d** (31 mg, 0.045 mmol) was selectively deprotected and coupled with TML acid 7 (12 mg, 0.045 mmol), affording crude **13d** which was used in the next step without further purification.

*Isopropyl-(S)-2-(3-(2-acetoxy-4,6-dimethylphenyl)-3-methylbutanamido)-4-((((3aR,4R,6R,6aR)-6-(6-amino-9H-purin-9-yl)-2,2-dimethyltetrahydrofuro[3,4-d][1,3]dioxol-4-yl)methyl)((E)-3-(4-cyanophenyl)allyl)amino)butanoate* (**13e**). Following the procedure described for compound **13b**, compound **11e** (31 mg, 0.045 mmol) was selectively deprotected and coupled with TML acid 7 (15 mg, 0.045 mmol), affording compound **13e** as a white powder (18 mg, 39% yield over 2 steps). ^1^H NMR (400 MHz, CDCl_3_) δ 8.22 (s, 1H), 7.90 (s, 1H), 7.54 (d, *J* = 8.3 Hz, 2H), 7.30 (d, *J* = 6.4 Hz, 2H), 6.79 (d, *J* = 1.6 Hz, 1H), 6.59 (d, *J* = 1.6 Hz, 1H), 6.43–6.32 (m, 4H), 6.23 (dt, *J* = 15.9, 6.4 Hz, 1H), 6.04 (d, *J* = 2.0 Hz, 1H), 5.44 (dd, *J* = 6.4, 2.0 Hz, 1H), 5.01–4.92 (m, 2H), 4.46–4.30 (m, 2H), 3.20 (d, *J* = 6.2 Hz, 2H), 2.70 (d, *J* = 7.9 Hz, 2H), 2.64–2.51 (m, 3H), 2.49 (s, 3H), 2.41–2.36 (m, 2H), 2.30 (s, 3H), 2.20 (s, 3H), 1.83–1.78 (m, 1H), 1.67–1.57 (m, 11H), 1.38 (s, 3H), 1.26 (s, 4H), 1.20 (d, *J* = 6.3 Hz, 3H), 1.15 (d, *J* = 6.2 Hz, 3H). ^13^C NMR (101 MHz, CDCl_3_) δ 171.6, 171.0, 170.8, 155.7, 152.9, 149.9, 141.2, 140.1, 138.6, 132.7, 132.4, 123.42, 120.2, 119.0, 114.4, 109.8, 90.9, 85.7, 84.0, 83.2, 68.9, 56.7, 56.0, 51.1, 50.8, 49.2, 39.8, 31.7, 29.7, 27.2, 25.5, 25.4, 22.0, 21.7, 20.3. HRMS (ESI): calculated for C_45_H_57_N_8_O_8_ [M + H]^+^ 837.4299, found 837.4303.

*Benzyl-(S)-2-(3-(2-acetoxy-4,6-dimethylphenyl)-3-methylbutanamido)-4-((((3aR,4R,6R,6aR)-6-(6-amino-9H-purin-9-yl)-2,2-dimethyltetrahydrofuro[3,4-d][1,3]dioxol-4-yl)methyl)((E)-3-(4-cyanophenyl)allyl)amino)butanoate* (**13f**). Following the procedure described for compound **13b**, compound **11f** (82 mg, 0.13 mmol) was selectively deprotected and coupled with TML acid 7 (34 mg, 0.13 mmol), affording crude **13f** which was used in the next step without further purification.

*(S)-2-(3-(2-acetoxy-4,6-dimethylphenyl)-3-methylbutanamido)-4-((((2R,3S,4R,5R)-5-(6-amino-9H-purin-9-yl)-3,4-dihydroxytetrahydrofuran-2-yl)methyl)((E)-3-(4-cyanophenyl)allyl)amino)butanoic acid* (**14a**). Following the procedure described for compound **12a**, compound **13a** (55 mg, 0.065 mmol) was deprotected and purified, affording compound **14a** as a white powder (28 mg, 54% yield). ^1^H NMR (400 MHz, CD_3_OD) δ 8.47 (s, 1H), 8.35 (s, 1H), 7.72 (d, *J* = 8.1 Hz, 2H), 7.54 (d, *J* = 8.1 Hz, 2H), 6.85–6.75 (m, 2H), 6.61 (d, *J* = 2.1 Hz, 1H), 6.44–6.34 (m, 1H), 6.15 (d, *J* = 3.5 Hz, 1H), 4.54–4.47 (m, 2H), 4.32 (dd, *J* = 8.6, 4.8 Hz, 1H), 3.93 (d, *J* = 7.5 Hz, 2H), 3.84–3.76 (m, 1H), 3.62 (d, *J* = 13.8 Hz, 1H), 3.27–3.16 (m, 2H), 2.76 (d, *J* = 14.9 Hz, 1H), 2.69–2.60 (m, 1H), 2.55 (s, 3H), 2.33 (s, 3H), 2.16 (s, 3H), 2.08–1.96 (m, 1H), 1.60 (s, 3H), 1.56 (s, 3H). ^13^C NMR (101 MHz, CD_3_OD) δ 175.2, 171.7, 162.9, 157.1, 150.4, 147.3, 139.6, 138.7, 138.3, 136.0, 134.4, 133.0, 132.3, 131.9, 127.3, 123.0, 119.8, 118.1, 117.7, 114.8, 112.4, 91.1, 77.0, 73.4, 71.7, 54.9, 50.9, 50.5, 36.9, 31.0, 29.7, 26.3, 20.5, 18.5. HRMS (ESI): calculated for C_39_H_47_N_8_O_8_ [M + H]^+^ 755.3511, found 755.3508.

*Methyl-(S)-2-(3-(2-acetoxy-4,6-dimethylphenyl)-3-methylbutanamido)-4-((((2R,3S,4R,5R)-5-(6-amino-9H-purin-9-yl)-3,4-dihydroxytetrahydrofuran-2-yl)methyl)((E)-3-(4-cyanophenyl)allyl)amino)butanoate* (**14b**). Following the procedure described for compound **12a**, compound **13b** (10 mg, 0.012 mmol) was deprotected and purified, affording compound **14b** as a white powder (5 mg, 48% yield). ^1^H NMR (400 MHz, CD_3_OD) δ 8.48 (s, 1H), 8.36 (s, 1H), 7.70 (d, *J* = 8.3 Hz, 2H), 7.53 (d, *J* = 8.1 Hz, 2H), 6.87–6.77 (m, 2H), 6.61 (d, *J* = 1.5 Hz, 1H), 6.42–6.35 (m, 1H), 6.17 (d, *J* = 3.5 Hz, 1H), 4.69 (t, *J* = 3.8 Hz, 1H), 4.51 (d, *J* = 6.6 Hz, 2H), 4.40 (dd, *J* = 8.7, 5.0 Hz, 1H), 3.95 (br s, 2H), 3.82–3.74 (m, 1H), 3.69 (s, 3H), 3.62 (br d, *J* = 13.6 Hz, 1H), 3.25–3.18 (m, 2H), 2.76 (d, *J* = 14.9 Hz, 1H), 2.64 (br d, *J* = 14.8 Hz, 1H), 2.54 (s, 3H), 2.16 (s, 3H), 2.00 (s, 1H), 1.61 (s, 3H), 1.55 (s, 3H). ^13^C NMR (101 MHz, CD_3_OD) δ 172.9, 170.9, 160.96, 161.3, 161.0, 160.6, 160.2, 151.1, 149.7, 148.1, 139.66, 138.7, 136.0, 133.6, 132.3, 131.9, 127.3, 123.0, 119.9, 118.1, 117.7, 114.8, 111.9, 91.1, 78.5, 73.4, 72.2, 54.3, 51.8, 49.5, 39.1, 31.1, 30.8, 26.1, 24.4, 20.6, 18.9. HRMS (ESI): calculated for C_40_H_49_N_8_O_8_ [M + H]^+^ 769.3673, found 769.3681.

*Ethyl-(S)-2-(3-(2-acetoxy-4,6-dimethylphenyl)-3-methylbutanamido)-4-((((2R,3S,4R,5R)-5-(6-amino-9H-purin-9-yl)-3,4-dihydroxytetrahydrofuran-2-yl)methyl)((E)-3-(4-cyanophenyl)allyl)amino)butanoate* (**14c**). Following the procedure described for compound **12a**, compound **13c** (10 mg, 0.012 mmol) was deprotected and purified, affording compound **14c** as a white powder (6 mg, 56% yield). ^1^H NMR (400 MHz, Methanol-*d*_4_) δ 8.46 (s, 1H), 8.34 (s, 1H), 7.72 (d, *J* = 8.4 Hz, 2H), 7.53 (d, *J* = 8.2 Hz, 2H), 6.86–6.78 (m, 2H), 6.61 (d, *J* = 2.1 Hz, 1H), 6.42–6.31 (m, 1H), 6.16 (d, *J* = 3.6 Hz, 1H), 4.70 (s, 1H), 4.55–4.46 (m, 2H), 4.38 (dd, *J* = 8.8, 5.0 Hz, 1H), 4.18–4.13 (m, 2H), 3.93 (s, 2H), 3.81–3.75 (m, 1H), 3.61 (br d, *J* = 14.9 Hz, 1H), 3.26–3.15 (m, 2H), 2.55 (s, 3H), 2.34 (s, 3H), 2.16 (s, 3H), 1.62 (s, 3H), 1.56 (s, 3H), 1.24 (t, *J* = 7.1 Hz, 3H). ^13^C NMR (101 MHz, Methanol-*d*_4_) δ 172.9, 171.0, 151.6, 149.7, 147.1, 139.6, 138.7, 138.3, 136.0, 133.6, 132.7, 131.9, 128.1, 123.6, 119.8, 118.9, 112.0, 91.0, 78.5, 78.5, 73.4, 72.2, 61.6, 55.5, 50.9, 49.6, 39.1, 29.9, 26.1, 24.4, 20.6, 18.8. HRMS (ESI): calculated for C_41_H_51_N_8_O_8_ [M + H]^+^ 783.3830, found 783.3835.

*Propyl-(S)-2-(3-(2-acetoxy-4,6-dimethylphenyl)-3-methylbutanamido)-4-((((2R,3S,4R,5R)-5-(6-amino-9H-purin-9-yl)-3,4-dihydroxytetrahydrofuran-2-yl)methyl)((E)-3-(4-cyanophenyl)allyl)amino)butanoate* (**14d**). Following the procedure described for compound **12a**, compound **13d** (10 mg, 0.012 mmol) was deprotected and purified, affording compound **14d** as a white powder (4 mg, 42% yield). ^1^H NMR (500 MHz, CD_3_OD) δ 8.45 (s, 1H), 8.34 (s, 1H), 7.70 (d, *J* = 8.3 Hz, 2H), 7.51 (d, *J* = 8.1 Hz, 2H), 6.85–6.78 (m, 2H), 6.60 (d, *J* = 1.8 Hz, 1H), 6.40–6.34 (m, 1H), 6.15 (d, *J* = 3.5 Hz, 1H), 4.68 (t, *J* = 4.0 Hz, 1H), 4.52 –4.47 (m, 2H), 4.37 (dd, *J* = 8.6, 5.1 Hz, 1H), 4.04 (t, *J* = 6.7 Hz, 2H), 3.97–3.89 (m, 2H), 3.79–3.74 (m, 1H), 3.64–3.57 (m, 1H), 3.24–3.17 (m, 2H), 2.79–2.70 (m, 2H), 2.67 (d, *J* = 4.2 Hz, 1H), 2.64 (s, 1H), 2.53 (s, 3H), 2.32 (s, 3H), 2.15 (s, 3H), 2.04–1.94 (m, 1H), 1.67–1.58 (m, 6H), 1.54 (s, 3H), 0.91 (t, *J* = 7.4 Hz, 3H). ^13^C NMR (126 MHz, CD_3_OD) δ 172.9, 171.1, 170.6, 149.8, 139.7, 138.4, 136.1, 133.7, 134.4, 132.0, 127.4, 123.1, 119.9, 118.1, 112.0, 91.1, 78.6, 73.5, 72.3, 67.1, 54.5, 50.9, 49.7, 39.2, 31.1, 30.9, 24.5, 21.6, 20.7, 18.9. HRMS (ESI): calculated for C_42_H_53_N_8_O_8_ [M + H]^+^ 783.3986, found 797.3991.

*Isopropyl-(S)-2-(3-(2-acetoxy-4,6-dimethylphenyl)-3-methylbutanamido)-4-((((2R,3S,4R,5R)-5-(6-amino-9H-purin-9-yl)-3,4-dihydroxytetrahydrofuran-2-yl)methyl)((E)-3-(4-cyanophenyl)allyl)amino)butanoate* (**14e**). Following the procedure described for compound **12a**, compound **13e** (10 mg, 0.012 mmol) was deprotected and purified, affording compound **14e** as a white powder (5.3 mg, 49% yield). ^1^H NMR (400 MHz, CD_3_OD) δ 8.47 (s, 1H), 8.35 (s, 1H), 7.71 (d, *J* = 8.1 Hz, 2H), 7.53 (d, *J* = 8.1 Hz, 2H), 6.86–6.77 (m, 2H), 6.61 (d, *J* = 2.1 Hz, 1H), 6.42–6.35 (m, 1H), 6.17 (d, *J* = 3.5 Hz, 1H), 4.72–4.66 (m, 1H), 4.54–4.49 (m, 2H), 4.33 (dd, *J* = 8.8, 5.0 Hz, 1H), 3.93 (d, *J* = 7.5 Hz, 2H), 3.78 (dd, *J* = 14.0, 9.6 Hz, 1H), 3.62 (d, *J* = 13.8 Hz, 1H), 3.27–3.17 (m, 2H), 2.77 (d, *J* = 14.9 Hz, 1H), 2.64 (br d, *J* = 14.9 Hz, 1H), 2.55 (s, 3H), 2.33 (s, 3H), 2.16 (s, 3H), 2.07–1.93 (m, 1H), 1.61 (s, 3H), 1.56 (s, 3H), 1.22 (dd, *J* = 6.3, 1.8 Hz, 6H). ^13^C NMR (101 MHz, CD_3_OD) δ 175.2, 171.7, 162.9, 157.1, 150.4, 147.3, 139.6, 138.7, 138.3, 136.0, 134.4, 133.0, 132.3, 131.9, 127.3, 123.0, 119.8, 118.1, 117.7, 114.8, 112.4, 91.1, 77.0, 73.4, 71.7, 69.2, 54.9, 50.9, 50.5, 36.9, 31.0, 29.7, 26.3, 24.4, 20.5, 18.5. HRMS (ESI): calculated for C_42_H_53_N_8_O_8_ [M + H]^+^ 783.3986, found 797.3994.

*Benzyl-(S)-2-(3-(2-acetoxy-4,6-dimethylphenyl)-3-methylbutanamido)-4-((((2R,3S,4R,5R)-5-(6-amino-9H-purin-9-yl)-3,4-dihydroxytetrahydrofuran-2-yl)methyl)((E)-3-(4-cyanophenyl)allyl)amino)butanoate* (**14f**). Compound **13f** (13mg, 0.015 mmol) was deprotected and purified, affording compound **14f** as a white powder (5 mg, 39%). ^1^H NMR (500 MHz, CD_3_OD) δ 8.41 (s, 1H), 8.31 (s, 1H), 7.69 (d, *J* = 6.3 Hz, 2H), 7.49 (d, *J* = 8.1 Hz, 2H), 7.33 (d, *J* = 9.1 Hz, 5H), 6.82–6.75 (m, 2H), 6.57 (d, *J* = 2.1 Hz, 1H), 6.36–6.27 (m, 1H), 6.12 (d, *J* = 3.5 Hz, 1H), 5.12 (d, *J* = 2.9 Hz, 2H), 4.65 (t, *J* = 4.2 Hz, 1H), 4.50–4.38 (m, 3H), 3.89 (d, *J* = 11.8 Hz, 2H), 3.76–3.70(m, 1H), 3.56 (br d, *J* = 14.6 Hz, 1H), 3.35 (s, 1H), 3.23–3.11 (m, 2H), 2.28 (s, 3H), 2.12 (s, 3H), 2.01–1.93 (m, 1H), 1.56 (s, 3H), 1.49 (s, 3H). ^13^C NMR (126 MHz, CD3OD) δ 171.1, 149.8, 139.6, 138.4, 136.1, 133.7, 1132.4, 128.3, 128.1, 123.1, 91.1, 72.3, 67.1, 42.4, 31.7, 24.4, 20.6, 18.9. HRMS (ESI): calculated for C_46_H_52_N_8_O_8_ [M + H]^+^ 844.3908, found 844.3911.

### 2.2. Ester Stability Assay

The prodrugs were evaluated for their stability in both PBS buffer at pH 7.4 and Tris buffer at pH 8.4. The compounds were dissolved in DMSO at a concentration of 40 mM and diluted with the respective buffer to a final concentration of 1 mM. Compounds were tested directly (t_0_) and subsequently every 2 h over a time period of 16 h by HPLC. Compounds were eluted from a Dr. Maisch ReproSil-Pur C18 column (4.6 × 250 mm, 10 μm particle size, Dr. Maisch, Ammerbuch-Entringen, Germany) with the following solvent system at a flow rate of 0.5 mL/min: solvent A, 0.1% trifluoroacetic acid in water/acetonitrile (95:5); solvent B, 0.1% trifluoroacetic acid in water/acetonitrile (5:95). Gradient elution was as follows: 100:0 (A/B) to 0:100 (A/B) over 8 min, 0:100 (A/B) for 1 min, then reversion back to 100:0 (A/B) over 1 min, 100:0 (A/B) for 2 min. The formation of the parent compound was evaluated and normalized by measuring the peak area at 214 nm and comparing it to the initial timepoint.

### 2.3. Esterase-Mediated Hydrolysis

The conversion of the prodrugs to the parent compound by esterases was evaluated using pig liver esterase (PLE, 18 U/mg, Sigma-Aldrich, St. Louis, MO, USA) in PBS at pH 7.4. Compounds were dissolved in DMSO at 40 mM, diluted to a final concentration of 2 mM with PBS, and added to an equal volume of a 10 mg/mL solution of PLE in PBS (pH 7.4), resulting in final concentrations of 2.5% DMSO, 1 mM compound, and 5 mg/mL PLE. At different time-points 50 µL aliquots were taken, added to 100 µL acetonitrile to precipitate the proteins, and centrifuged for 5 min at 10,000 rpm. The supernatant was subsequently analyzed by HPLC as described in Section 2.2 above.

### 2.4. Cell Proliferation Assay

#### 2.4.1. Cell Culture

HSC-2 human oral cancer cell line, T24 human bladder cancer cell line, and A549 human lung cancer line were cultured in DMEM/F12 medium and supplemented with 10% fetal bovine serum and 50 μg/mL gentamicin at 37 °C in a humidified 5% CO_2_ incubator. For each compound tested, powder was dissolved in DMSO at 100 mM concentration. This stock solution was then diluted in culture medium to final concentration values ranging between 1 µM and 100 µM. For each sample, DMSO was kept constant at 0.1% final concentration. The day before starting treatment, cells were seeded in 96-well plates, at a density of 2000 cells/well. Cells were allowed to attach overnight and then incubated with compounds at different concentrations, or with DMSO only, for 24, 48, and 72 h. All experiments were performed in triplicate.

#### 2.4.2. MTT Cell Viability Assay

Cell proliferation was determined using a colorimetric assay with 3-(4,5-dimethylthiazol-2-yl)-2,5-diphenyl tetrazolium bromide (MTT). The MTT assay measures the conversion of MTT to insoluble formazan by dehydrogenase enzymes of the intact mitochondria of living cells. Cell proliferation was evaluated by measuring the conversion of the tetrazolium salt MTT to formazan crystals upon treatment with compounds or DMSO only for 24, 48, and 72 h. Briefly, cells were incubated for 2 h at 37 °C with 100 µL fresh culture medium containing 5 µL of MTT reagent (5 mg/mL in PBS). The medium was removed and 200 µL isopropanol were added. The amount of formazan crystals formed correlated directly with the number of viable cells. The reaction product was quantified by measuring the absorbance at 540 nm using a plate reader. Experiments were repeated three times. Results were expressed as a percentage of the control (the control equals 100% and corresponds to the absorbance value of each sample at time zero) and presented as mean values ± standard deviation of three independent experiments performed in triplicate.

### 2.5. 1-Methylnicotinamide (MNA) Quantification in A549 Cells

#### 2.5.1. Cell Culture

Human lung adenocarcinoma line (A549, ATCC, Manassas, VA, USA) was cultured according to the provider’s indications and seeded in 6-well format. After 24 h stabilization, when cells reached ≈ 100% confluence, A549 line was preincubated with normal Hank’s buffer (HBSS), then treated with NNMT peptide inhibitors (1 or 10 µM) and applied for a 1 h incubation in a presence of 100 µM nicotinamide and 10 µM *S*-adenosyl-l-methionine (Sigma Aldrich, St. Louis, MO, USA). After incubation, effluent samples were taken and frozen (−80 °C) for further MNA measurement. Cells also were collected, centrifuged (2 × 500 G/5 min.), and frozen both for the measurement of intracellular MNA and for BCA protein assay.

#### 2.5.2. Quantification of 1-Methylnicotinamide (MNA)

The quantification of 1-methylnicotinamide (MNA), nicotinamide (NA), nicotinic acid (NicA), 1-methyl-2-pyridone-5-carboxamide (Met-2Pyr), and 1-methyl-4-pyridone-5-carboxamide (Met-4Pyr) was performed by applying ultra-high pressure liquid chromatography coupled to tandem mass spectrometry (UPLC-MS) according to the methodology previously described with minor modifications [35]. A UPLC-MS system comprised of a UPLC Ultimate 3000 (Dionex, Thermo Scientific, Waltham, MA, USA) connected to a TSQ Quantum Ultra mass spectrometer (Thermo Scientific, Waltham, MA, USA) equipped with a heated electrospray ionization interface (HESI-II Probe, Waltham, MA, USA) was used. Chromatographic separation of analytes was carried out on an Aquasil C18 analytical column (4.6 mm × 150 mm, 5 mm; Thermo Scientific, Waltham, MA, USA) under isocratic elution using acetonitrile with 0.1% of formic acid (A) and 5 mM ammonium formate in water (B) as mobile phases delivered at the flow rate of 0.8 mL/min (A:B, 80:20, *v*/*v*). The cell pellet was resuspended in 60 µL of deionized water and 50 µL of suspension was transferred to a fresh test tube. An amount of 50 µL of effluent sample was used for the measurement of extracellular MNA. The internal standard (IS) containing MNA-*d*_3_ was added to each sample (5 µL) obtaining the final concentration of 500 ng/mL. After the sample mixing, the proteins were precipitated using 100 μL of acidified acetonitrile (0.1% of formic acid), and samples were mixed (10 min), cooled at 4 °C (15 min), and finally centrifuged (15,000× *g*, 15 min, 4 °C). A clear supernatant was transferred to a chromatographic vial and directly injected (5 µL) into UPLC-MS system. The mass spectrometer was operating in the positive ionization mode using the selected reactions monitoring (SRM) mode to monitor the following ion transitions for analyzed metabolite: m/z 137 to 94 for MNA and 140 to 97 for MNA-*d*_3_. The concentration of MNA was calculated based on the calibration curve plotted for the analyte as the relationship between the peak area ratios of analyte/IS to the nominal concentration of the analyte. The concentration of analytes was normalized to milligram of proteins, which was assessed using a Pierce^TM^ BCA Protein Assay Kit (Thermo Fisher, Waltham, MA, USA) and a Synergy4 multiplate reader (BioTek, Winooski, VT, USA). MNA was obtained from Sigma-Aldrich (St. Louis, MO, USA), and the deuterated internal standard MNA-*d*_3_ was synthesized by Dr. Adamus (Technical University, Lodz, Poland). LC-MS–grade acetonitrile, ammonium formate, and formic acid were purchased from Sigma-Aldrich (St. Louis, MO, USA). Ultrapure water was obtained from a Merck-Millipore system (Direct-Q 3UV, Burlington, MA, USA).

### 2.6. Statistical Analysis

Data were analysed using GraphPad Prism software for Windows (GraphPad Software version 9.2.0, San Diego, CA, USA). Significant differences between groups were determined using the one-way analysis of variance (ANOVA). A *p* value < 0.05 was considered as statistically significant.

## 3. Results and Discussion

### 3.1. Chemical Synthesis

**Scheme 1:**
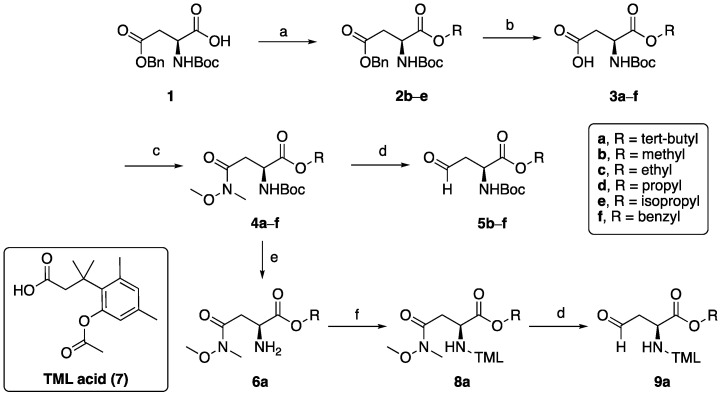
Synthesis of the prodrug forms of amino acid building blocks **5b–f** and **9a**. Reagents and conditions: (**a**) RI, DMF, K_2_CO_3_, rt, overnight (65–79%); (**b**) 10% Pd/C, MeOH, overnight (82–90%); (**c**) CH_3_NHOCH_3_·HCl, BOP, Et_3_N, CH_2_Cl_2_, rt, 2 h (77–83%); (**d**) DIBAL-H (1 M in hexanes), THF, −78 °C, assumed quant; (**e**) HCl (4N in dioxanes), 0 °C to rt, 2.25 h; (**f**) TML acid 7, BOP, Et_3_N, CH_2_Cl_2_, rt, overnight, 88% over two steps.

**Scheme 2:**
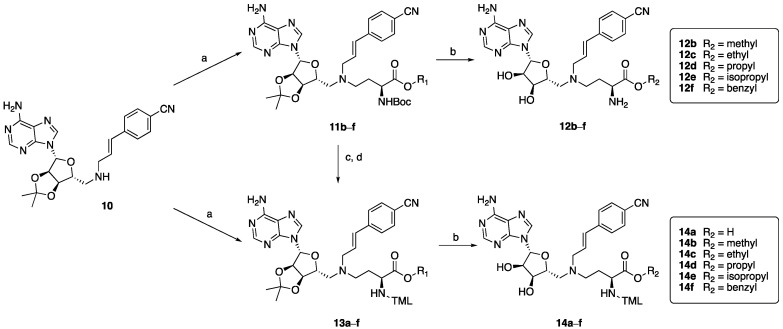
Synthesis of prodrugs **12b**–**f** and **14a**–**f**. Reagents and conditions: (**a**) aldehydes **5b**–**f** or **9a**, NaBH(OAc)_3_, AcOH, DCE, rt, overnight (34–73%); (**b**) TFA, CH_2_Cl_2_, H_2_O, rt, 2 h (70–93%); (**c**) TFA, CH_2_Cl_2_, rt, 2 h; (**d**) TML acid 7, BOP, Et_3_N, CH2Cl_2_, rt, 2 h (77–83%).

The prodrug analogues of GYZ-319 were prepared following the routes depicted in Scheme 1 and Scheme 2. This synthetic approach was based on the one previously developed during our structure-activity relationship studies with bisubstrate NNMT inhibitors leading to the discovery of GYZ-319 [24], allowing for the convenient modification of different parts of the molecule. The ester building blocks (Scheme 1) were synthesized starting from the Boc-Asp(Bn)-OH **1**, which is esterified with the appropriate iodides in the presence of potassium carbonate to produce compounds **2b**–**e**, followed by the deprotection of the benzyl protecting group to obtain compounds **3b**–**e**. Compounds **3a** and **3f** were obtained from commercial sources. Free carboxylic acids **3a**–**f** were then converted into Weinreb amides **4a**–**f** using BOP-coupling conditions and subsequently reduced to the corresponding aldehyde (**5b**–**f**) with DIBAL-H. Compound **4a** followed a different route to produce TML-prodrug **8a** which contains the free carboxylic acid. In order to do so, the Boc group was selectively deprotected using HCl in dioxane to produce free amine **6a**. The free amine was then coupled to the trimethyl-lock acid **7** [36] with BOP and triethylamine to yield Weinreb amide intermediate **8a** followed by DIBAL reduction to form aldehyde **9a**. The aldehydes were subsequently coupled to intermediate **10** via reductive amination conditions, forming intermediates **11b**–**f**, which were then deprotected to form ester prodrugs **12b**–**f** (Scheme 2). Compounds **11b**–**f** can alternatively be selectively Boc-deprotected using TFA/DCM to form intermediates **13b**–**f** and subsequently coupled to trimethyl-lock acid **7** with BOP and triethylamine. Compound **13a** was synthesized through coupling of aldehyde **9a** with compound **10**. The intermediates were then deprotected under acidic conditions to yield ester-TML dual prodrugs **14a**–**f**.

### 3.2. Buffer Stability

All prodrugs prepared were first tested for their inhibitory activity against NNMT using a biochemical enzymatic activity assay. Somewhat surprisingly, several prodrugs showed significant inhibition when tested at fixed concentrations of 5 and 25 µM. In order to evaluate the validity of these results, the prodrugs were subsequently evaluated for their hydrolytic stability in both PBS buffer at pH 7.4 and Tris buffer at pH 8.4. Both these buffers and pHs have been used in the different biochemical and cellular assays described in this report. Compounds were dissolved in DMSO at a concentration of 40 mM and diluted with the respective buffer to a final concentration of 1 mM. Compound stability was then assessed by RP-HPLC directly (t_0_) and subsequently every 2 h over a time period of 16 h. The results presented in Table 1 show that over time, a significant amount of hydrolysis occurs for most of the prodrugs containing only the ester modification. Only the isopropyl ester was found to be stable under these conditions. Interestingly, the stability of the prodrugs increased significantly in the presence of the trimethyl-lock (TML) moiety at the amine position. Even for the rather labile methyl ester **12b**, the TML group in **14b** results in a decrease in hydrolysis of the methyl ester. Benzyl ester **8f** was found to be the least stable, and due to its poor aqueous solubility, the benzyl ester-TML dual prodrug **14f** was not evaluated further. The most stable prodrugs were found to be the isopropyl ester (compound **12e**), the trimethyl-lock (compound **14a**), and the isopropyl-trimethyl-lock dual prodrug (compound **14e**). However, as compound **14a** was found not to improve the cellular activity of the parent compound (see Figure S1 in the Supplementary Information), this compound was not evaluated further.

### 3.3. Esterase-Mediated Hydrolysis

**Figure 2:**
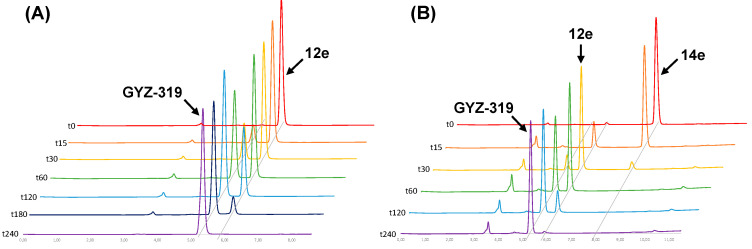
Esterase-mediated hydrolysis bisubstrate inhibitor prodrugs. (**A**) Hydrolysis of isopropyl ester prodrug **12e** and (**B**) hydrolysis of isopropyl-TML dual prodrug **14e**. The data show clean conversion of the prodrugs to the parent compound **17u**. For the dual prodrug **14e**, conversion of the TML happens first followed by the hydrolysis of the isopropyl ester moiety.

The next step was to establish whether compounds **12e** and **14e** are converted to the parent compound in the presence of an esterase. Using commercially available pig liver esterase (PLE), both compounds were found to be readily converted to the parent GYZ-319 as measured by RP-HPLC (Figure 2). Within 4 h, the prodrugs were fully converted to the parent compound, while no hydrolysis was observed in the absence of PLE.

Of note is the sequential conversion of dual prodrug **14e** in which the TML is hydrolyzed first followed by the isopropyl ester (Figure 2B). No trace of compound **14a** could be observed in which the ester is cleaved and the TML is still in place. This finding suggests that the TML group hinders the esterase-mediated hydrolysis of the isopropyl ester moiety and only after deacetylation of the TML moiety followed by its spontaneous loss, can the ester moiety be cleaved by the esterase.

### 3.4. Cellular Assays

**Figure 3:**
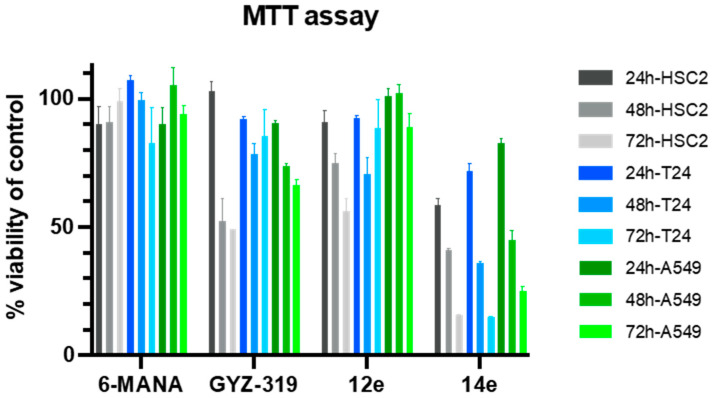
Results of the 3-(4,5-dimethylthiazol-2-yl)-2,5-diphenyl tetrazolium bromide (MTT) cell viability assay on HSC-2 human oral cancer (grey), T24 human bladder cancer (blue), and A549 human lung cancer cells (green) with compounds 6-methylaminonicotinamide (6-MANA), parent compound GYZ-319, and prodrugs **12e** and **14e**. Compounds were incubated for 24, 48, and 72 h at 1, 10, and 100 µM concentrations. As no effects were observed at 1 and 10 µM, only results at 100 µM are depicted. Results are presented as mean values with standard deviation from three independent experiments performed in triplicate.

With the suitability of prodrugs **12e** and **14e** established, the compounds were next tested using an MTT assay to evaluate their effect on cell viability in three different cancer cell lines: HSC-2 (oral cancer), T24 (bladder cancer), and A549 (lung cancer) (Figure 3). The results of these assays revealed that neither the reference NNMT inhibitor 6-methylamino-nicotinamide (6-MANA), nor prodrugs **12e** or **14e** or the parent GYZ-319, showed any appreciable effect on cell viability when tested at 1 and 10 µM concentrations. At the highest concentration tested of 100 µM, 6-MANA again showed no effect while the isopropyl ester prodrug **12e** showed an effect comparable to GYZ-319. By comparison, administration of compound **14e** at 100 µM did cause a time dependent reduction in cell viability for all three cell lines tested. These findings show that both the parent compound and the corresponding prodrug inhibitors have little effect on cell viability unless tested at very high concentrations, more than 10,000 times higher than the activity of the parent compound in biochemical assays (IC_50_ = 3.7 nM). One explanation for this finding may be that the prodrugs are still not effectively entering the cells. An alternative explanation, however, could be that the compounds do in fact enter the cells but that subsequent NNMT inhibition is simply not toxic to the cells. If this is the case, the impact on cell viability observed when applying the compounds at the highest concentration tested (100 µM) could instead by ascribed to a non-specific toxic effect.

**Figure 4:**
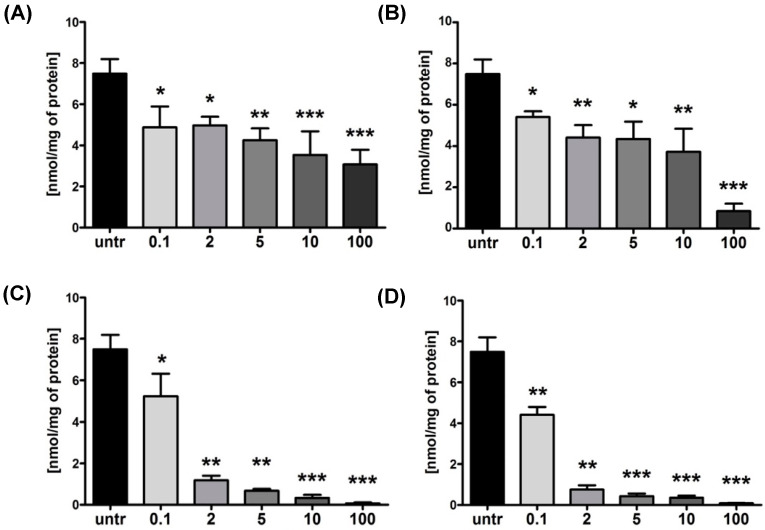
Concentration of 1-methyl nicotinamide (MNA) in A549 lung carcinoma cells after 24-h treatment with 0.1–100 µM of compounds: (**A**) 6-MANA; (**B**) GYZ-319; (**C**) **12e**; and (**D**) **14e**. *n* = 6, * *p* ≤ 0.05, ** *p* ≤ 0.01, *** *p* ≤ 0.001 compared to untreated control (untr).

To more directly investigate the cellular activity of GYZ-391 and the corresponding prodrugs, we next turned to a recently developed assay that allows for the detection and quantification of NNMT activity in live cells [7]. Using this assay, the levels of MNA produced by NNMT in an A549 lung carcinoma cell line can be quantified by means of a sensitive LC-MS method. Using this approach, we evaluated the effect of the reference NNMT inhibitor 6-MANA, parent compound GYZ-319, and prodrugs **12e** or **14e** on cellular MNA production. As illustrated in Figure 4, high concentrations of both 6-MANA and GYZ-319 are required to substantially decrease the levels of MNA in A549 lung cancer cells. In contrast, when the isopropyl ester prodrug **12e** was tested, a clear and significant decrease in MNA levels was observed. This effect is even further enhanced by the introduction of the TML moiety as present in prodrug **14e**.

These findings indicate that the employed prodrug strategy is able to convert a potent, but non-permeable, inhibitor into a compound with cellular activity. Notably, our findings also seem to suggest that the capacity for a small molecule to inhibit NNMT in cells does not *per se* lead to an impact on cell viability. This is in keeping with recent reports showing that the addition of NNMT inhibitors to cancer associated fibroblasts do not kill the cells but rather cause a reversion of cell morphology to one that more closely resembles normal fibroblasts [17].

## 4. Conclusions

In this report, we describe a prodrug approach to translate the biochemical activity of the potent bisubstrate NNMT inhibitor GYZ-319 into cellular activity. The prodrug strategy focused specifically on masking the amino acid functionality of GYZ-319. The carboxylic acid was masked with an ester using a variety of alkyl and benzyl groups and the amine was masked with a trimethyl-lock group, both of which can be released by esterase activity. The different combinations of esterase-cleavable motifs led to the selection of the isopropyl ester **12e** and the isopropyl ester/TML dual prodrug **14e** as the compounds with the most promising profile in terms of stability and cellular activity. When tested against different cancer cell lines, the prodrugs were found to have little impact on cell viability. However, when evaluated in an assay allowing for the direct quantification of cellular MNA production, a clear dose-dependent effect was observed for the prodrugs. Notably, the prodrugs exhibit a significant enhancement of cellular activity compared to the parent compound. The data presented here point to the potential for using such prodrug strategies for the delivery of polar NNMT inhibitors into cells. On-going efforts are focused on further establishing the potential of such NNMT inhibitor prodrugs in a range of cellular and in vivo assays relevant to both oncology and metabolic disease.

## Figures and Tables

**Table 1:**
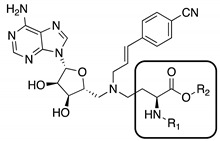
Stability data of the prodrugs in Tris buffer at pH 8.4 and in PBS buffer at pH 7.4. The values indicate the percentage of compound peak area present after 16 h of incubation at room temperature.

Compound	Ester R_2_ (R_1_ = H)	Tris	PBS	Compound	Ester R_2_ (R_1_ = TML)	Tris	PBS
GYZ-319	H	n.a.	n.a.	**14a**	H	93.97	99.10
**12b**	Methyl	22.59	36.97	**14b**	Methyl	73.88	88.08
**12c**	Ethyl	54.84	68.36	**14c**	Ethyl	87.00	90.44
**12d**	Propyl	61.32	71.96	**14d**	Propyl	87.52	90.30
**12e**	Isopropyl	93.70	98.59	**14e**	Isopropyl	87.69	91.13
**12f**	Benzyl	21.60	33.43	**14f**	Benzyl	n.d.	n.d.

n.a. not applicable; n.d. not determined.

## Data Availability

Full materials, synthesis, and purification methods and characterization data for all compounds synthesized are available online and mentioned under Supplementary Materials (above).

## Supplementary Materials

The following are available online at https://www.mdpi.com/article/10.3390/biom11091357/s1, Figure S1: cellular MNA data in HMEc-1 cells treated with 10 µM reference inhibitors or prodrugs; ^1^H and ^13^C NMR spectra of compounds **3e**, **4c**–**e**, **8a**, **11b**–**f**, **12b**–**f**, **13a**–**c**, **13e**, **14a**–**f**.
