# Supplementary material for: Esterase-Sensitive Prodrugs of a Potent Bisubstrate Inhibitor of Nicotinamide N-Methyltransferase (NNMT) Display Cellular Activity
Source: Biomolecules. 2021 Sep 14;11(9):1357. doi: 10.3390/biom11091357 (PMC8466754; doi:10.3390/biom11091357)

Supplementary Information for:

Esterase-Sensitive Prodrugs of a Potent Bisubstrate Inhibitor of Nicotinamide *N*-Methyltransferase (NNMT) Display Cellular Activity

Matthijs J. van Haren[1,†,*], Yongzhi Gao[1,†], Ned Buijs[1], Roberto Campagna[2,3], Davide Sartini[2], Monica Emanuelli[2], Lukasz Mateuszuk[3], Agnieszka Kij[3], Stefan Chlopicki[3,4], Pol Escudé Martinez de Castilla[5], Raymond Schiffelers[5], Nathaniel I. Martin[1,*]

1 Biological Chemistry Group, Institute of Biology Leiden, Leiden University, Sylviusweg 72, 2333 BE Leiden, The Netherlands.
2 Department of Clinical Sciences, Universitá Politecnica delle Marche, Via Ranieri 65, 60131, Ancona, Italy.
3 Jagiellonian University, Jagiellonian Centre for Experimental Therapeutics (JCET), Bobrzynskiego 14, 30-348 Krakow, Poland.
4 Jagielonian University Medical College, Faculty of Medicine, Chair of Pharmacology, Grzegorzecka 16, 31-531 Krakow, Poland
5 Department of Clinical Chemistry and Haematology, Universitair Medisch Centrum Utrecht, Heidelberglaan 100, 3584 CX Utrecht, The Netherlands.
* Correspondence: m.j.van.haren@biology.leidenuniv.nl, n.i.martin@biology.leidenuniv.nl
† These authors contributed equally to this work.

Table of contents

Cellular MNA data S2

NMR spectra S3

Cellular MNA data

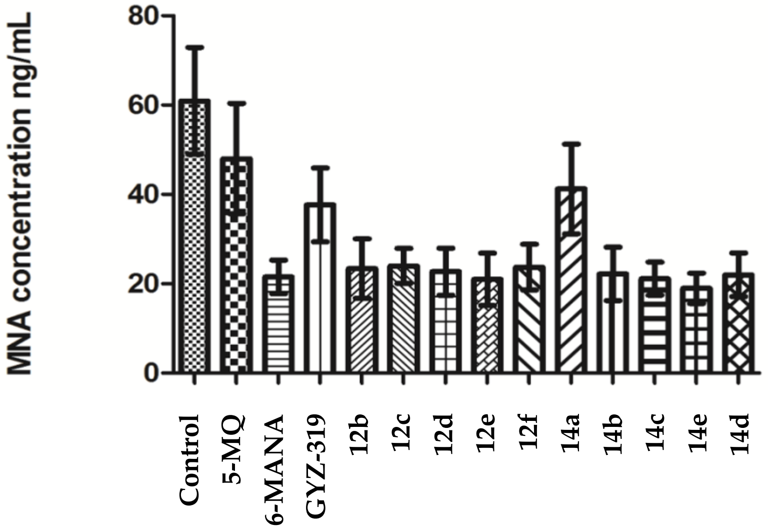

Figure S1. 1-methylnicotinamide (MNA) concentrations in endothelial HMEC-1 cells after 24-hour incubation with 10 μM of reference compounds 5-amino-methylquinolinium (5-MQ), 6-methylamino-nicotinamide (6-MANA), parent compound **GYZ-319** or prodrug compounds **12b**-**f** and **14a**-**e**.

NMR spectra

Compound **3e** ^{1}H NMR (400 MHz, $CDCl_3$)

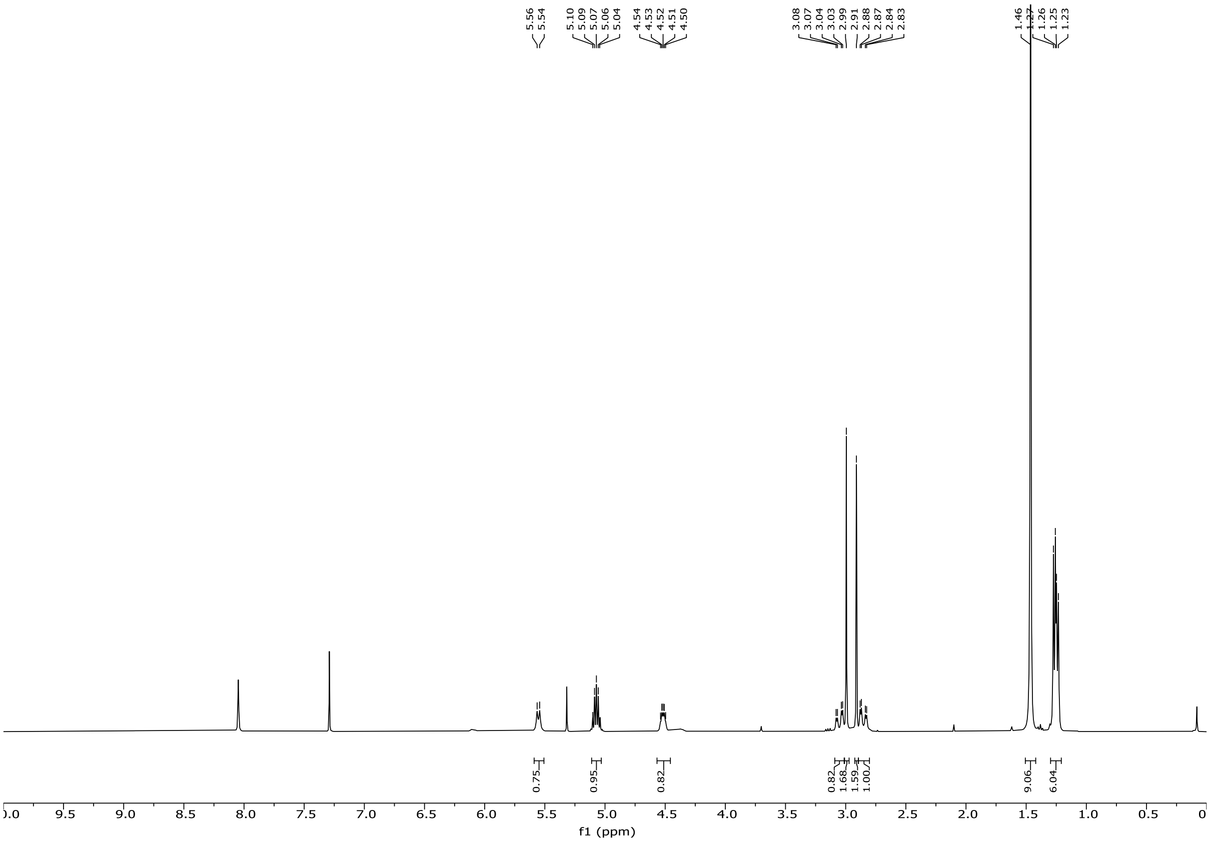

^{13}C NMR (101 MHz, $CDCl_3$)

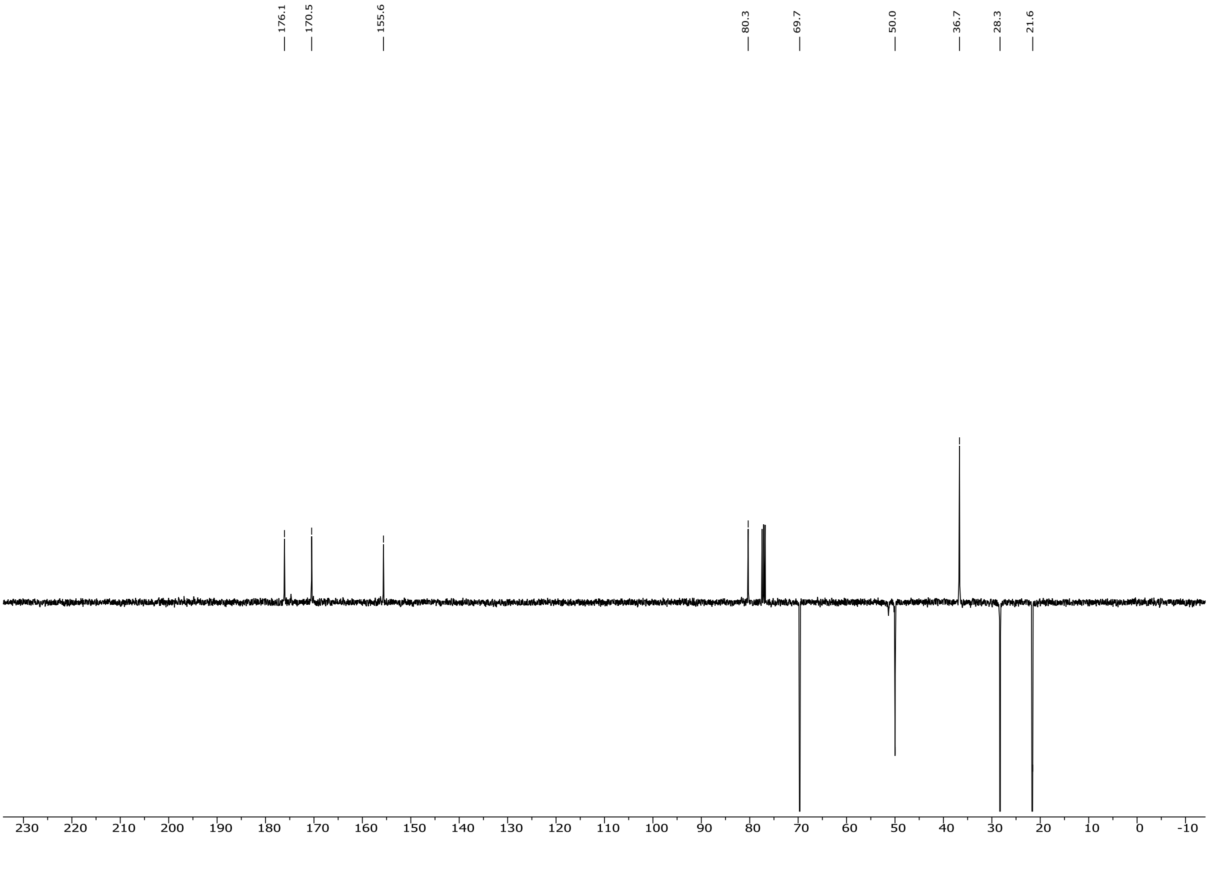

Compound **4d** 1H NMR (400 MHz, $CDCl_3$)

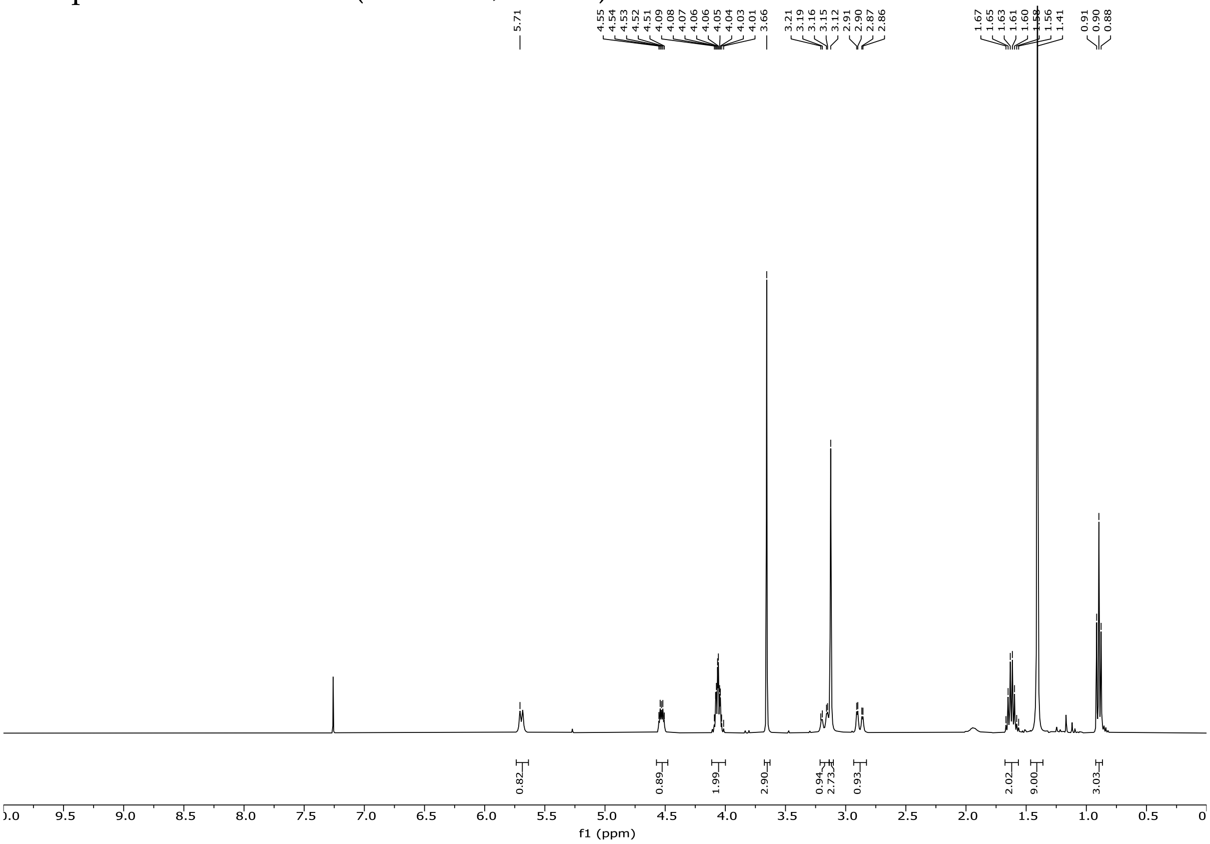

^{13}C NMR (101 MHz, $CDCl_3$)

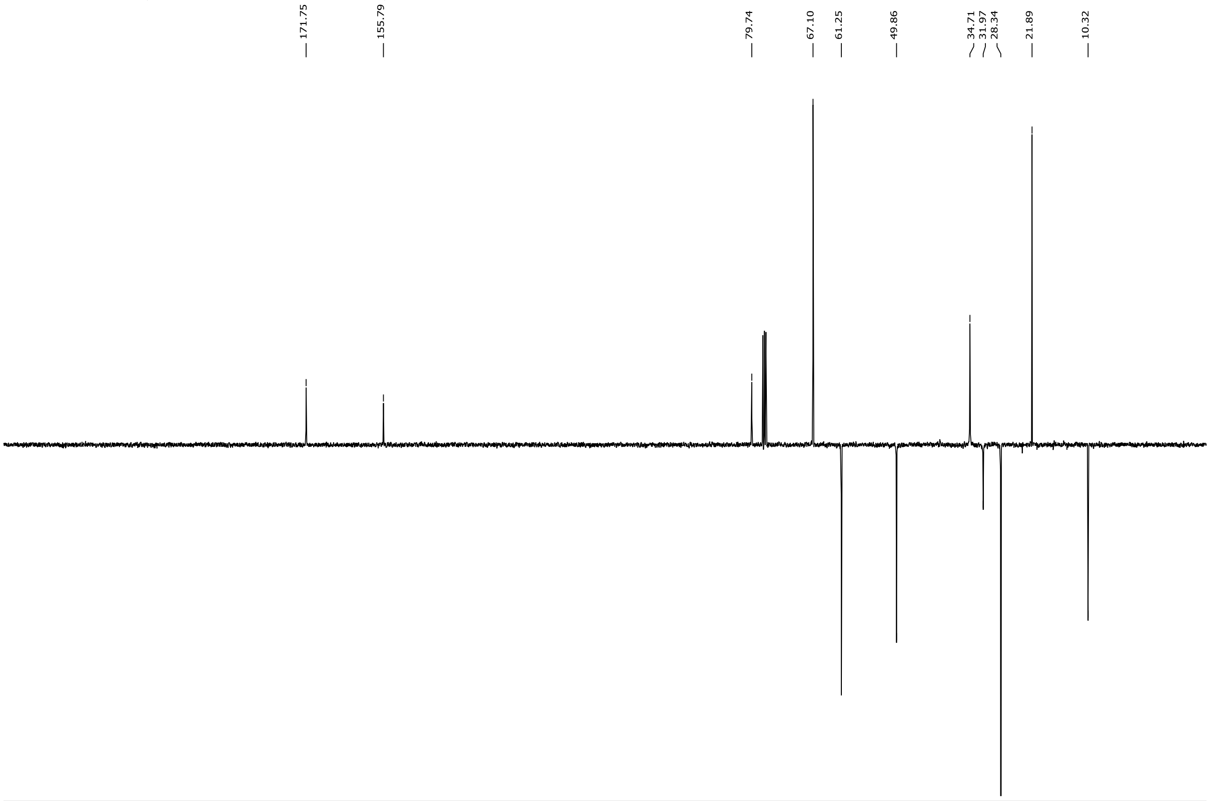

Compound **4e** ^{1}H NMR (400 MHz, $CDCl_3$)

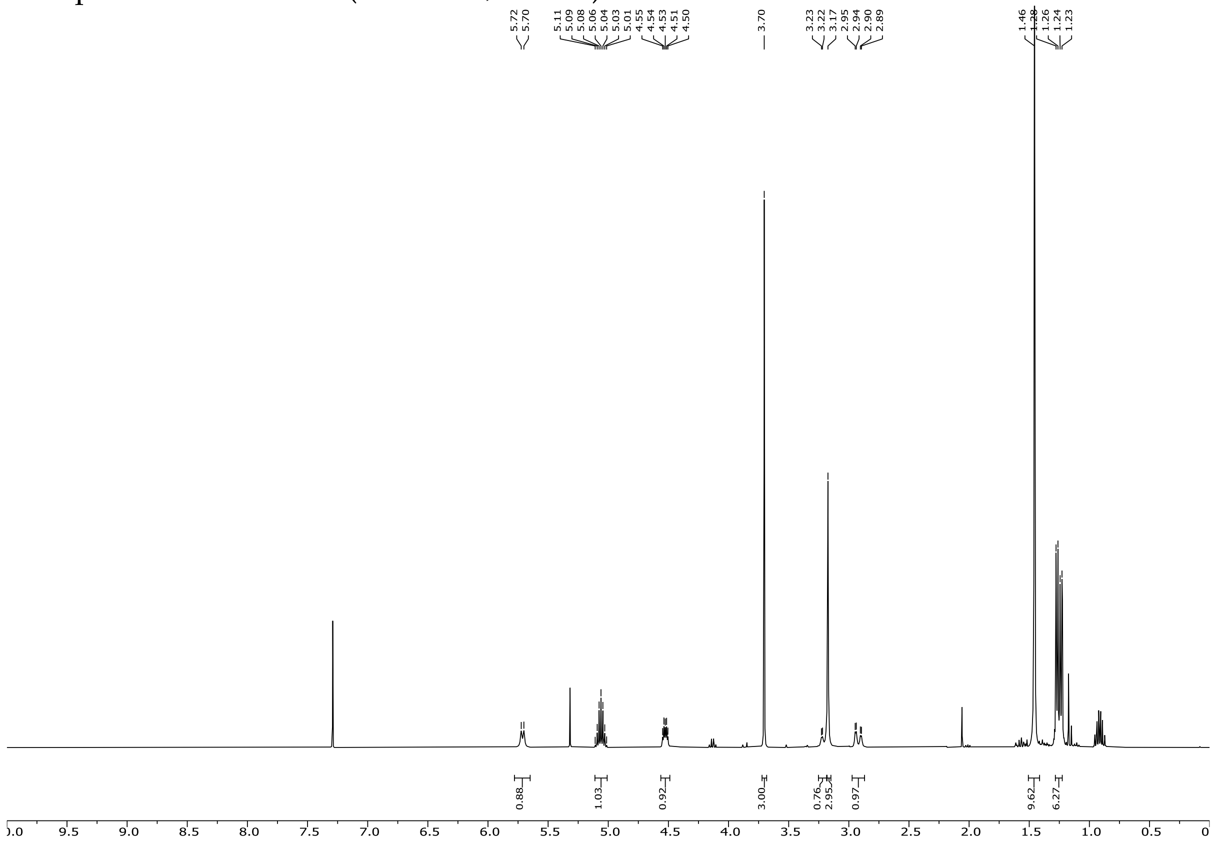

^{13}C NMR (101 MHz, $CDCl_3$)

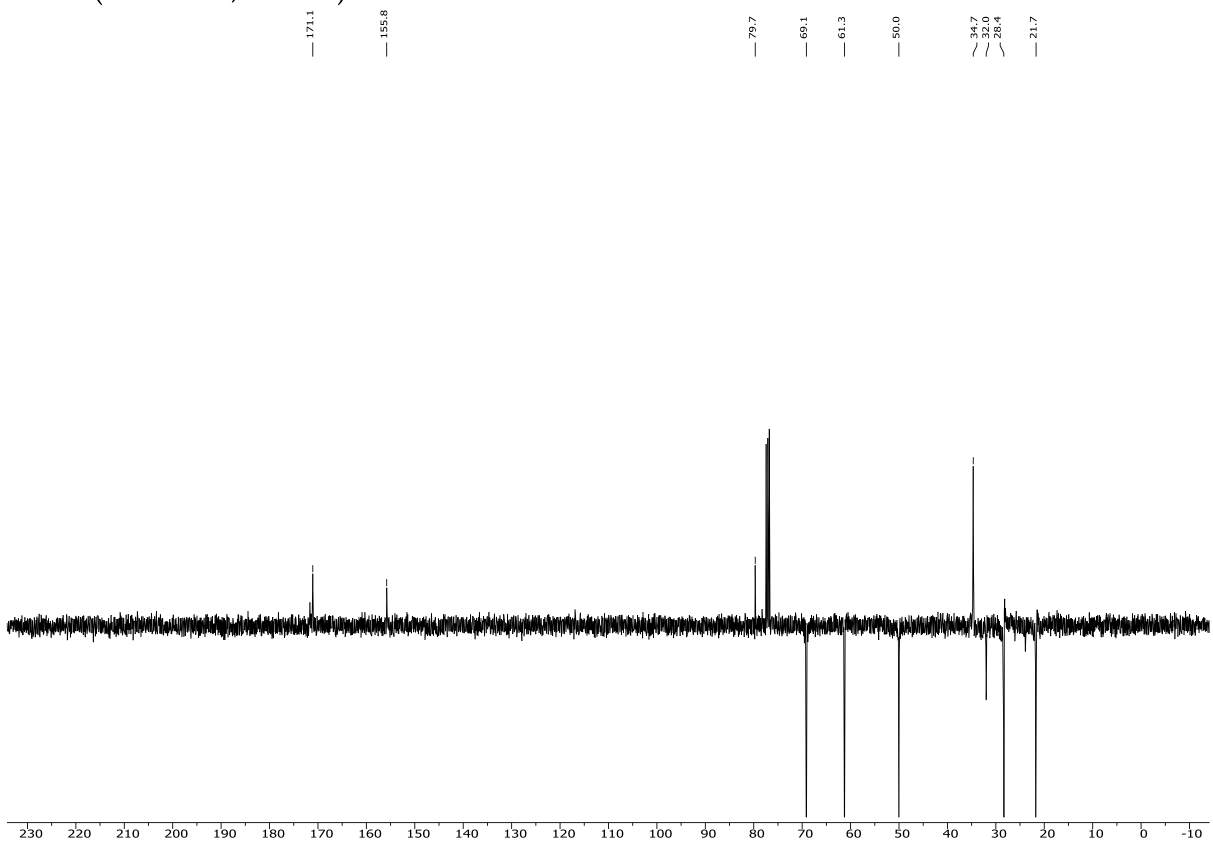

Compound **8a** ^{1}H NMR (300 MHz, $CDCl_3$)

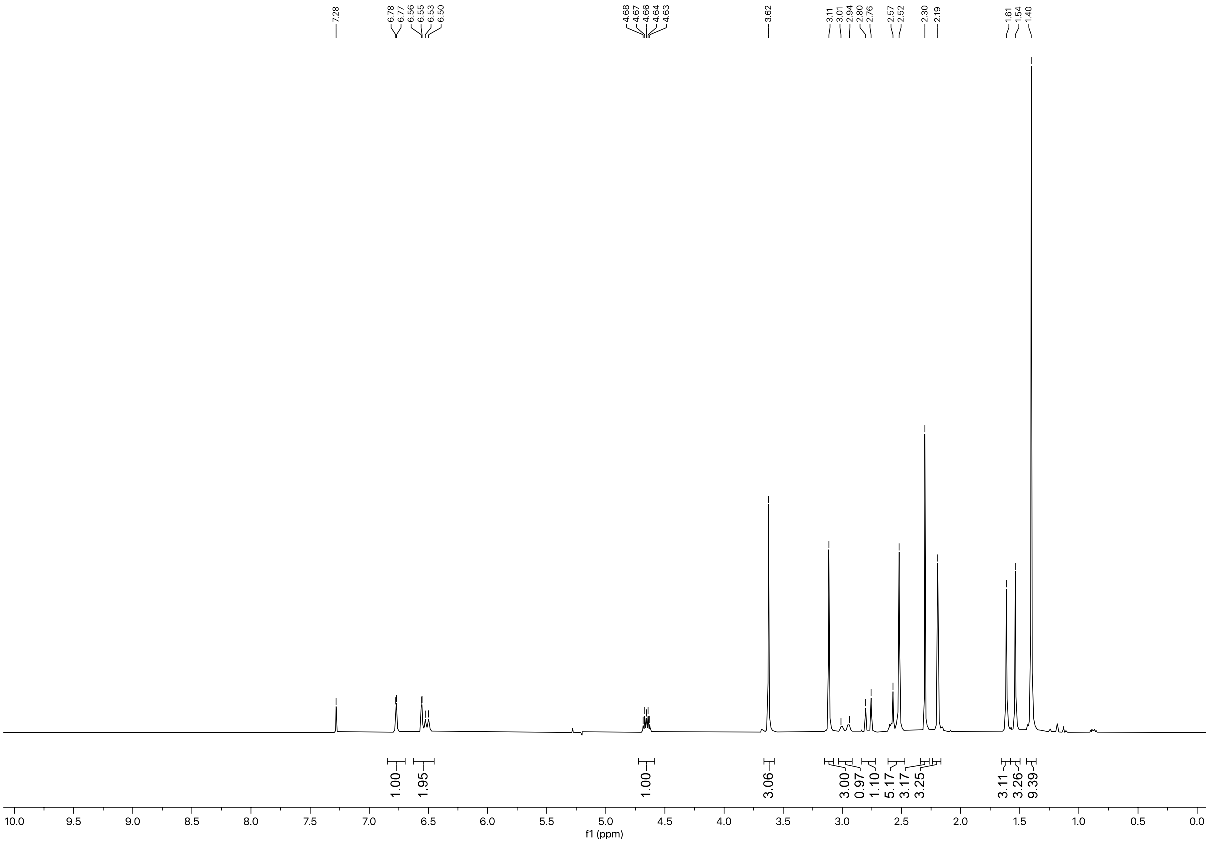

^{13}C NMR (75 MHz, $CDCl_3$)

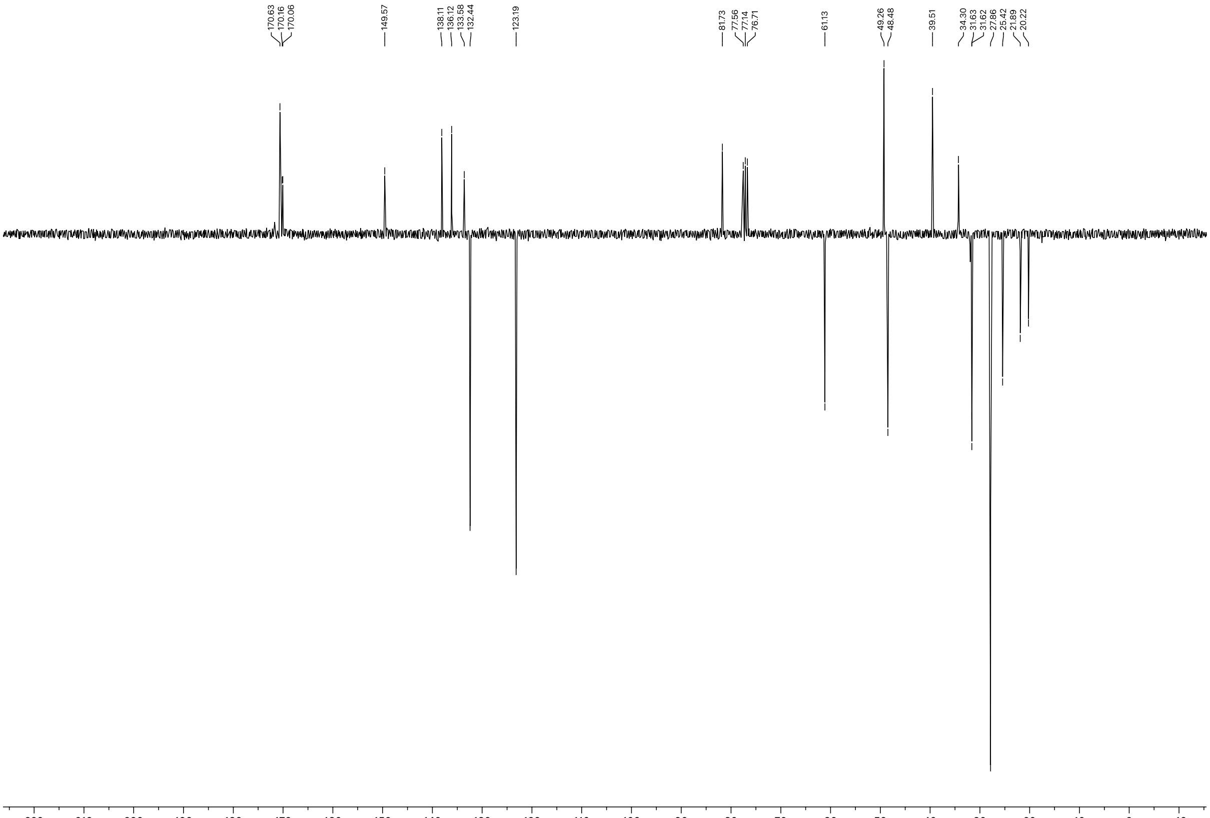

Compound **11b** ^{1}H NMR (400 MHz, $CDCl_3$)

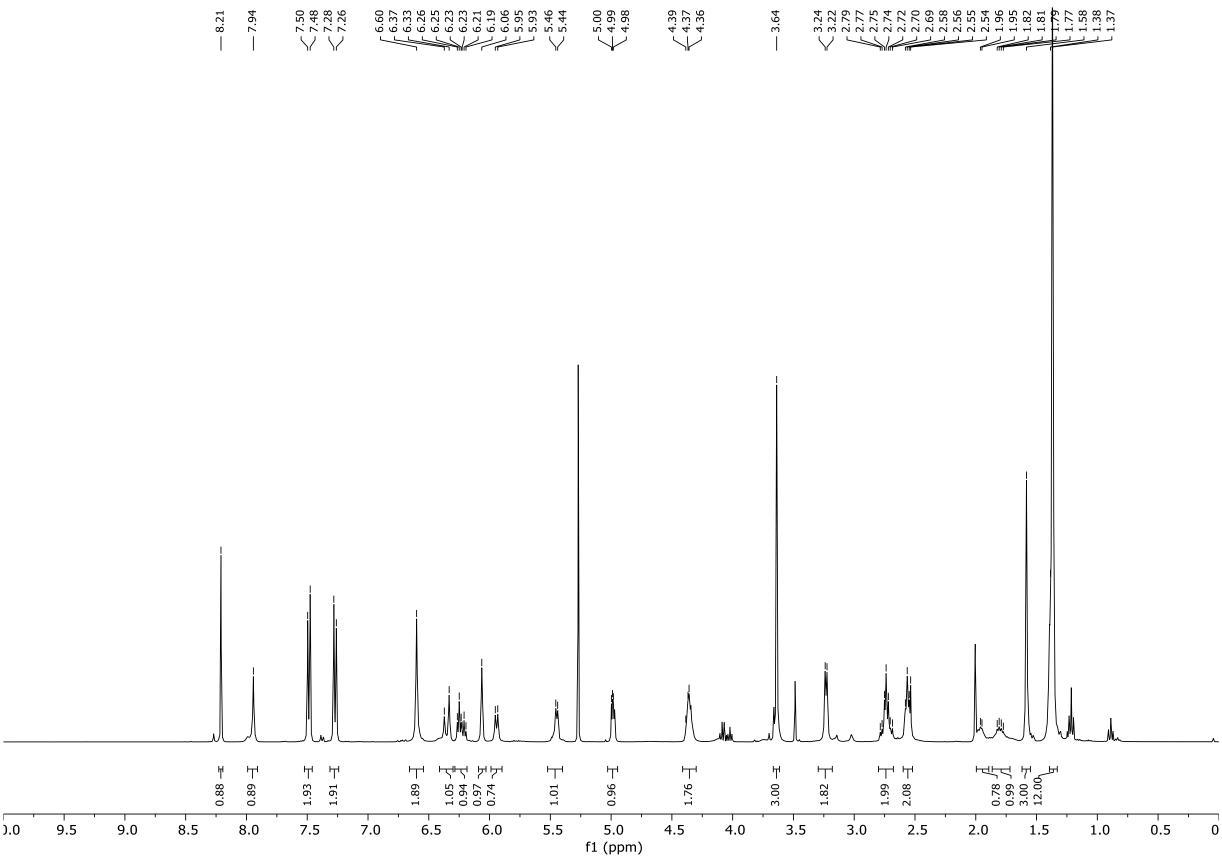

^{13}C NMR $CDCl_3$ (101 MHz, $CDCl_3$)

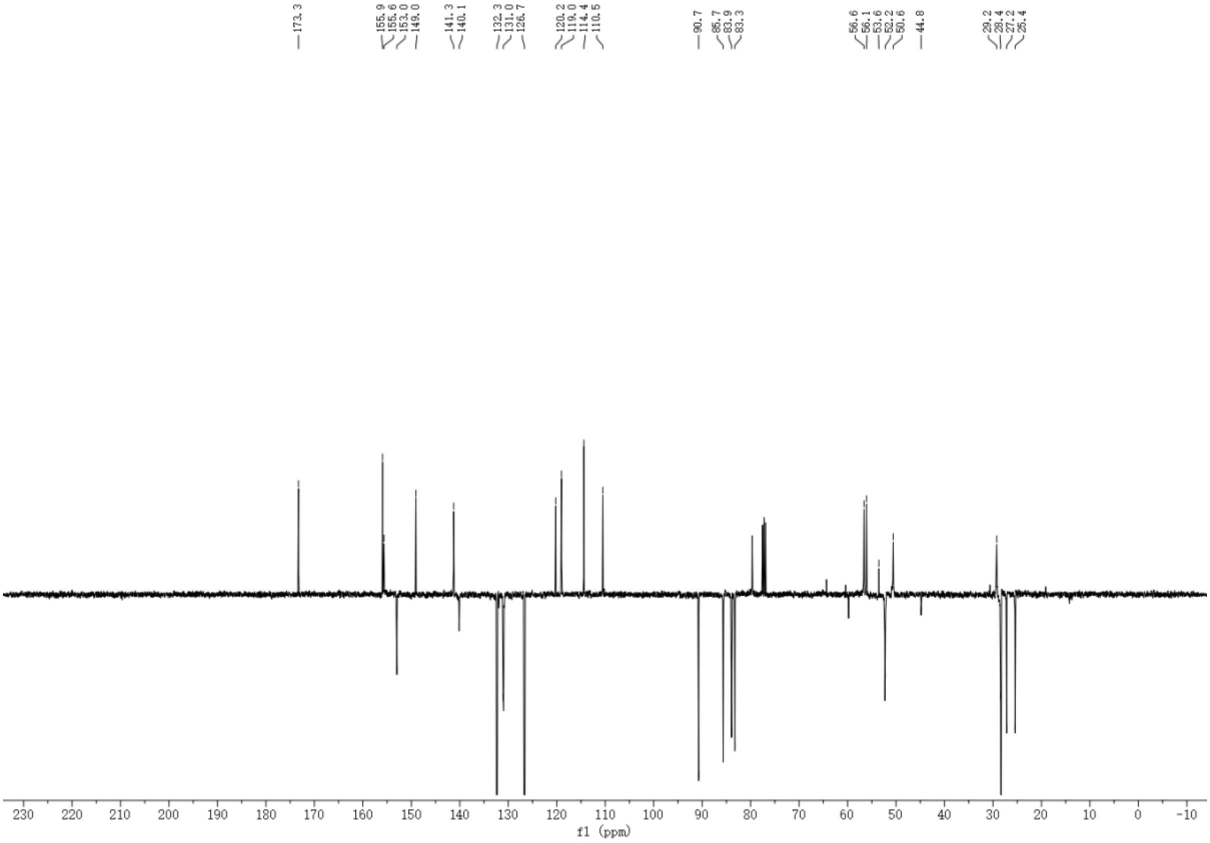

Compound **11c** ^{1}H NMR (400 MHz, $CDCl_3$)

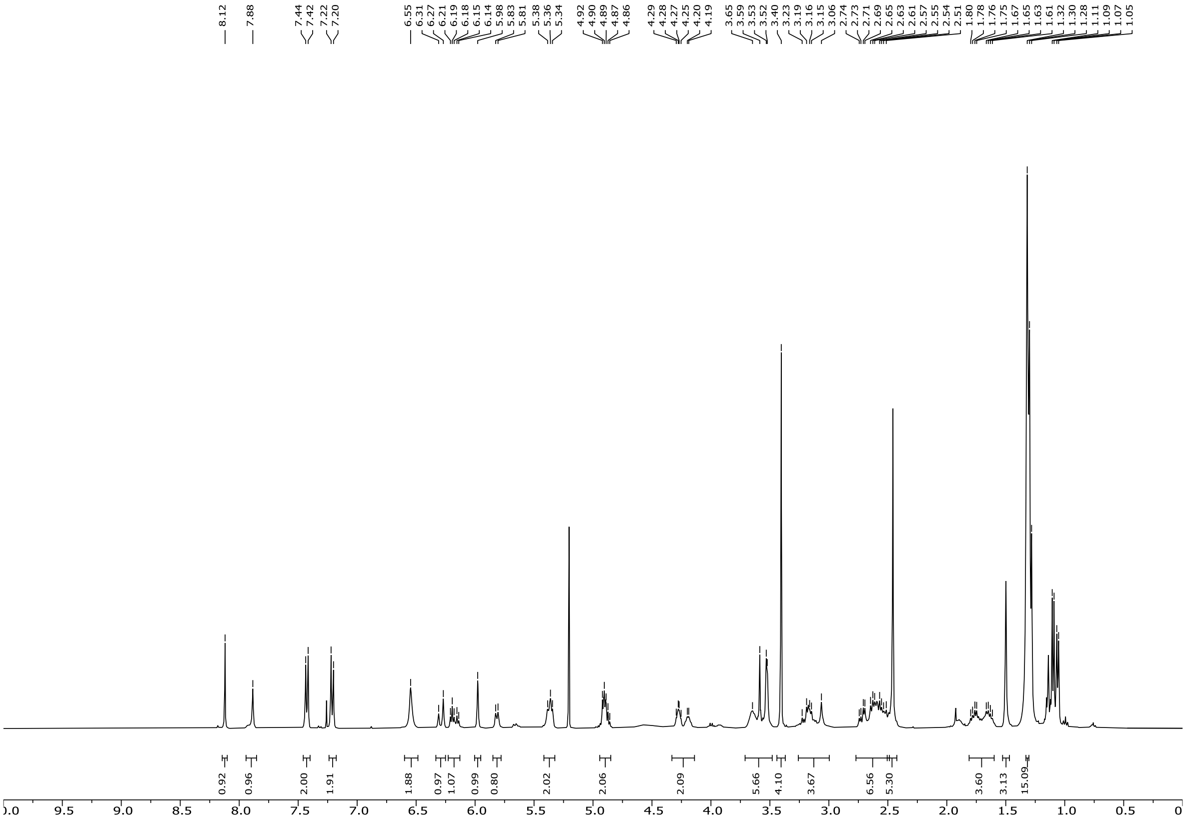

^{13}C NMR (101 MHz, $CDCl_3$)

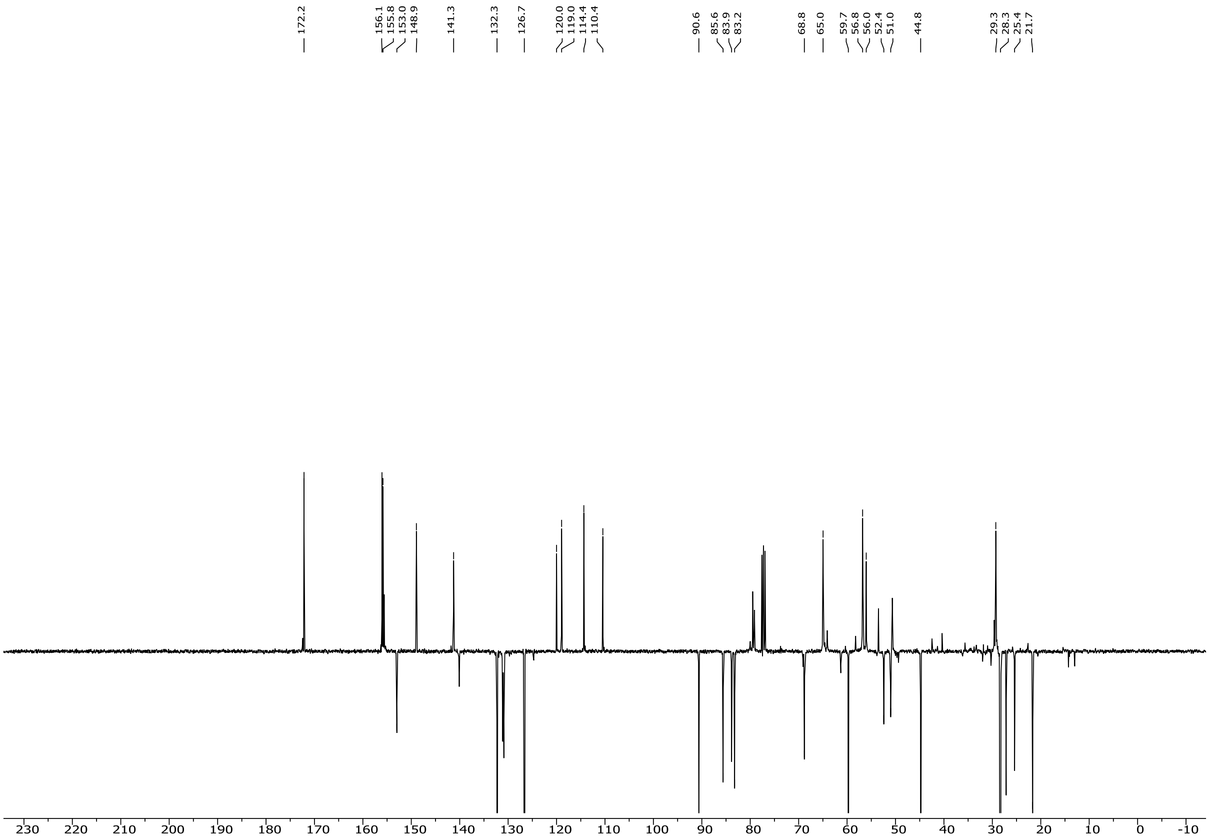

Compound **11d** ^{1}H NMR (400 MHz, $CDCl_3$)

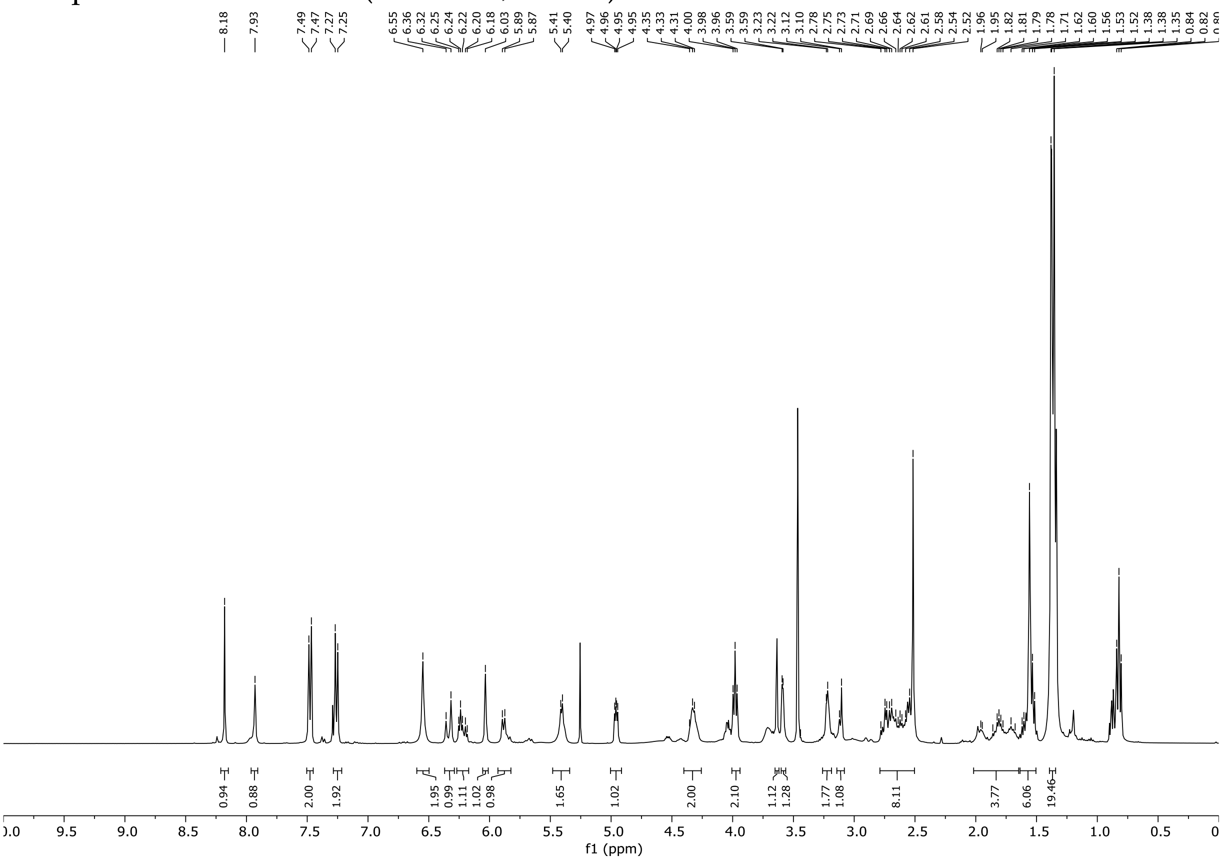

^{13}C NMR (101 MHz, $CDCl_3$)

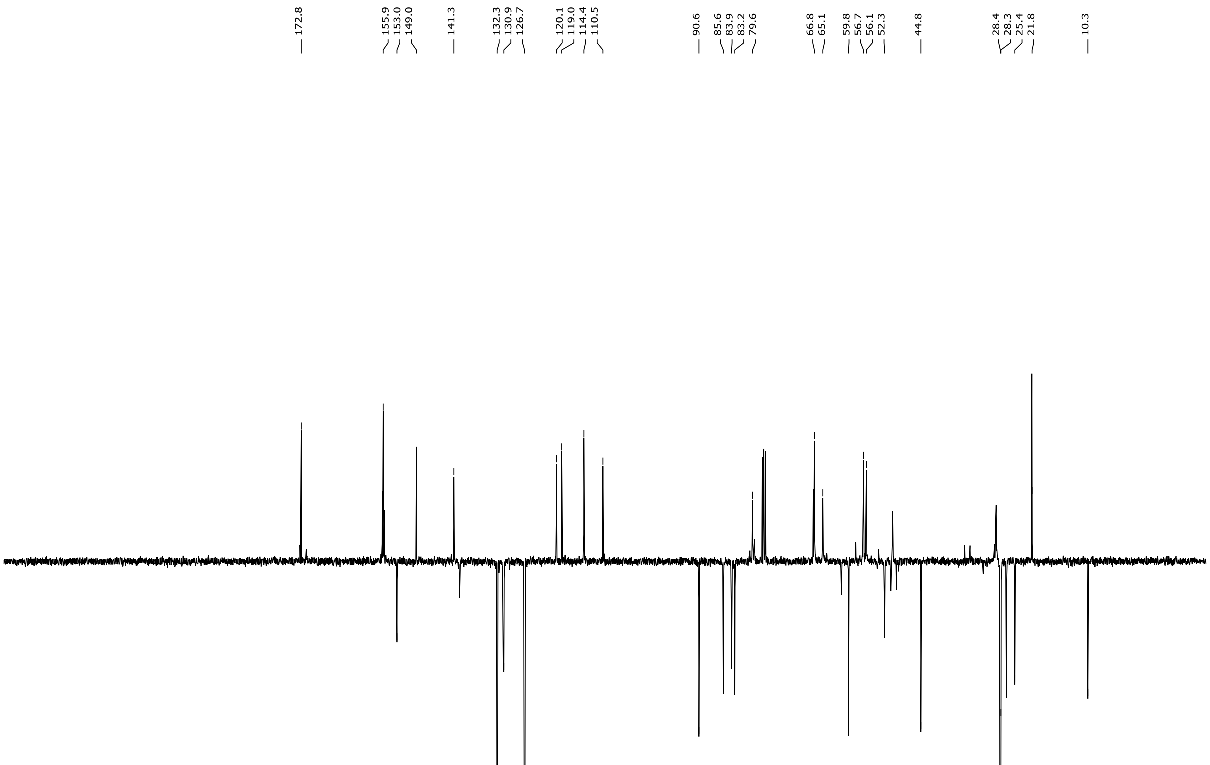

Compound **11e** ^{1}H NMR CDCl3 (400 MHz, CDCl3)

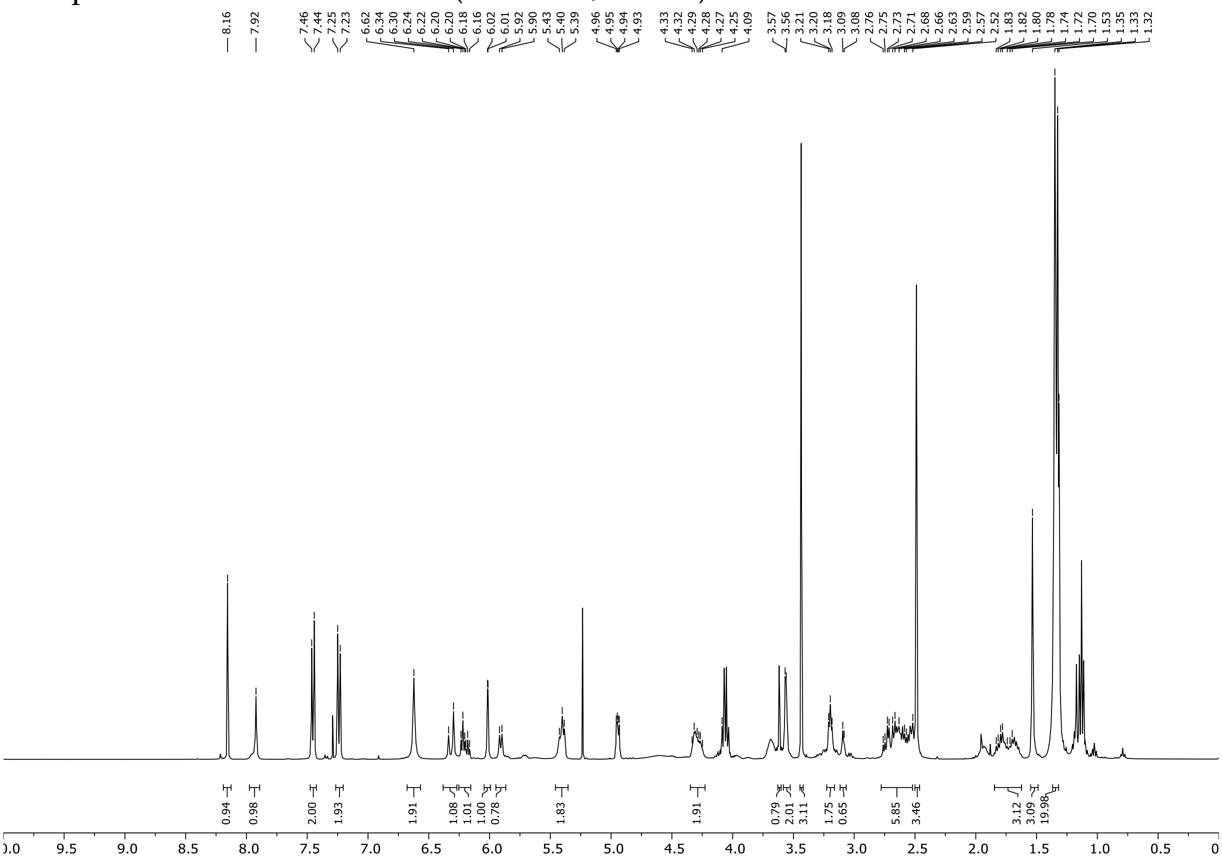

^{13}C NMR CDCl3 (101 MHz, CDCl3)

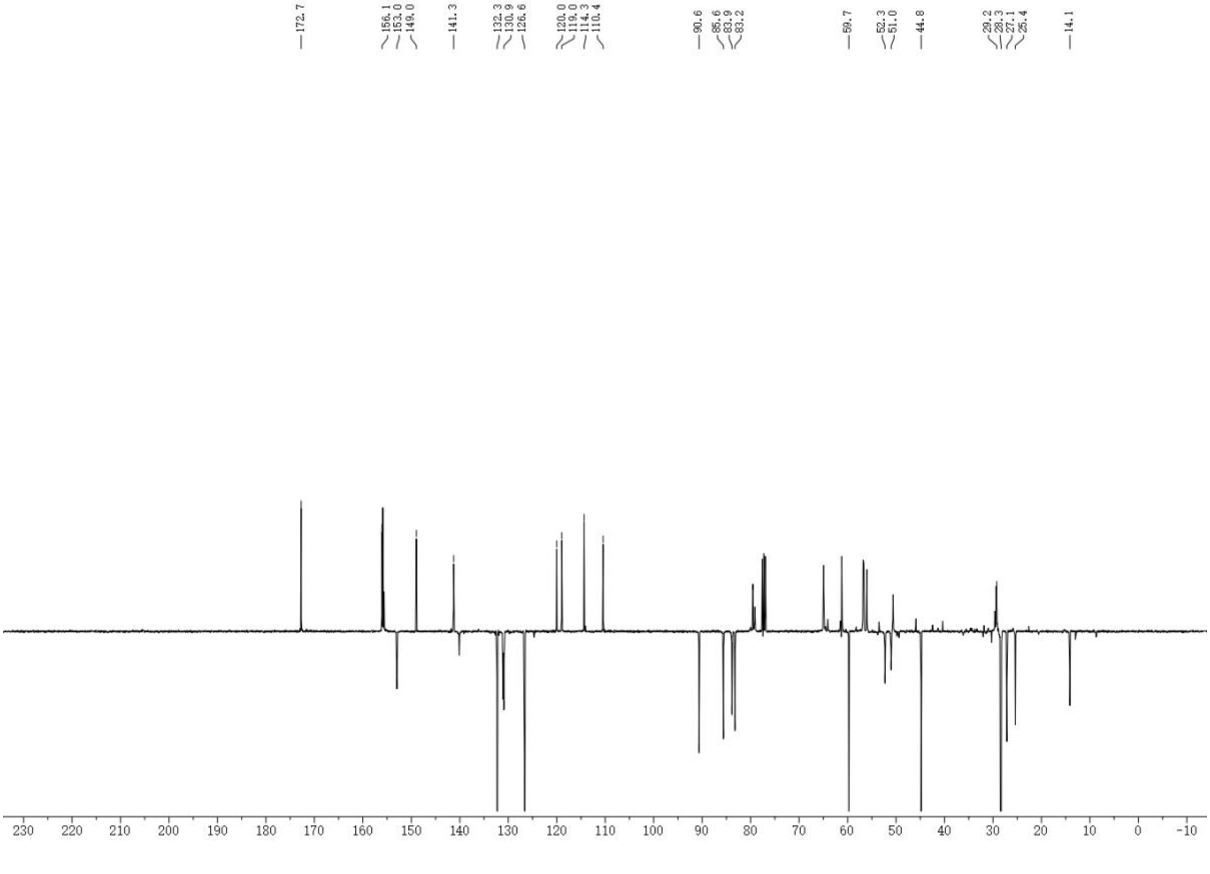

Compound **11f** ^{1}H NMR (400 MHz, $CDCl_3$)

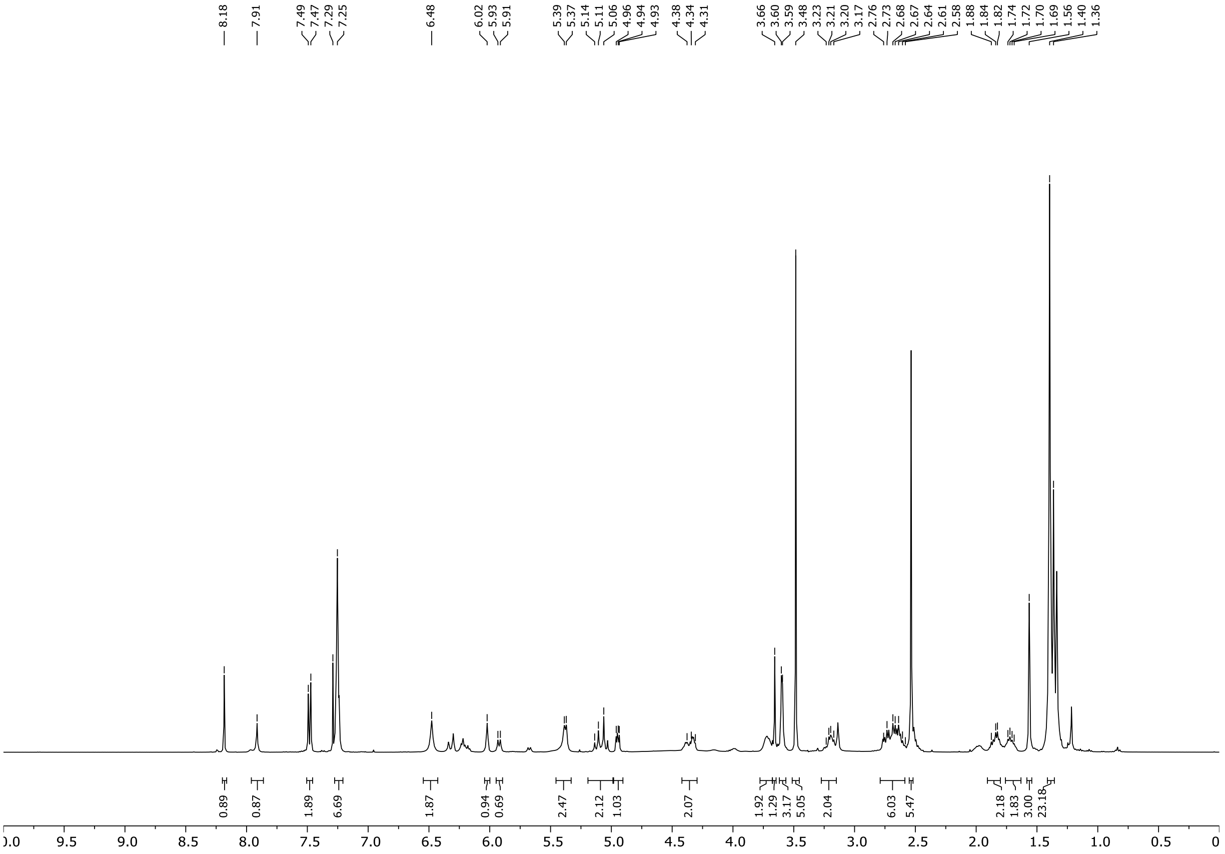

^{13}C NMR (400 MHz, $CDCl_3$)

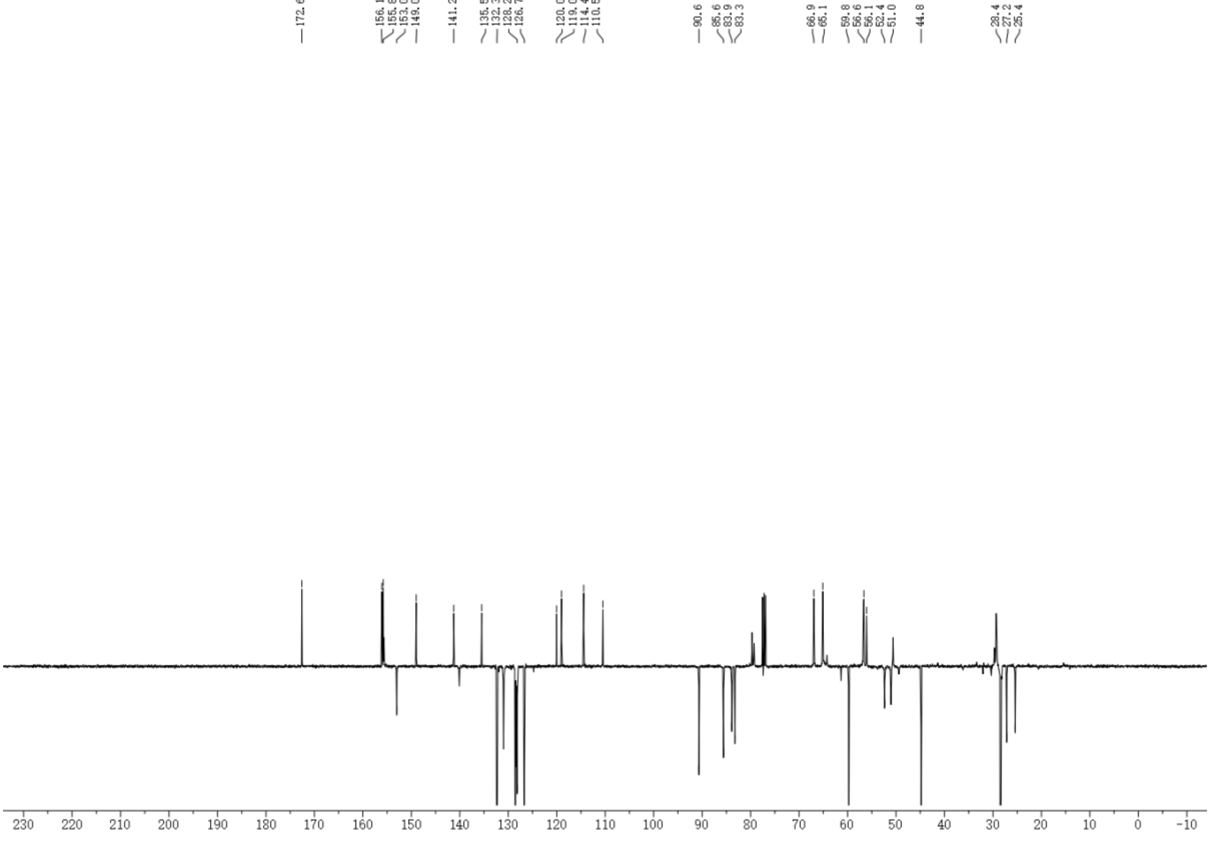

Compound **12b** ^{1}H NMR (400 MHz, CD_3OD)

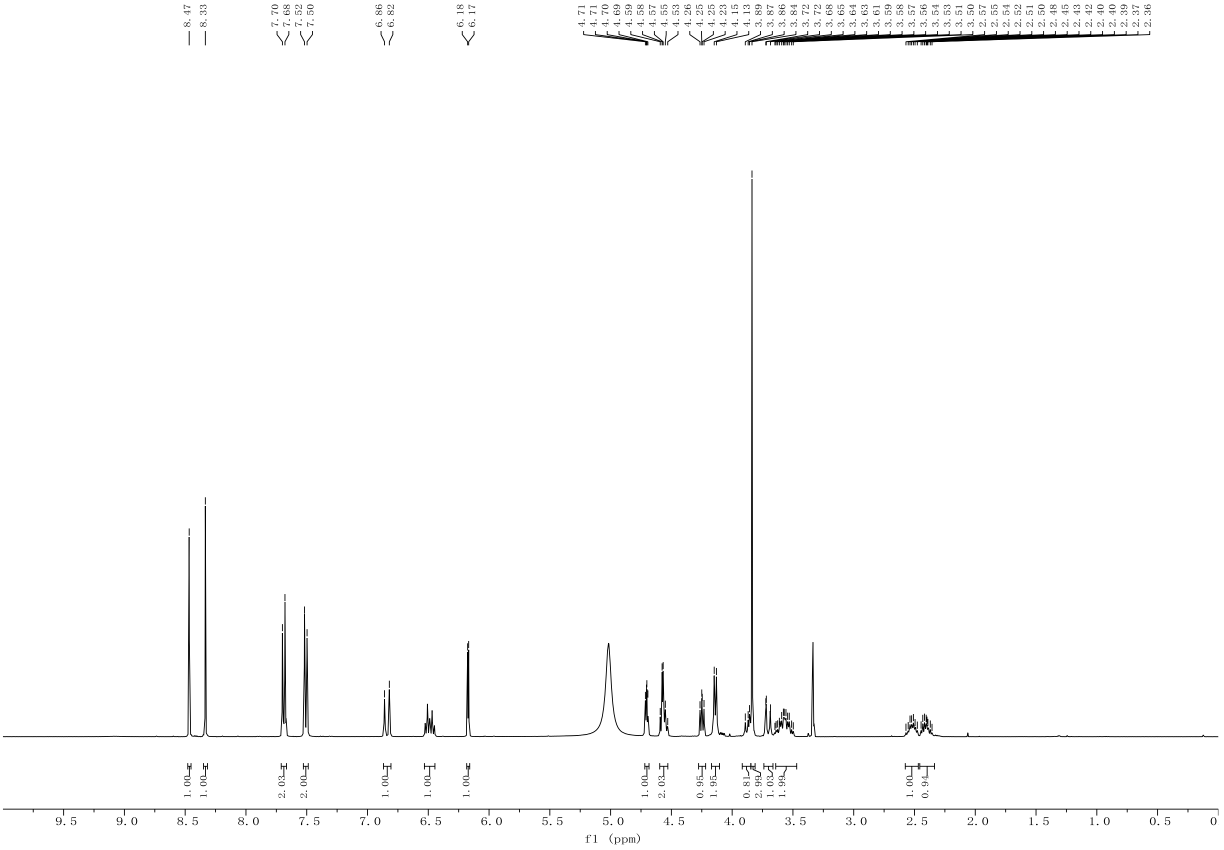

^{13}C NMR (101 MHz, CD_3OD)

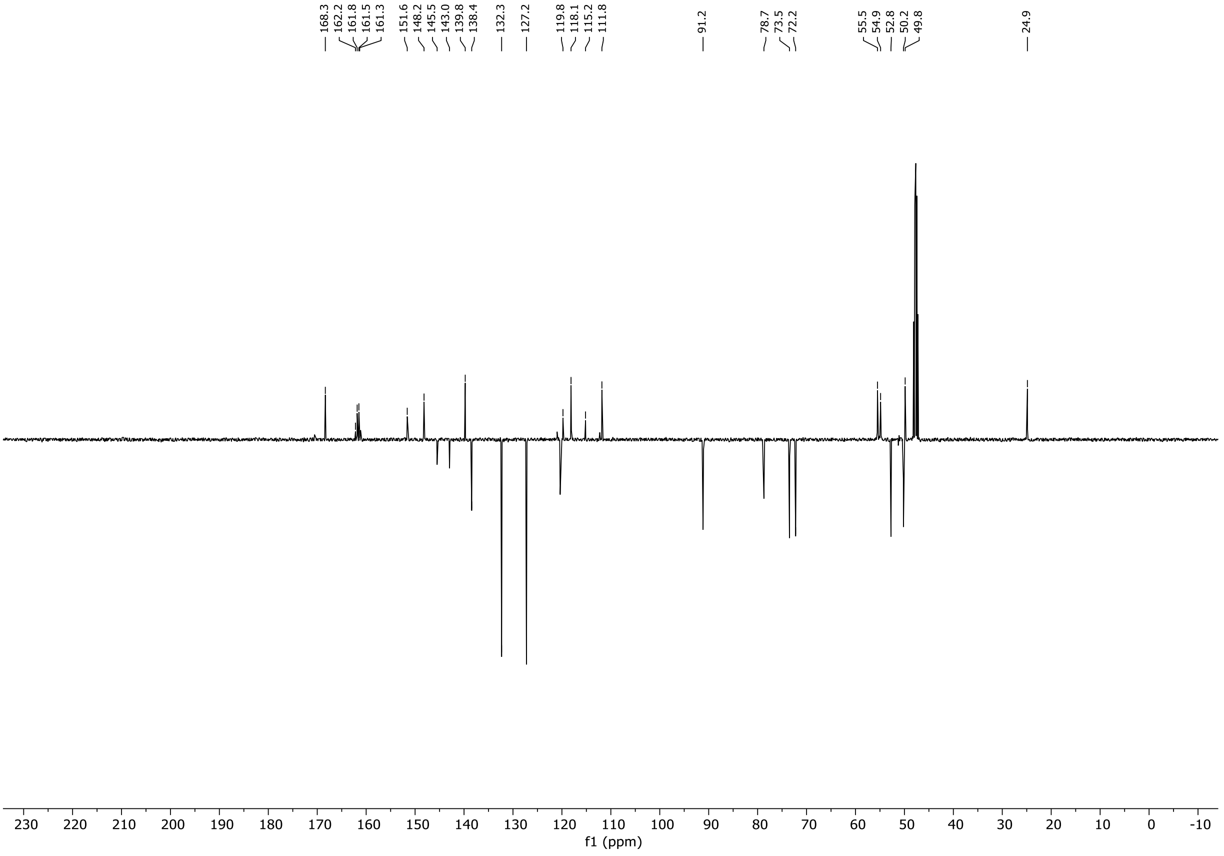

Compound **12c** ^{1}H NMR (400 MHz, CD_3OD)

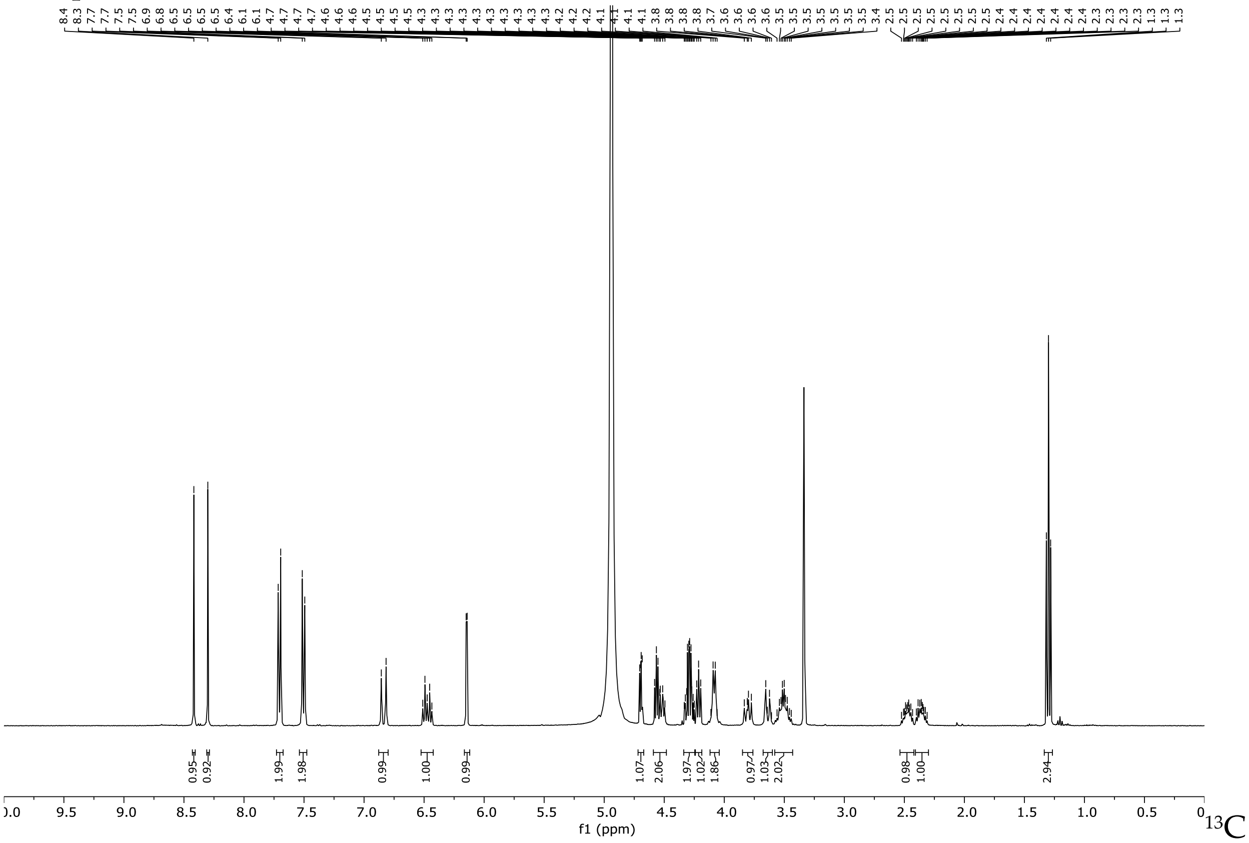

^{13}C NMR (101 MHz, CD_3OD)

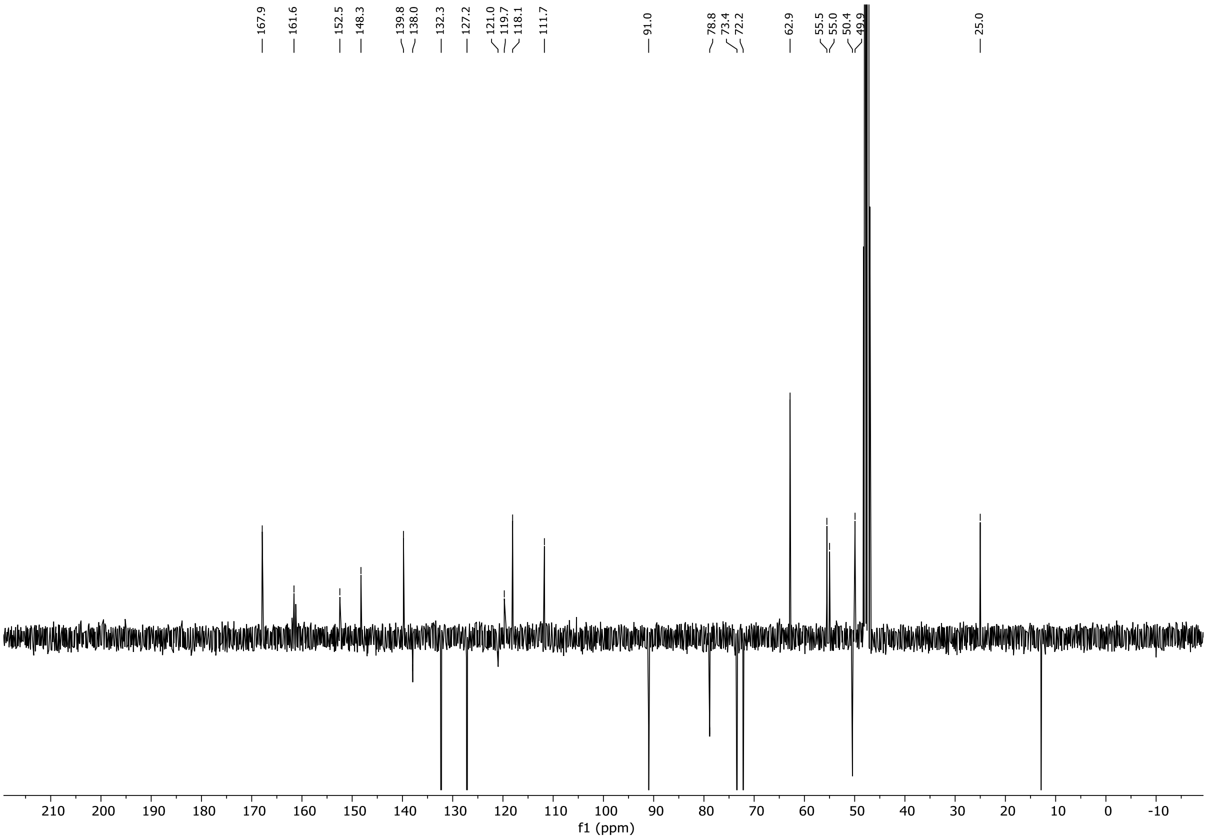

Compound **12d** ^{1}H NMR (400 MHz, CD_3OD)

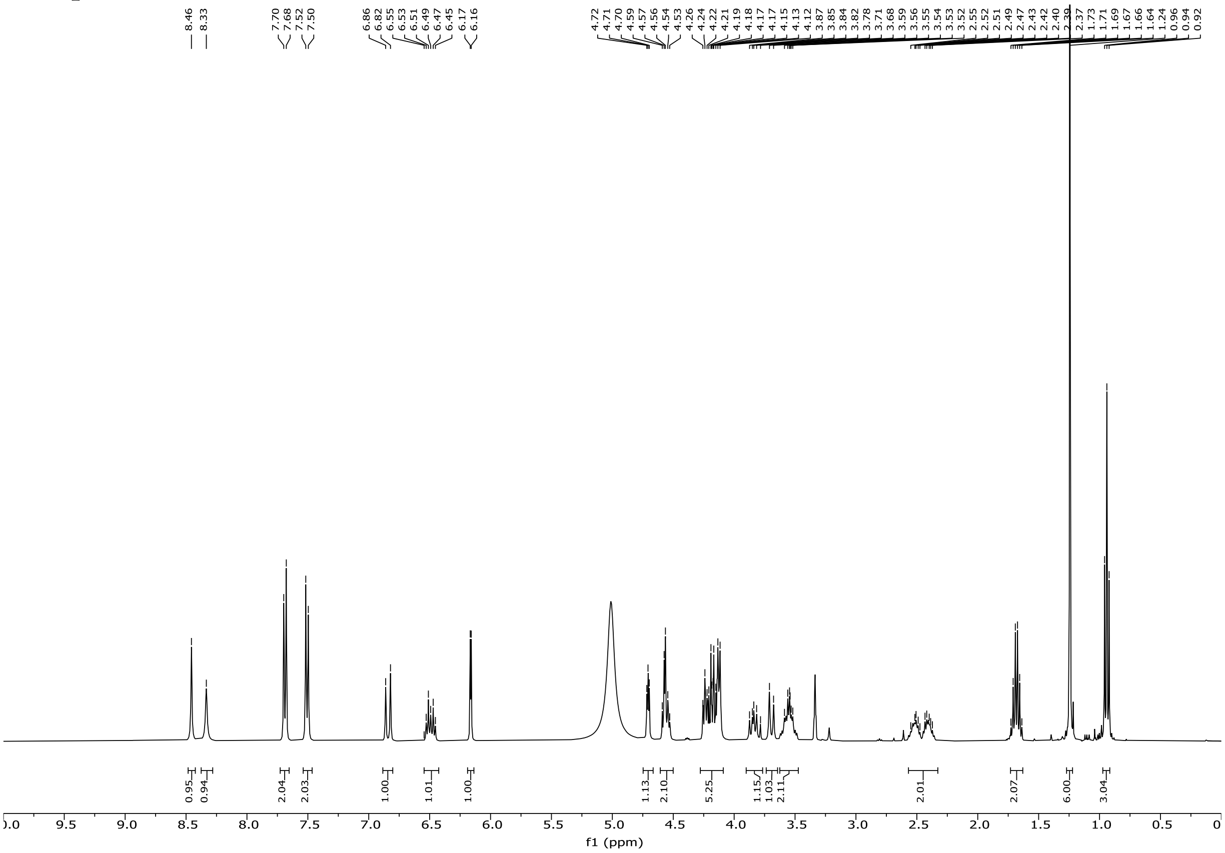

^{13}C NMR (101 MHz, CD_3OD)

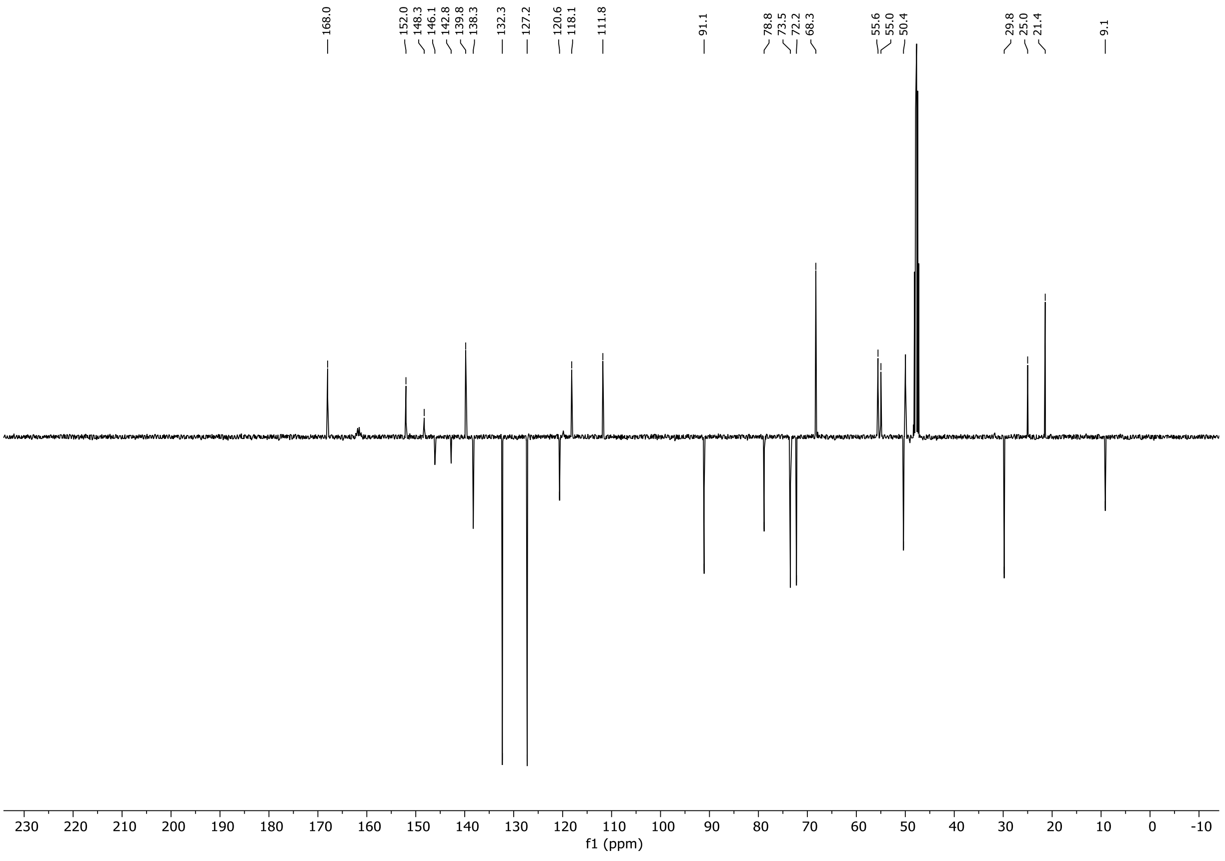

Compound **12e** ^{1}H NMR (400 MHz, CD_3OD)

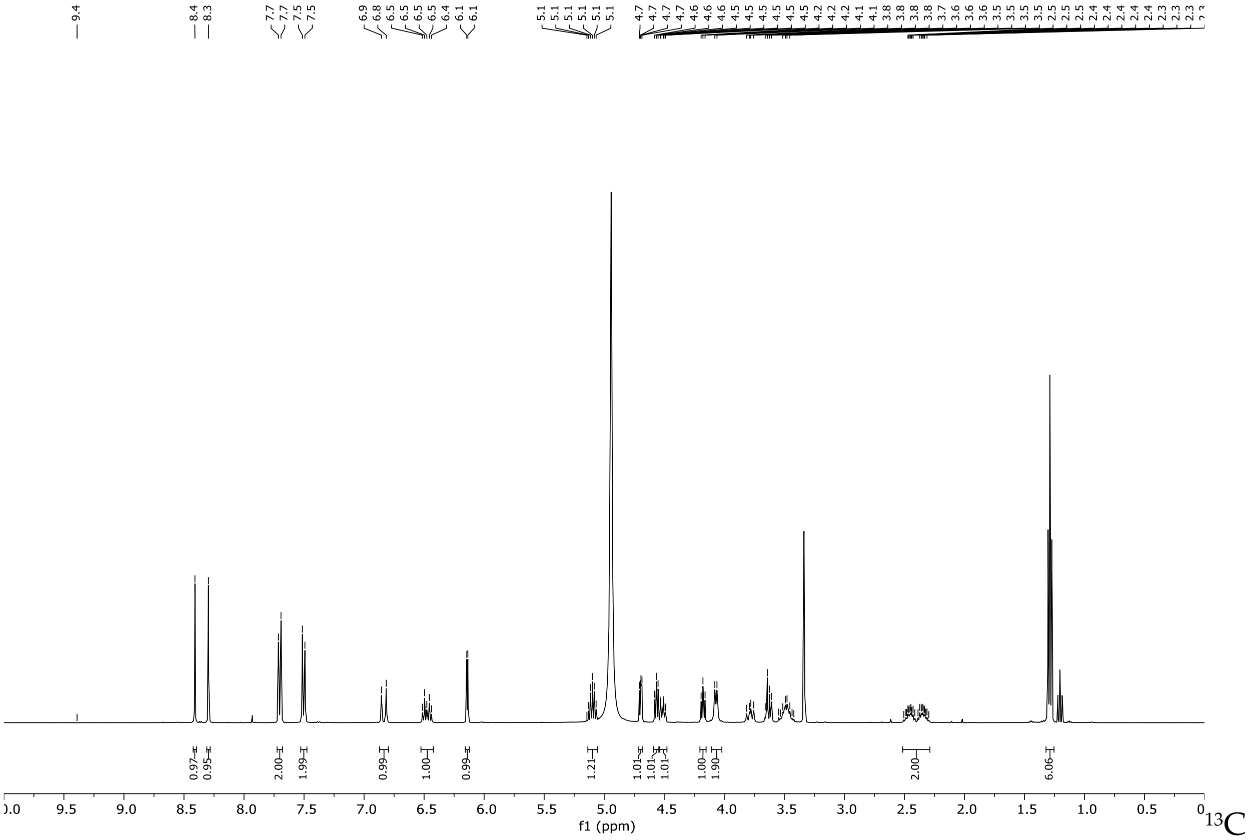

^{13}C NMR (101 MHz, CD_3OD)

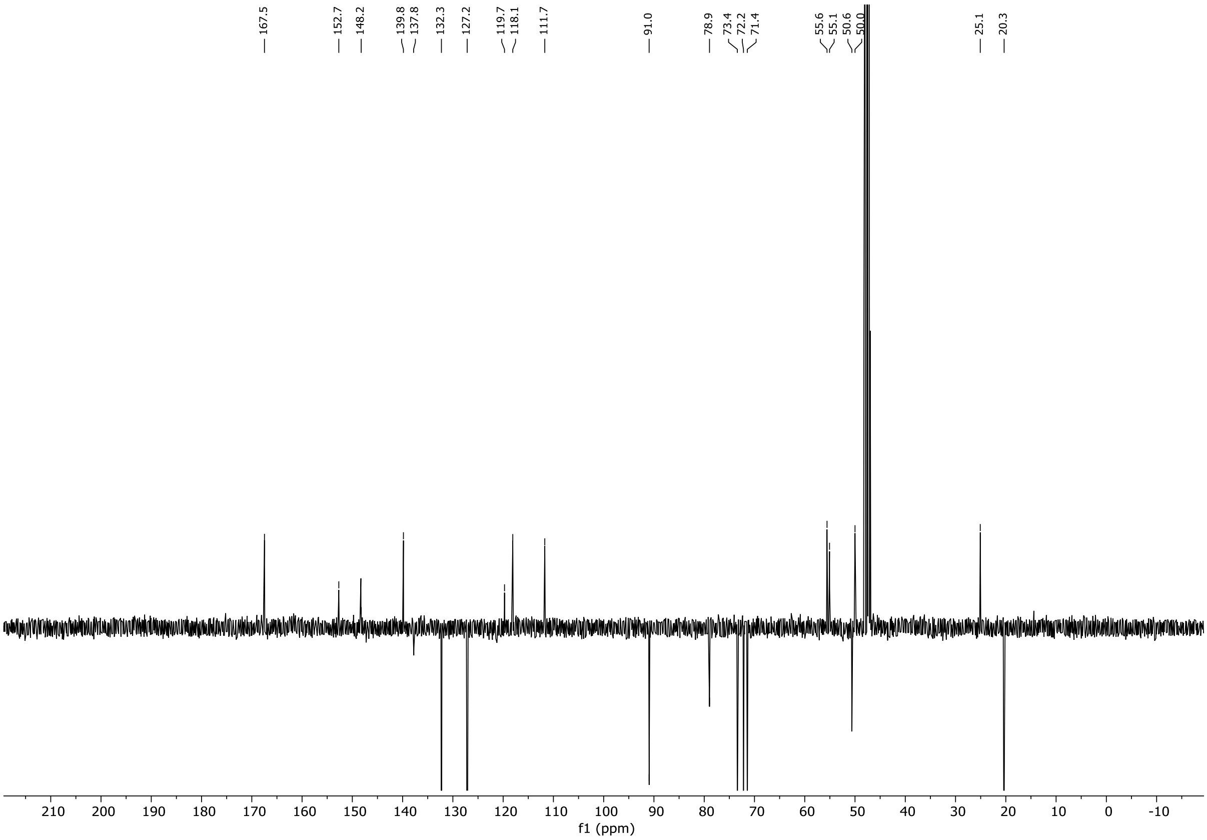

Compound **12f** ^{1}H NMR (400 MHz, CD_3OD)

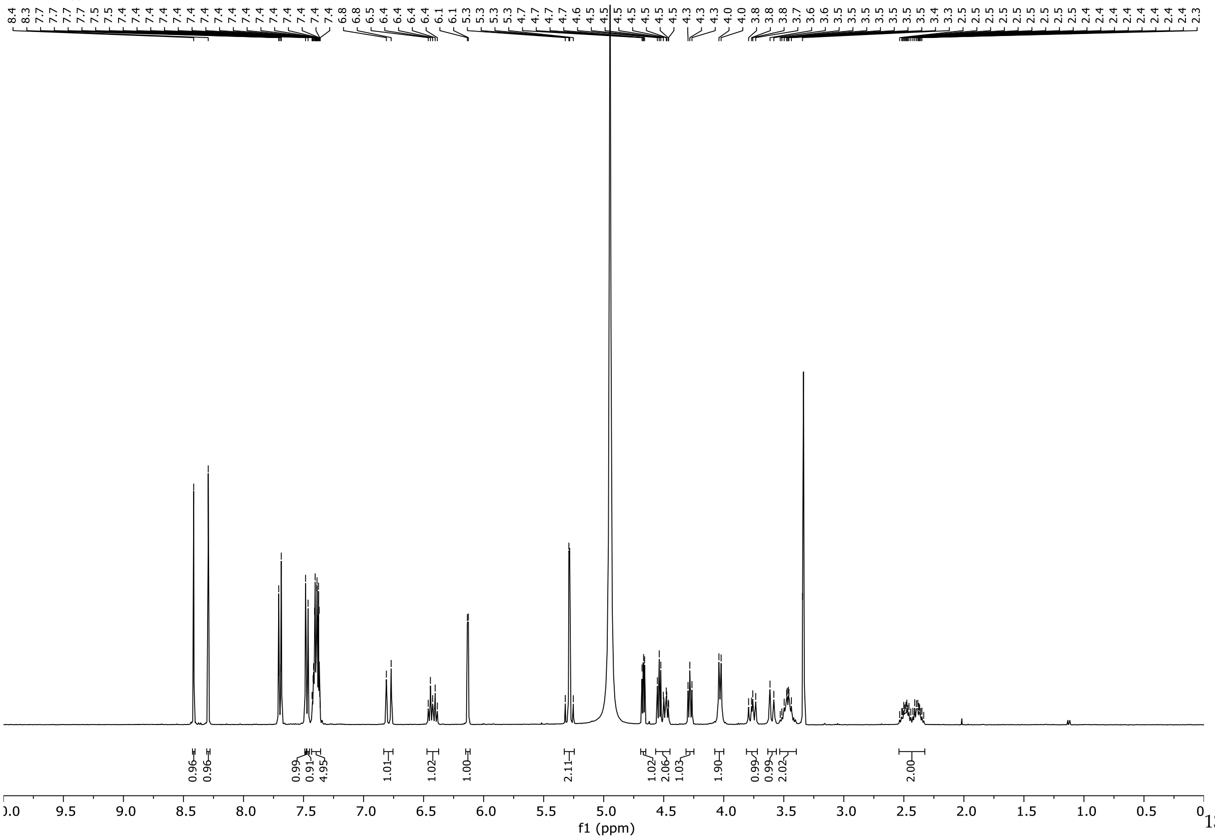

^{13}C NMR (101 MHz, CD_3OD)

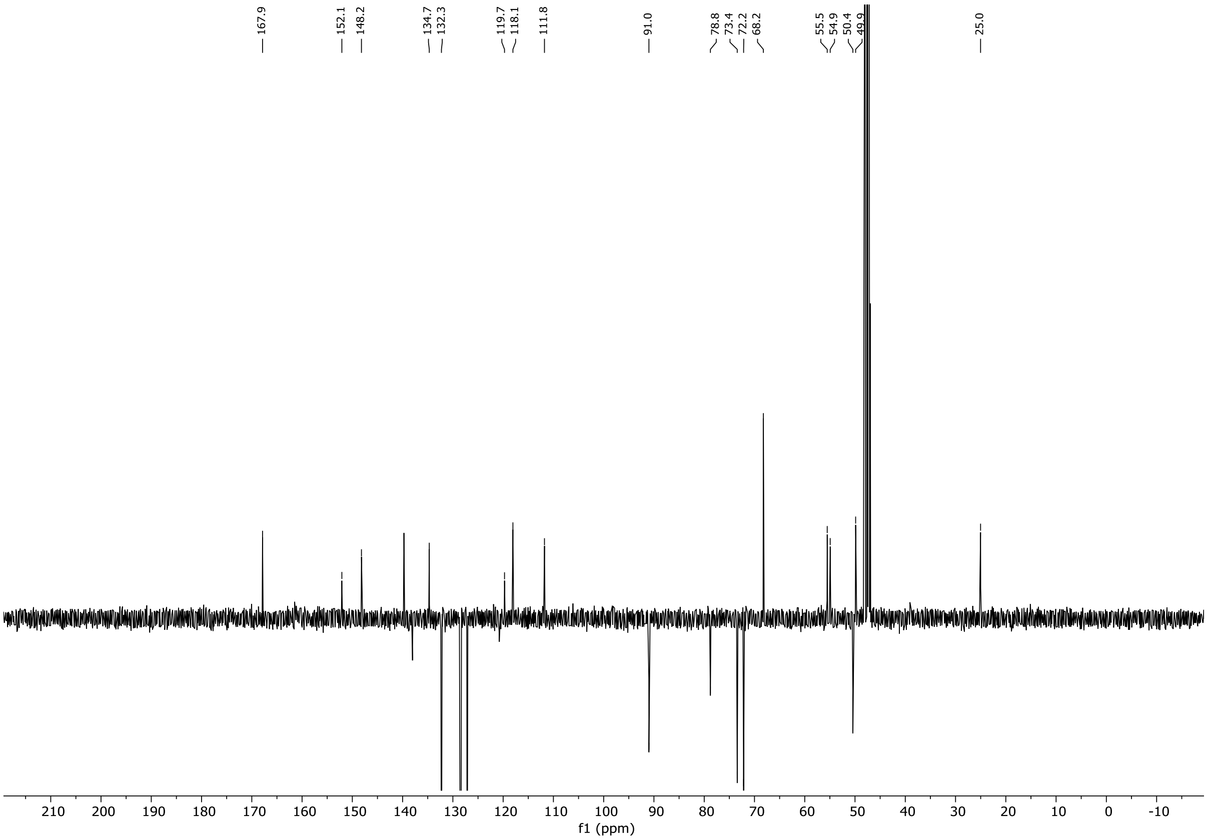

Compound **13a** ^{1}H NMR (400 MHz, $CDCl_3$)

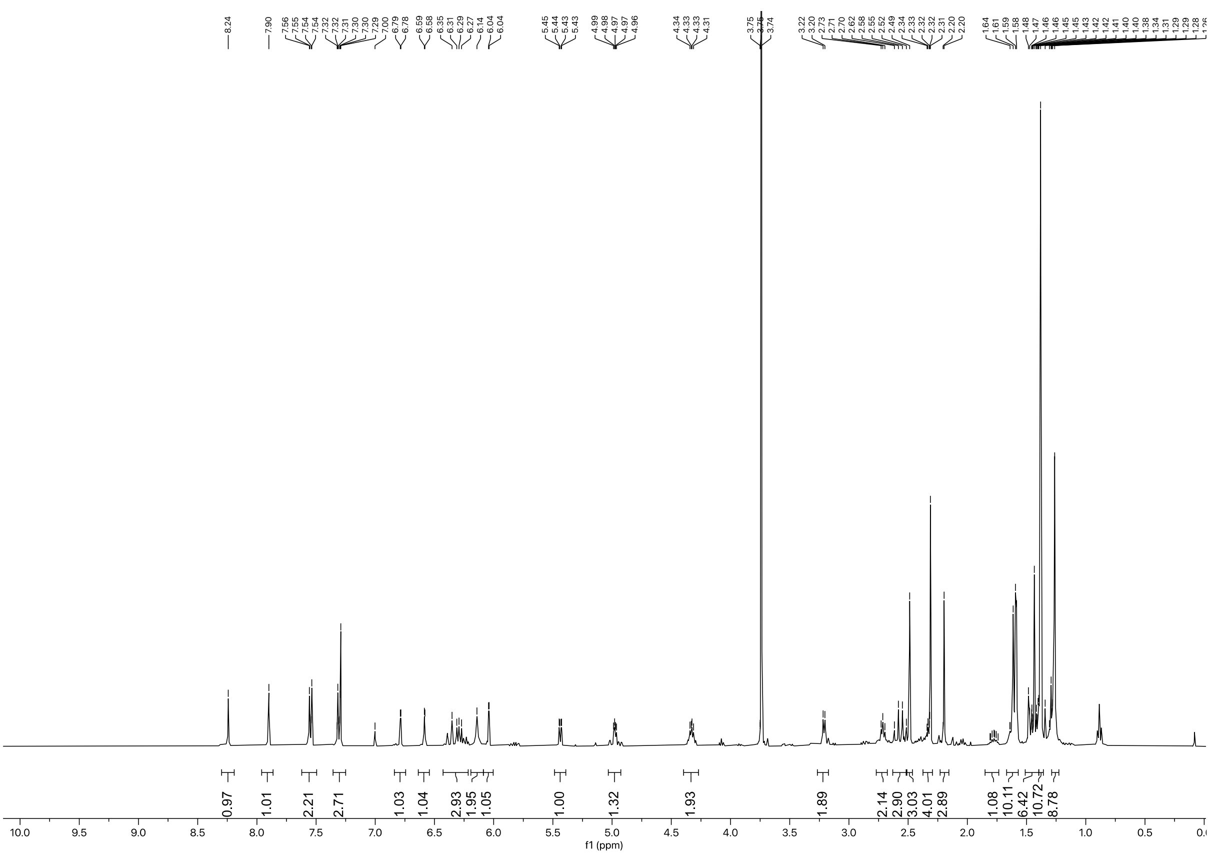

^{13}C NMR (101 MHz, $CDCl_3$)

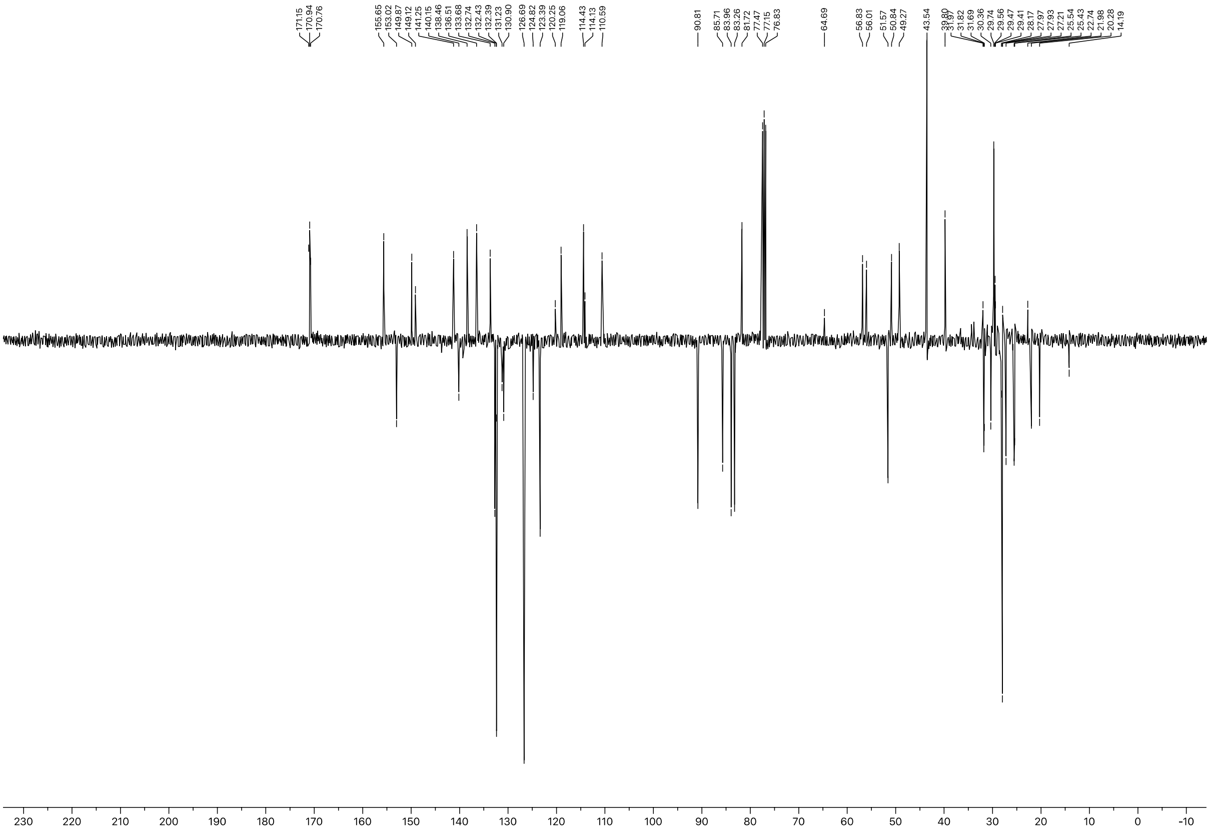

Compound **13b** ^{1}H NMR (400 MHz, $CDCl_3$)

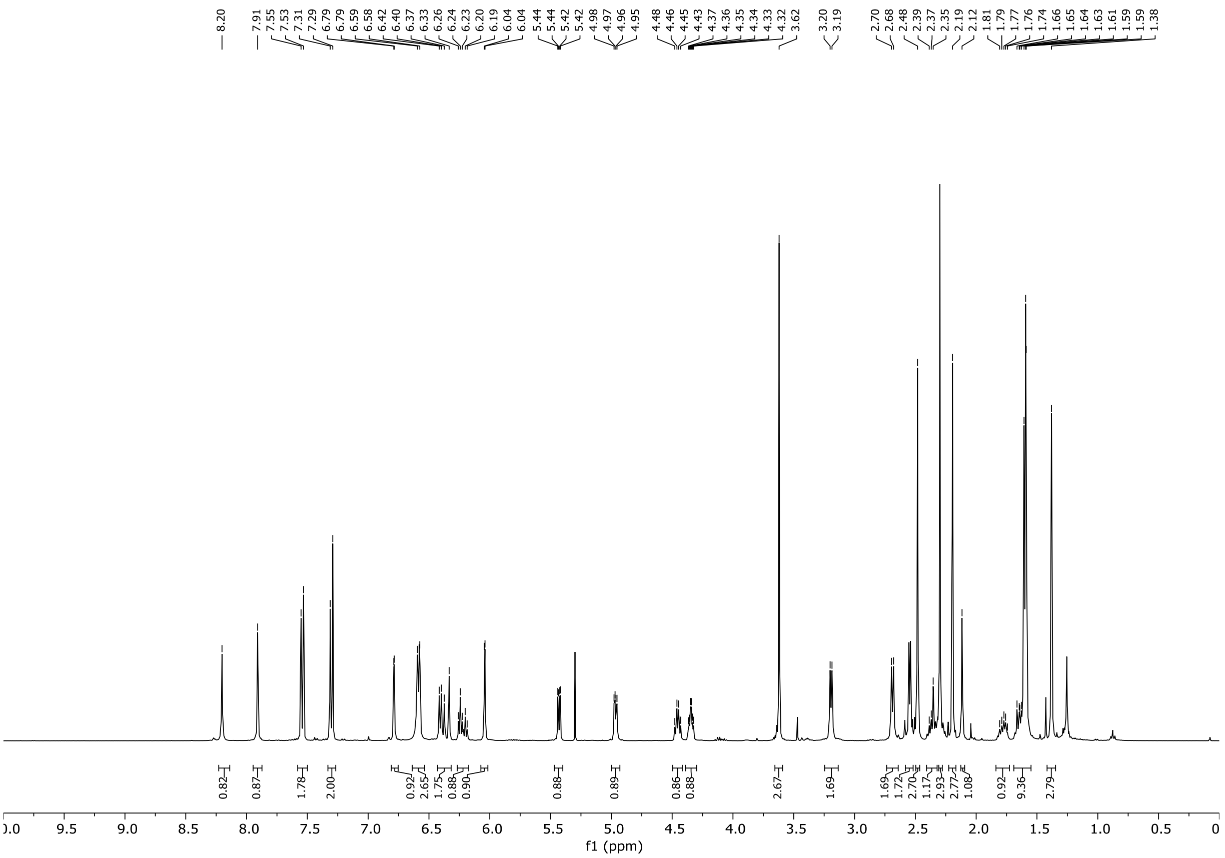

^{13}C NMR (101 MHz, $CDCl_3$)

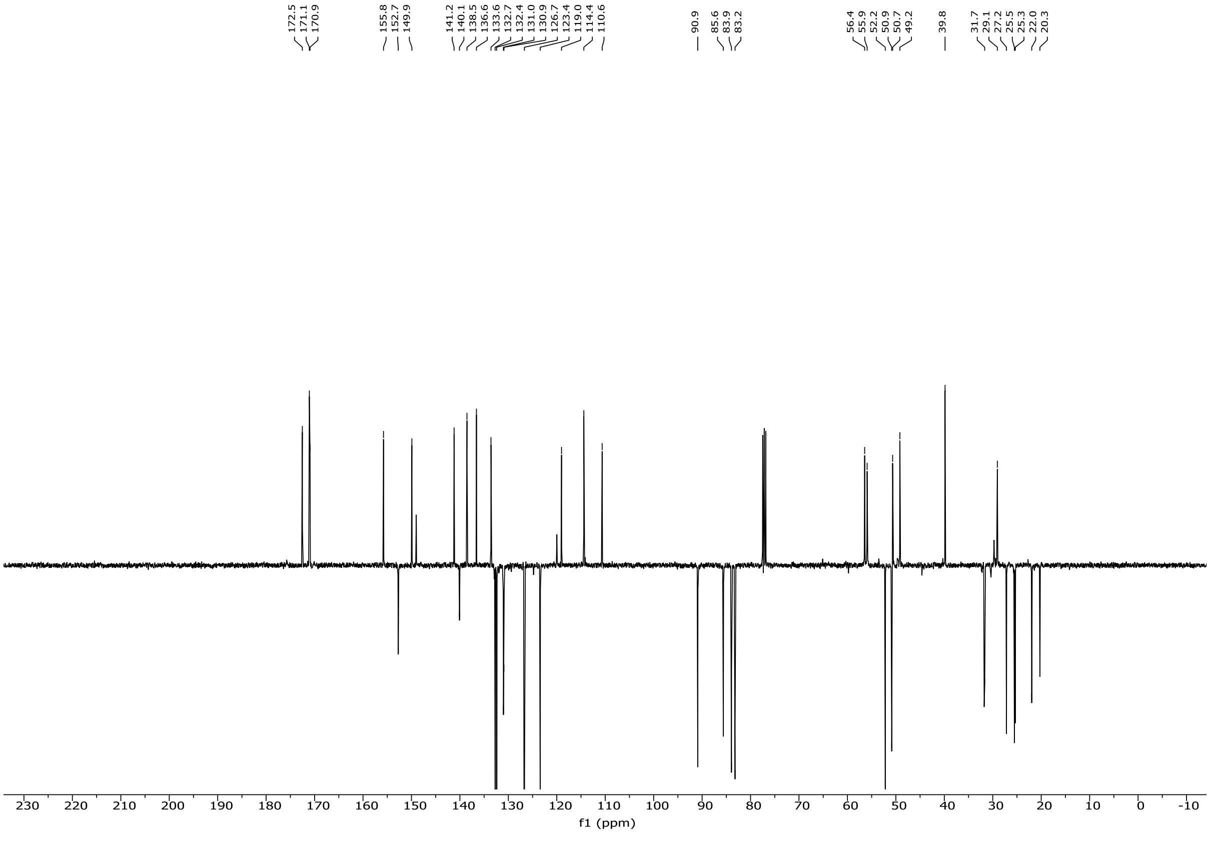

Compound 13c ^{1}H NMR (400 MHz, $CDCl_3$)

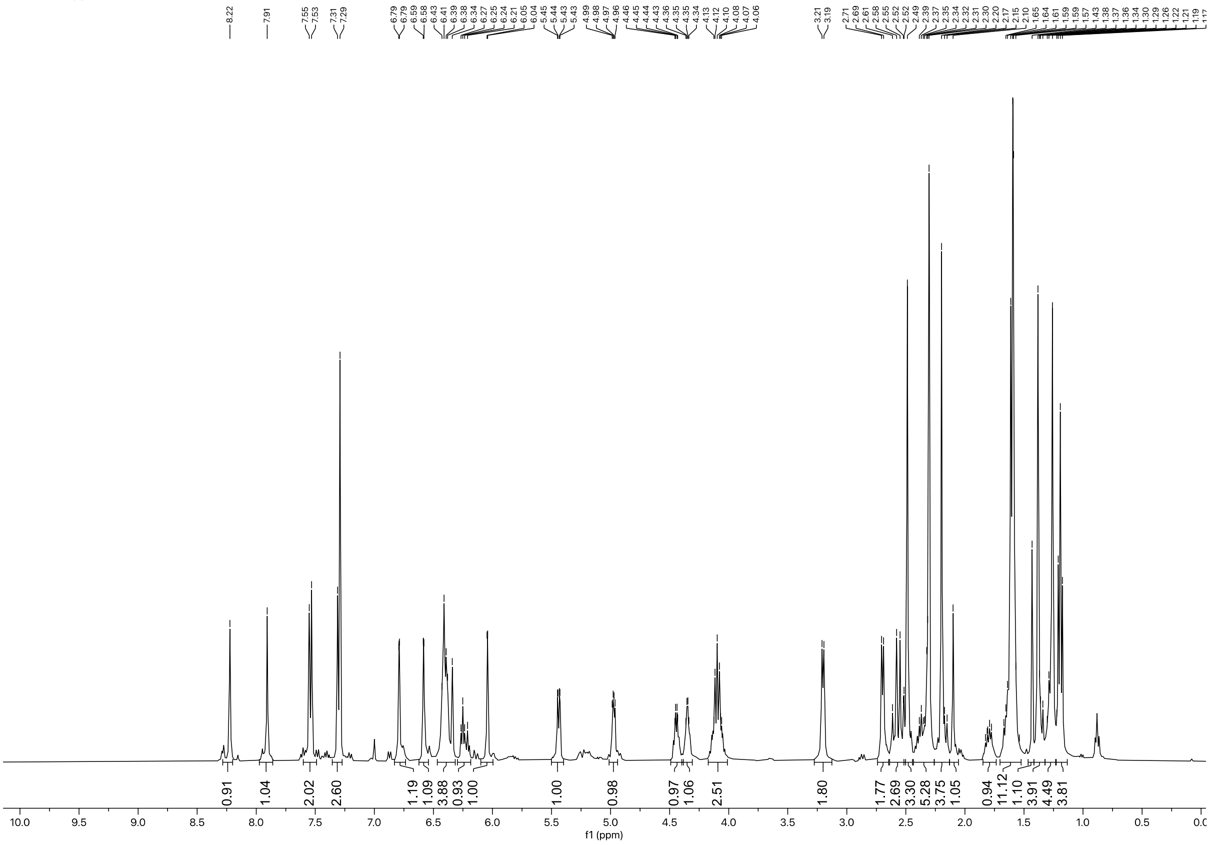

^{13}C NMR (101 MHz, $CDCl_3$)

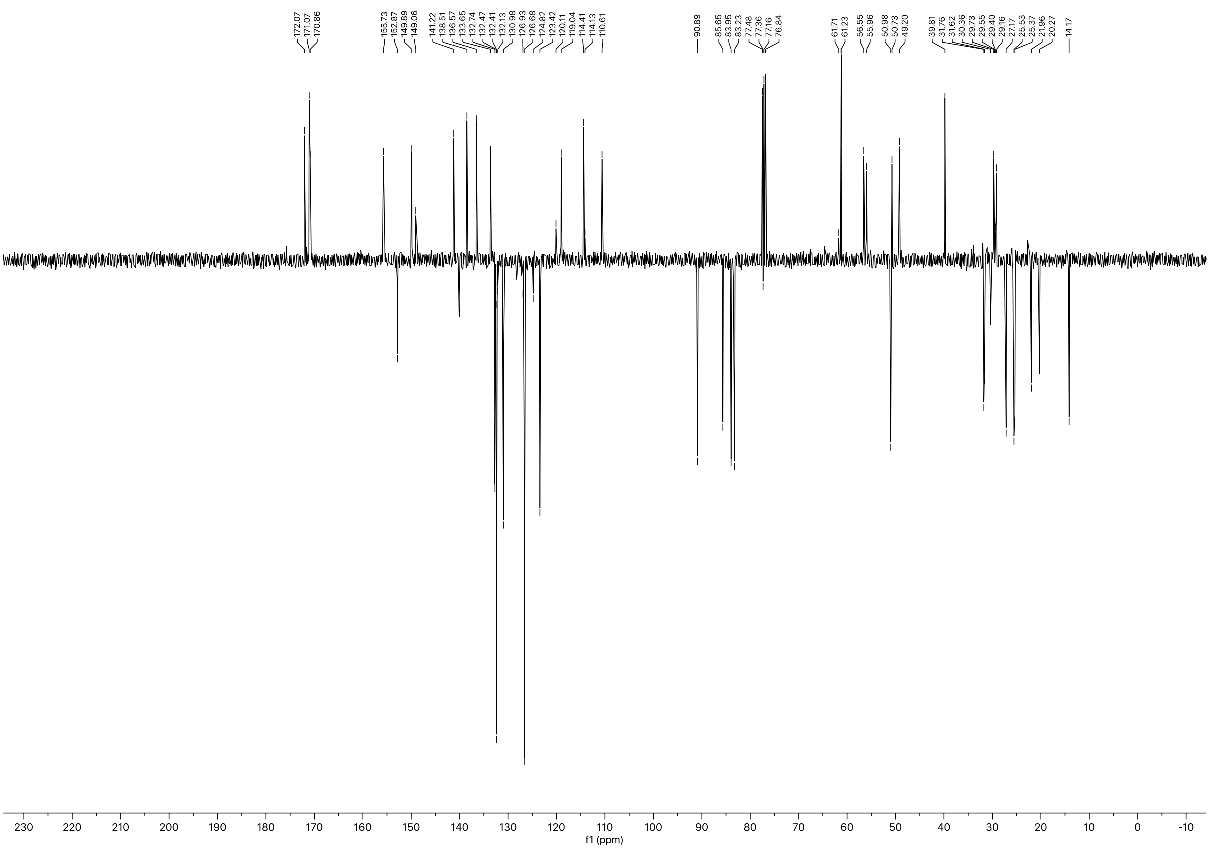

Compound 13e ¹H (400 MHz, CDCl₃)

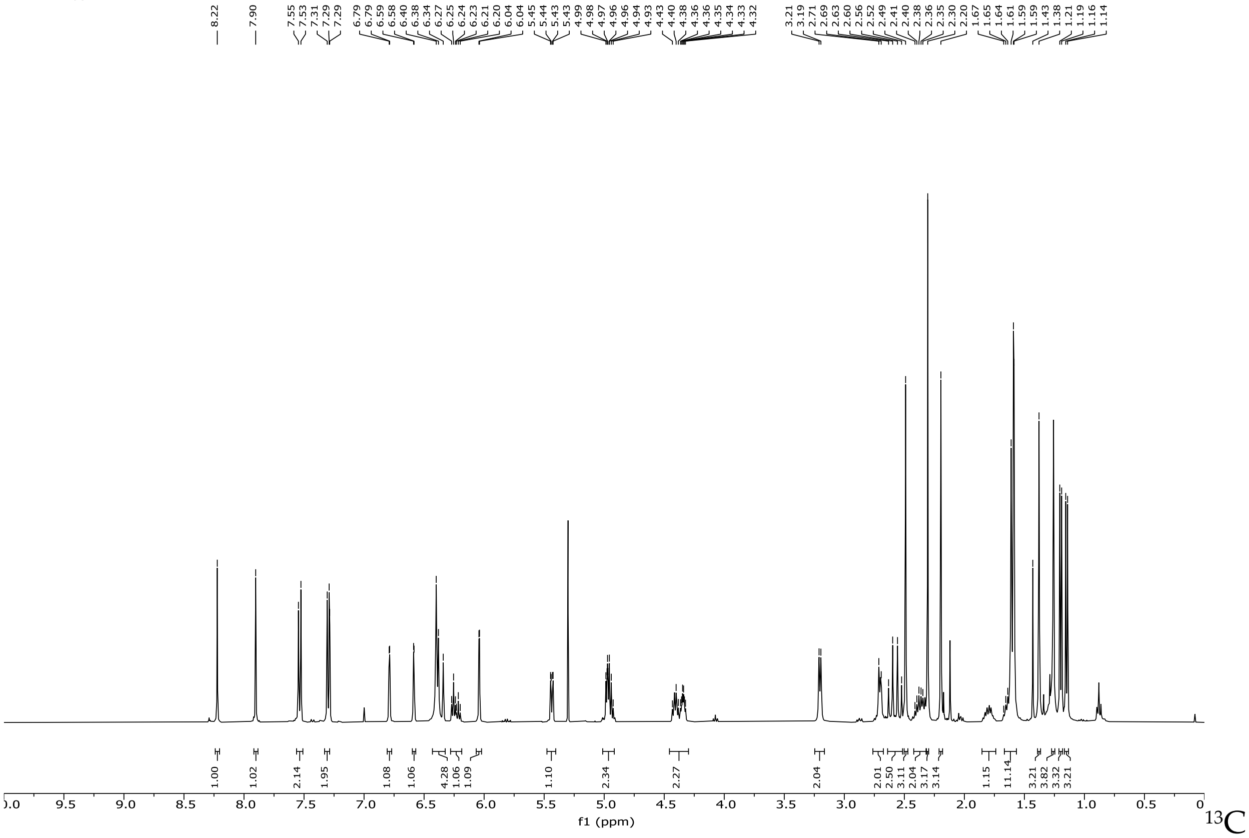

¹³C NMR (101 MHz, CDCl₃)

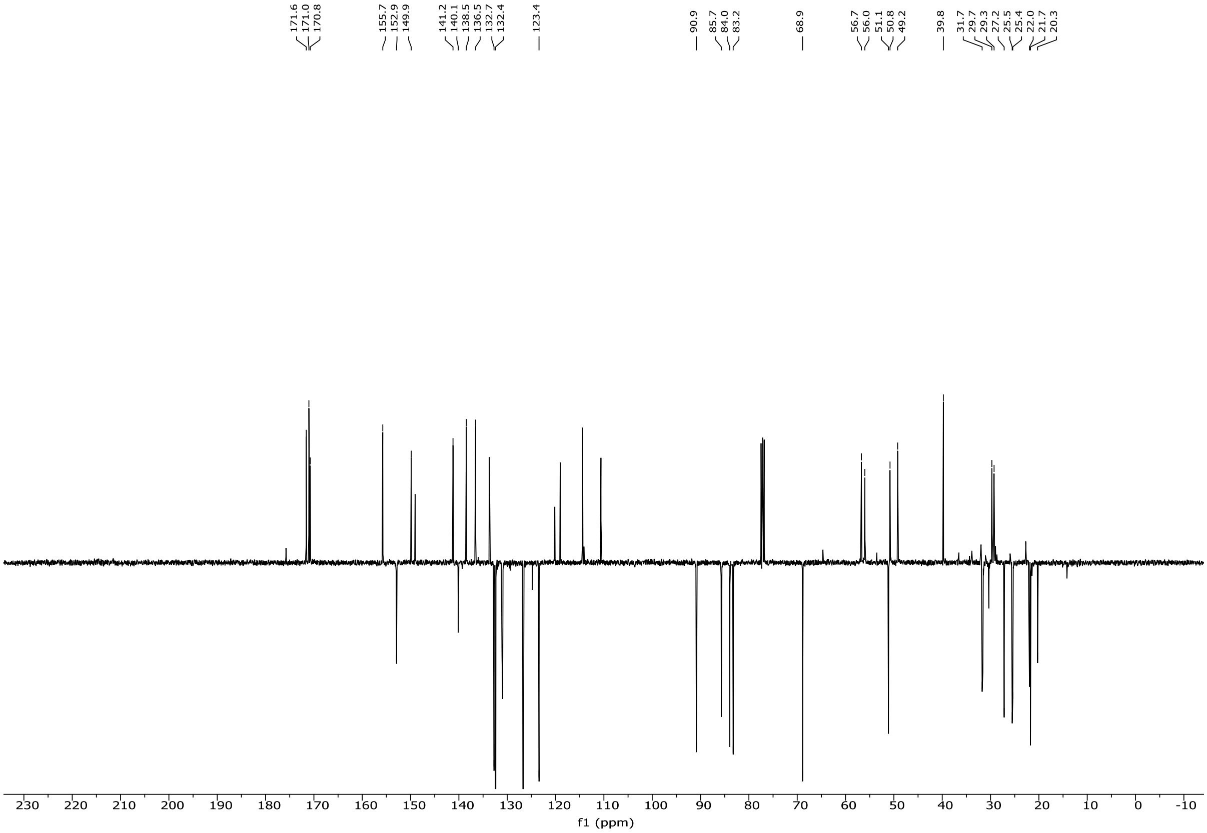

Compound **14a** ^{1}H (400 MHz, $CDCl_3$)

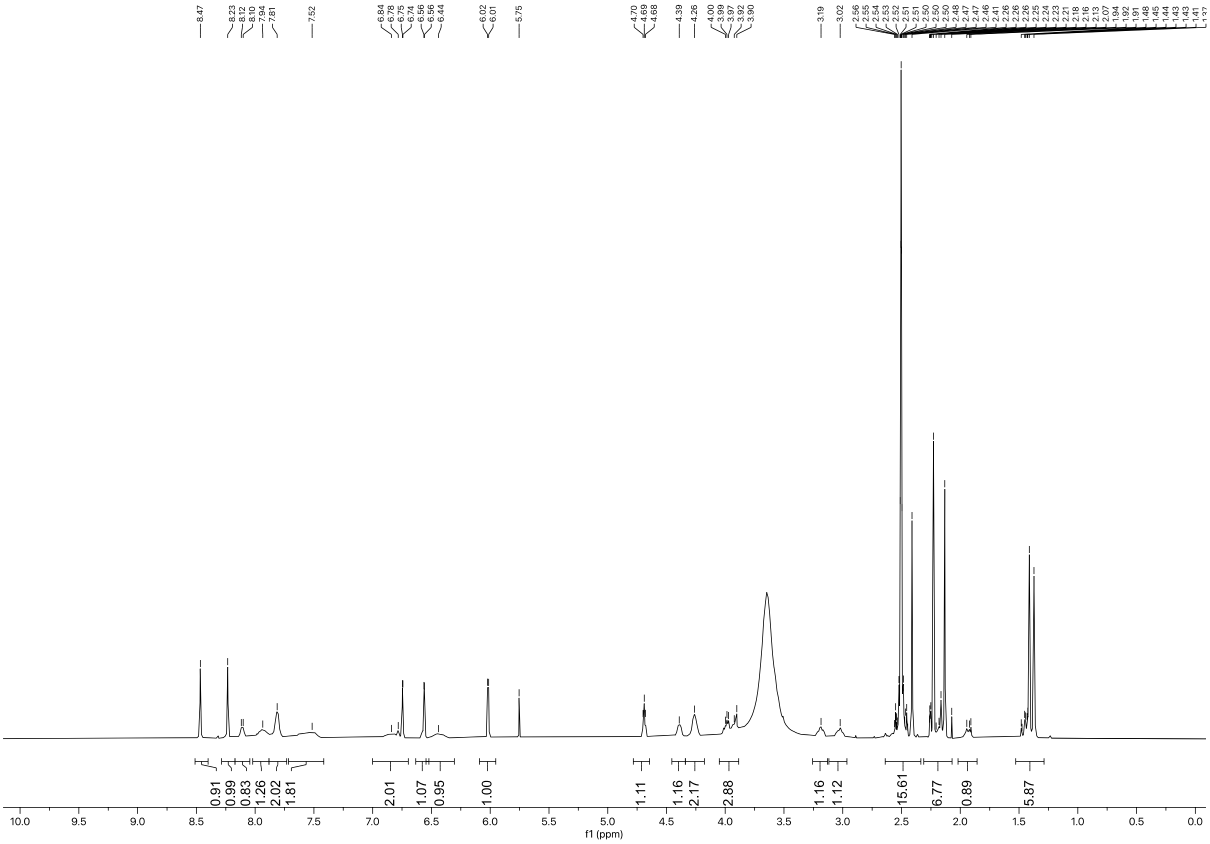

^{13}C NMR (101 MHz, $CDCl_3$)

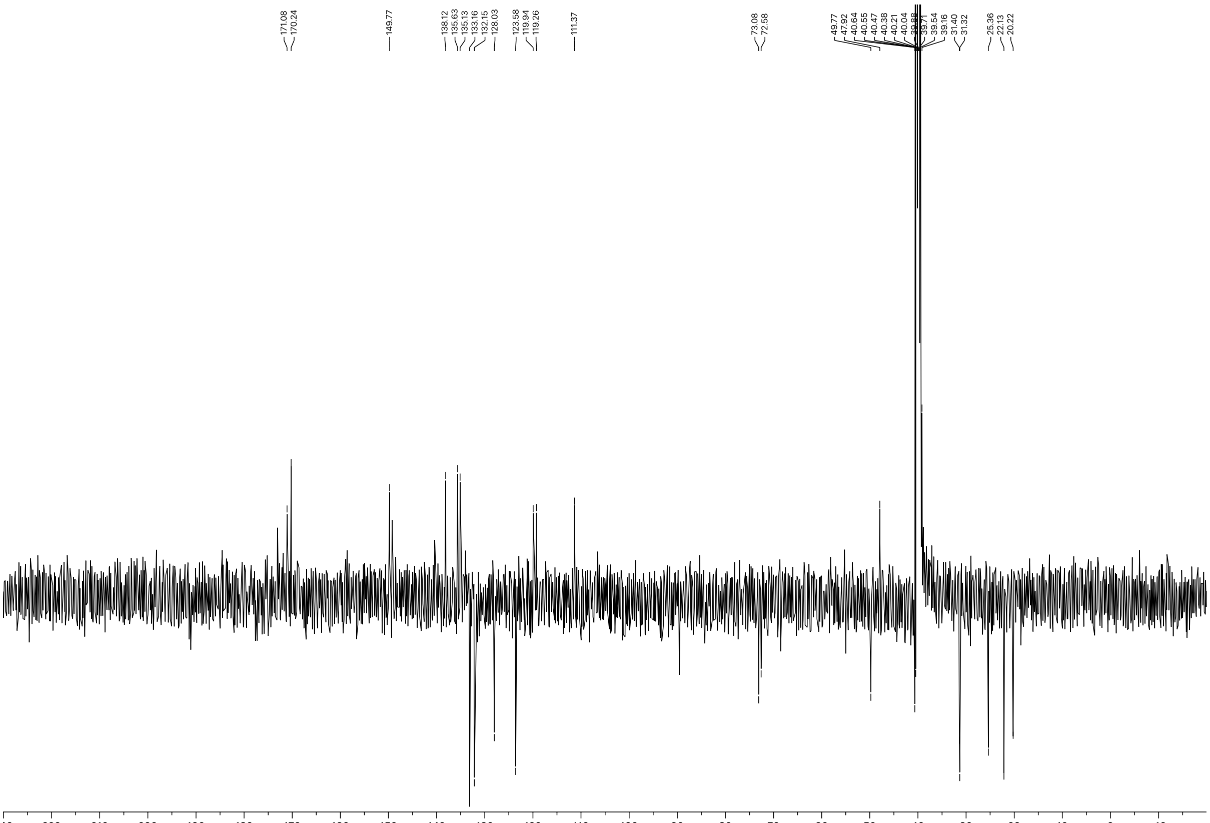

Compound **14b** ^{1}H NMR (400 MHz, CD_3OD)

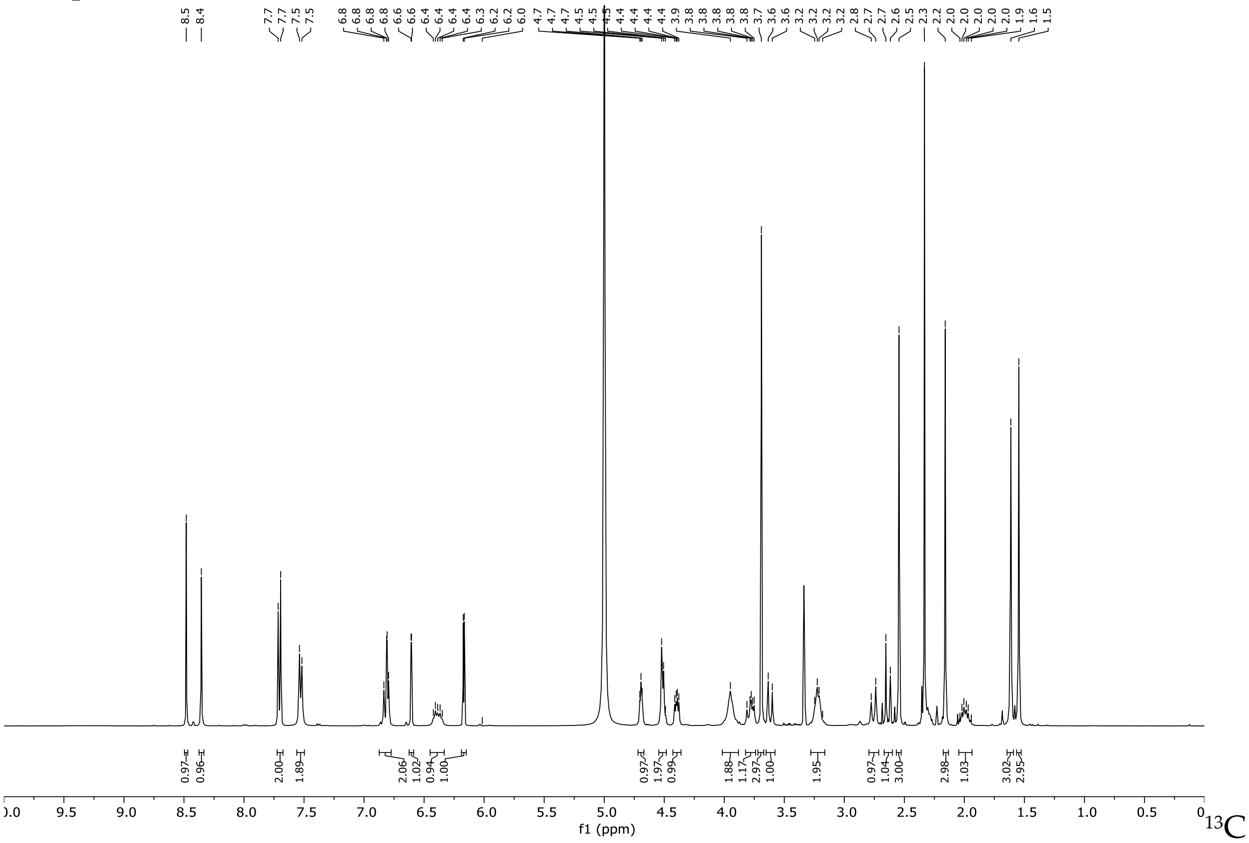

^{13}C NMR (400 MHz, CD_3OD)

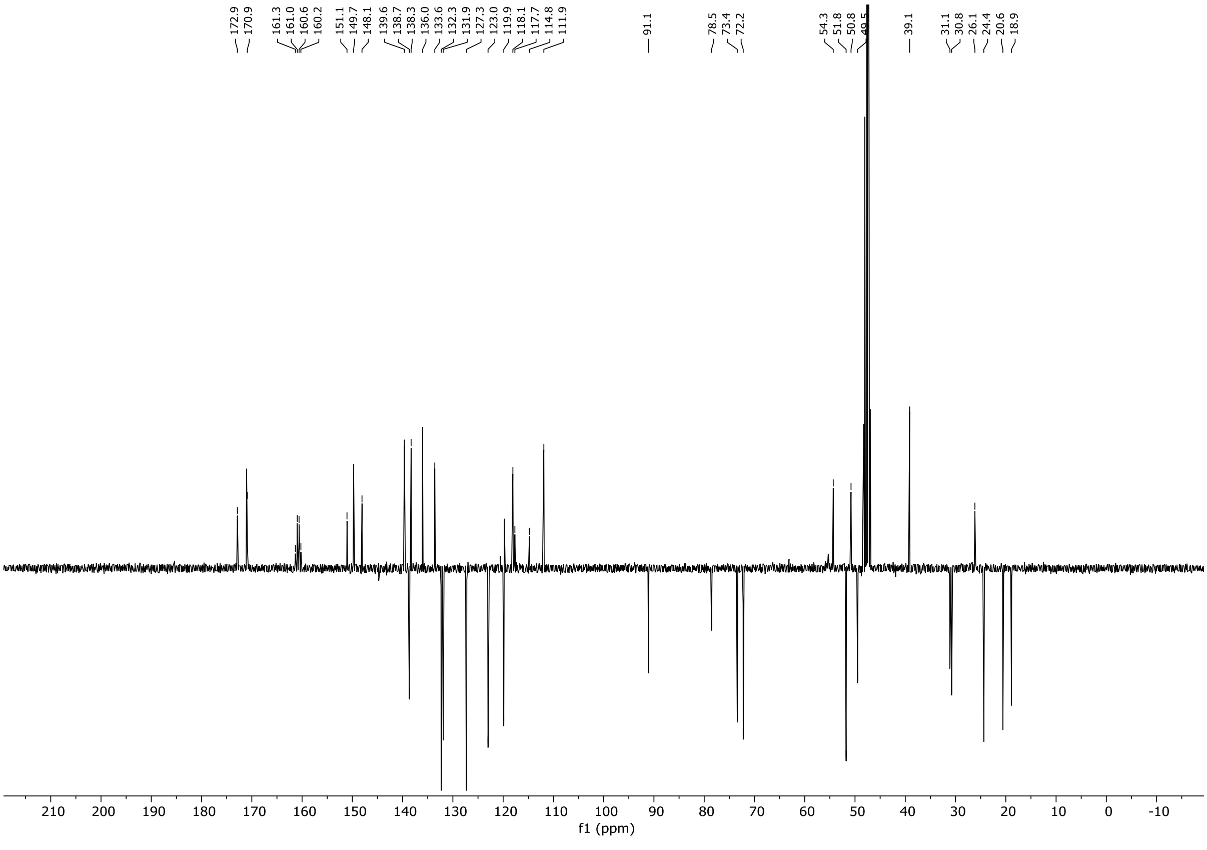

Compound **14c** ^{1}H NMR (400 MHz, CD_3OD)

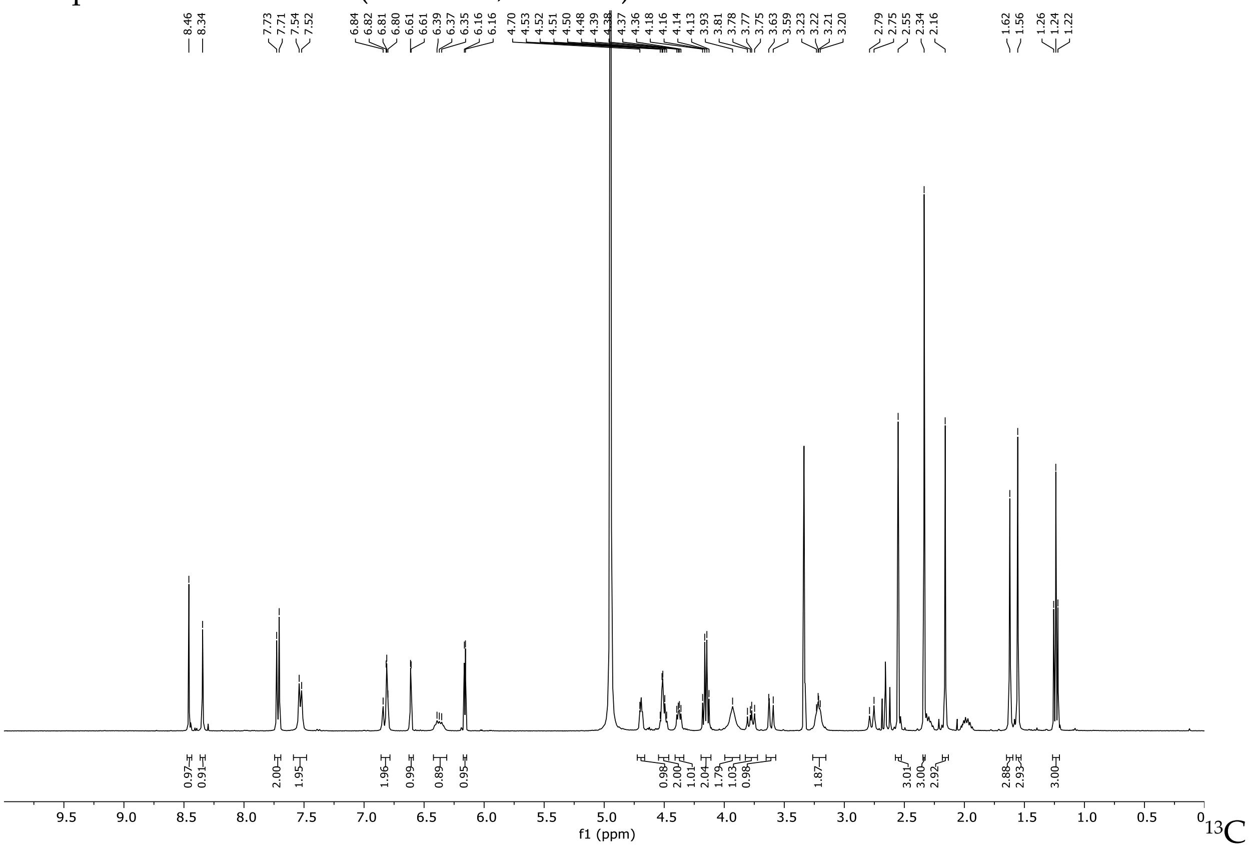

^{13}C NMR (400 MHz, CD_3OD)

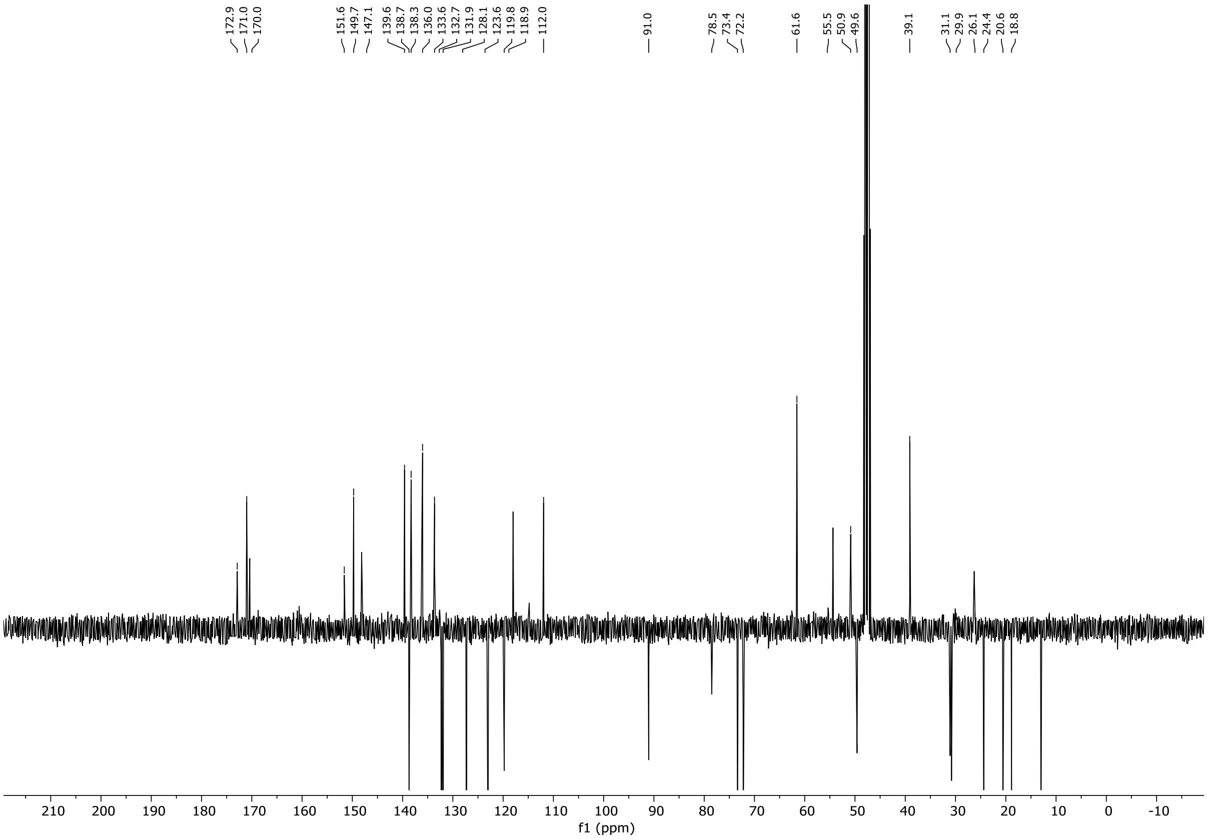

Compound **14d** ^{1}H NMR (500 MHz, CD_3OD)

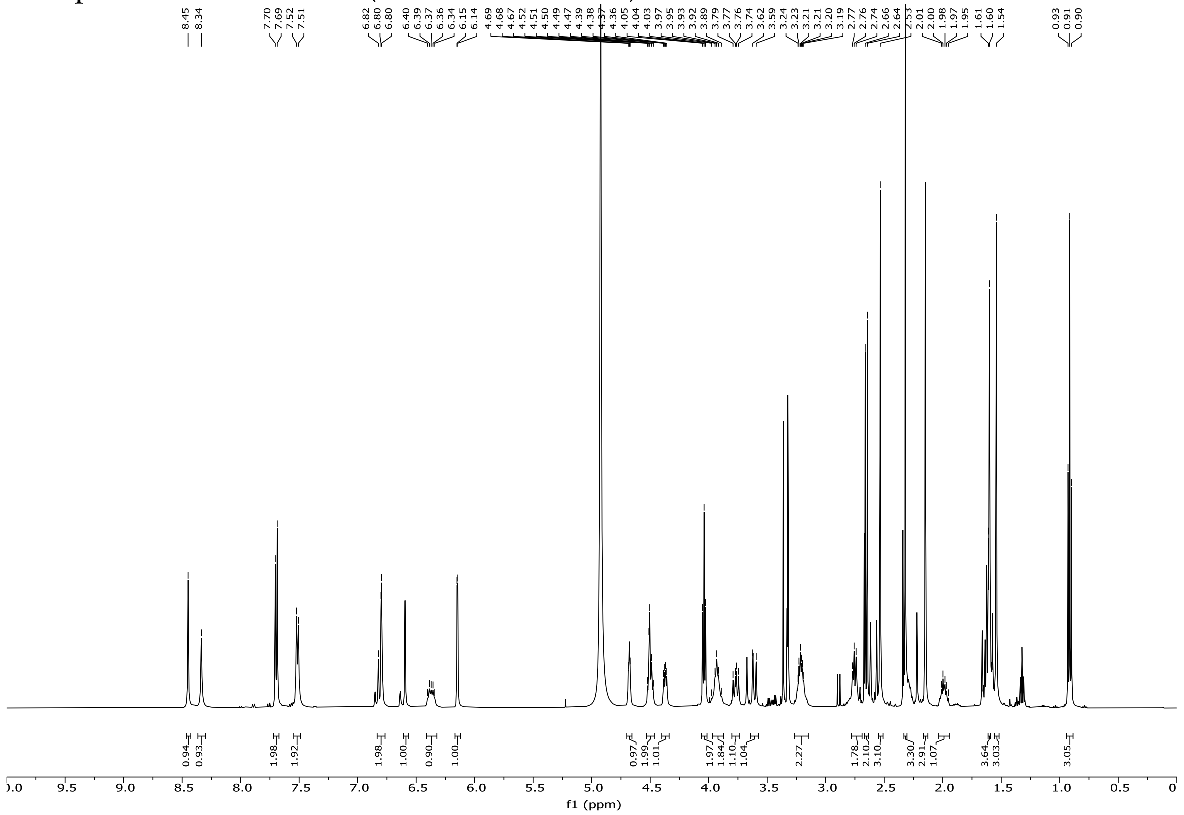

^{13}C NMR (126 MHz, CD_3OD)

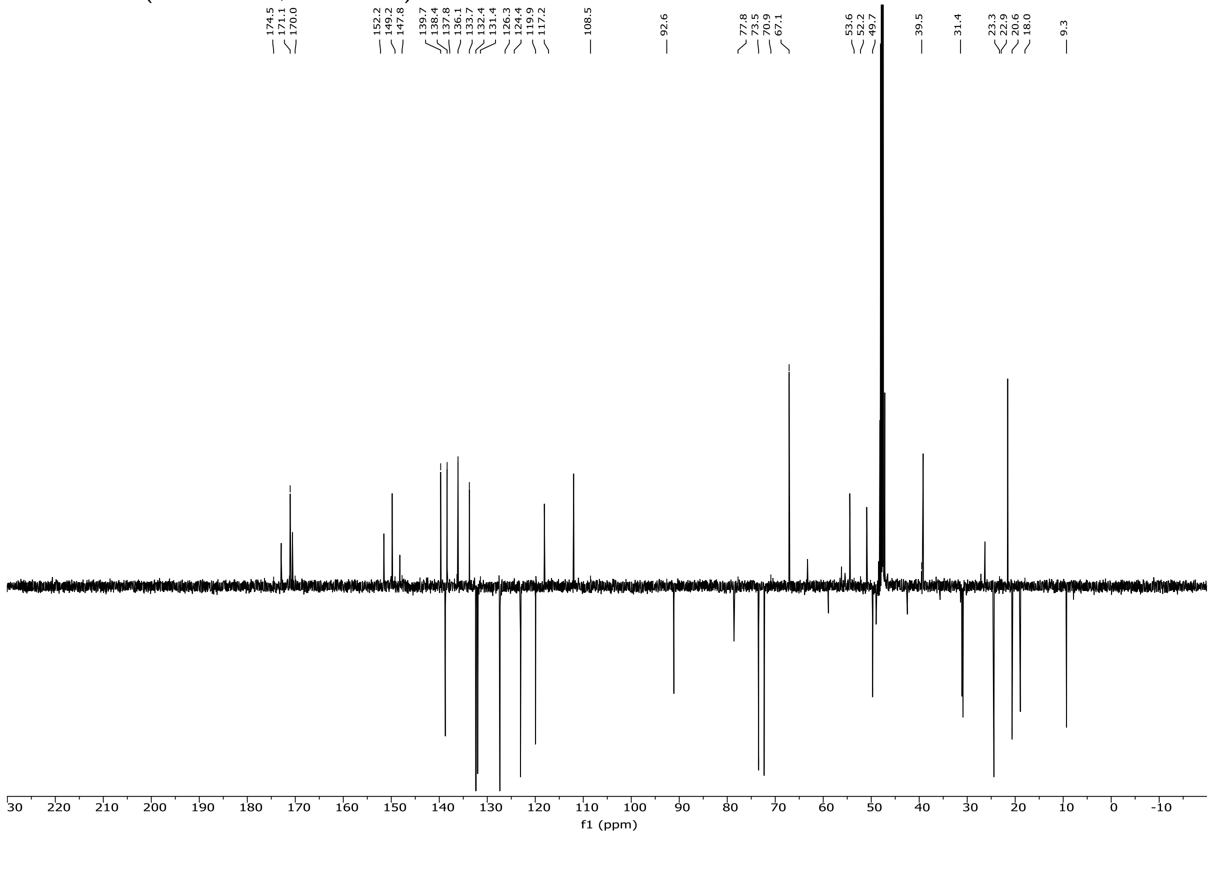

Compound **14e** ^{1}H NMR (400 MHz, CD_3OD)

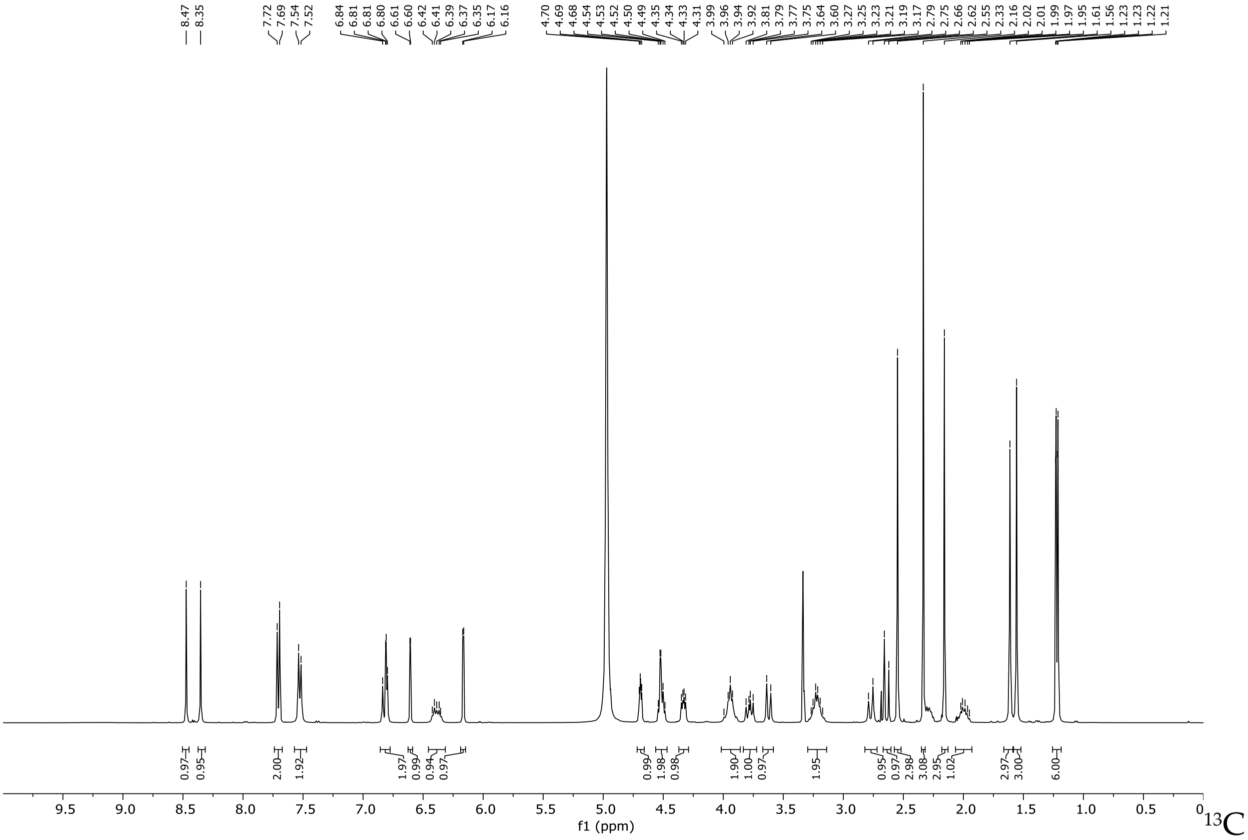

^{13}C NMR (101 MHz, CD_3OD)

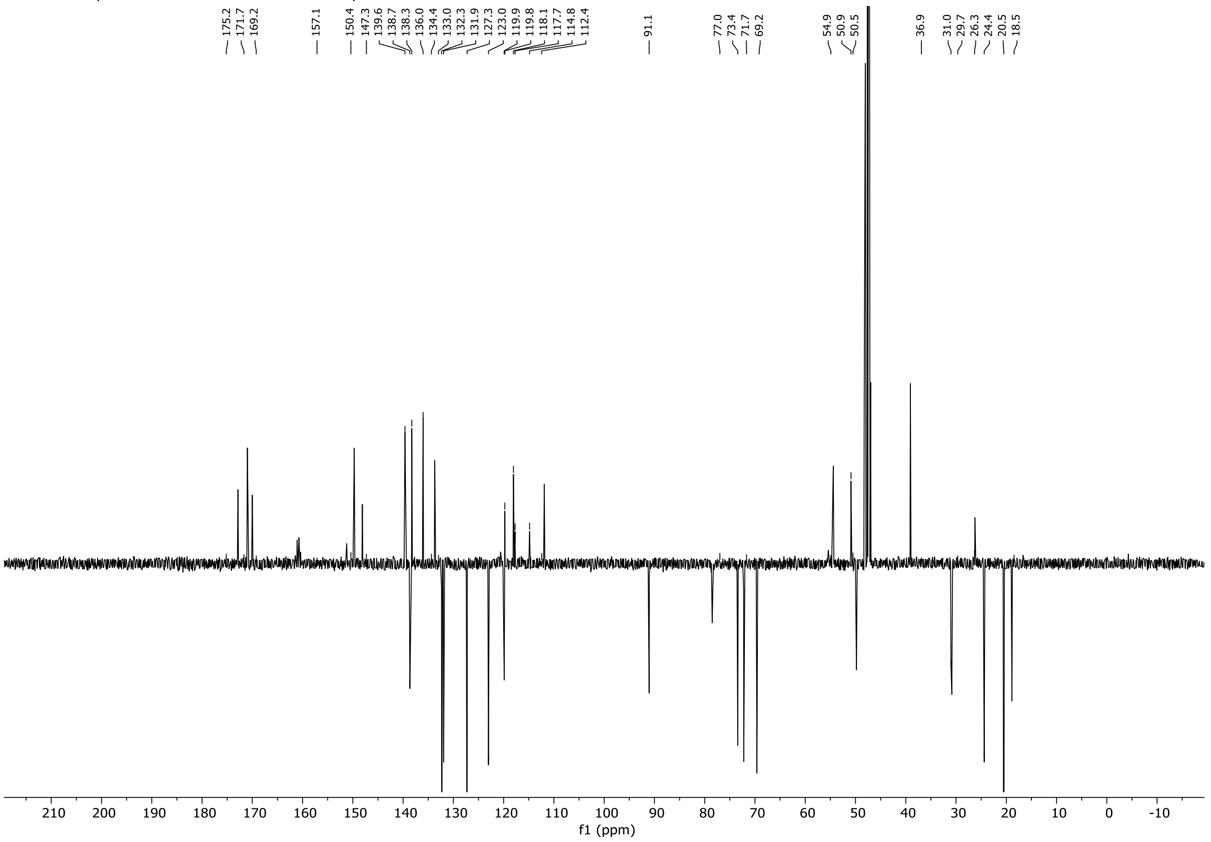

Compound **14f** ^{1}H NMR (500 MHz, CD_3OD)

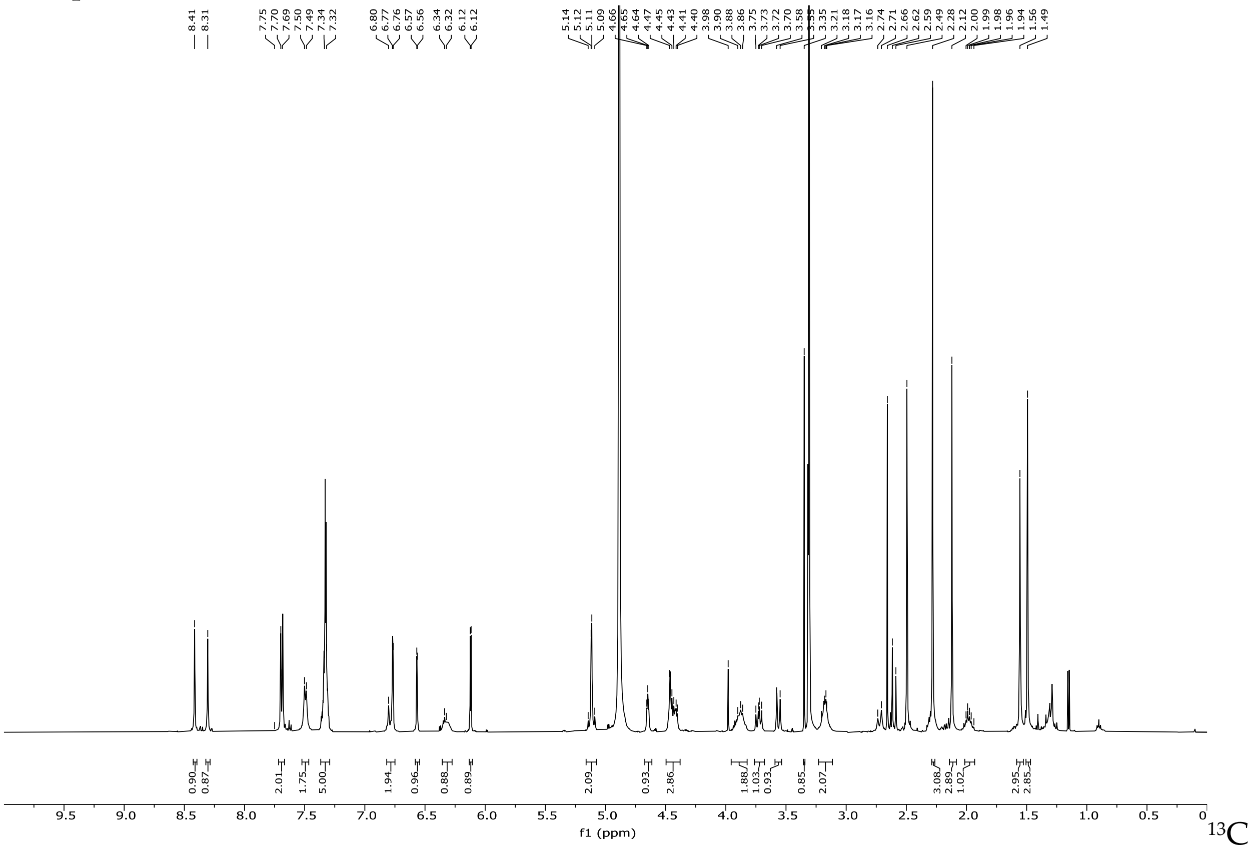

^{13}C NMR (126 MHz, CD_3OD)

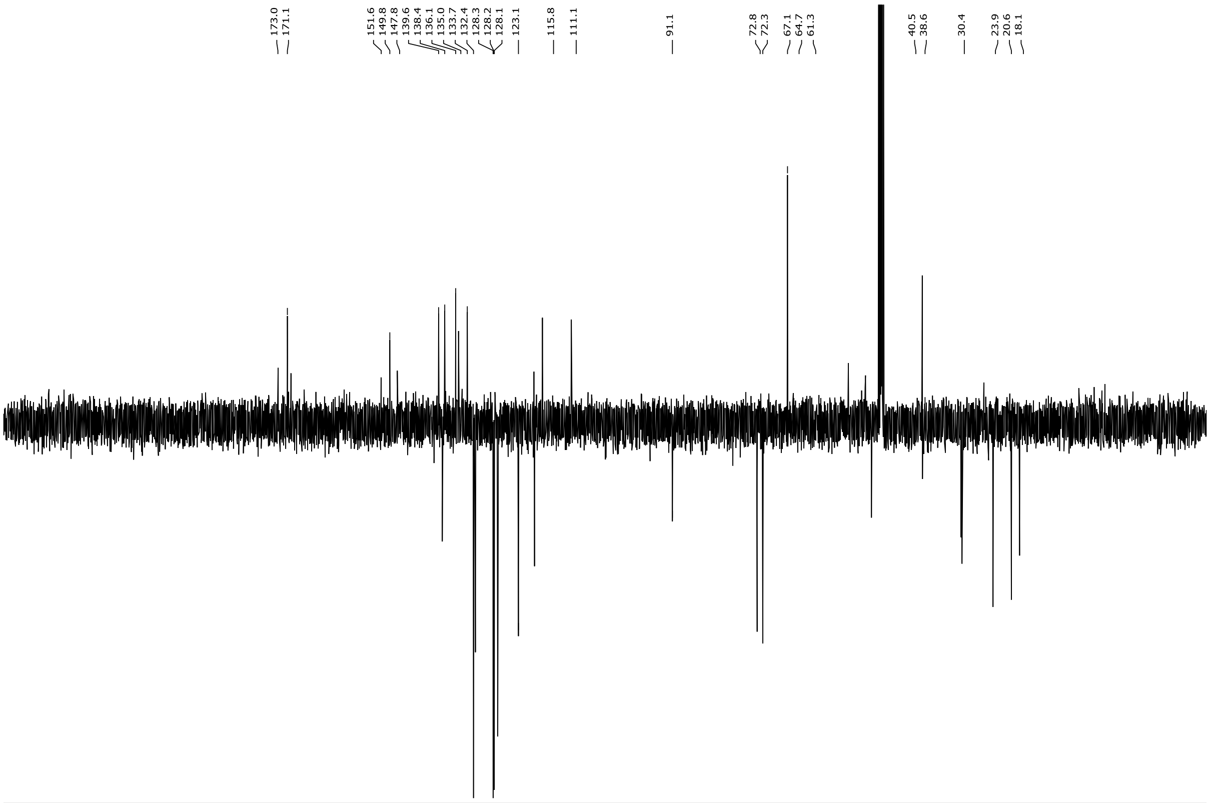

Supplement: Supplementary file 1 [file biomolecules-11-01357-s001.zip › biomolecules-1360553-supplementary.pdf]
